# *OSBPL10*, *RXRA* and lipid metabolism confer African-ancestry protection against dengue haemorrhagic fever in admixed Cubans

**DOI:** 10.1371/journal.ppat.1006220

**Published:** 2017-02-27

**Authors:** Beatriz Sierra, Petr Triska, Pedro Soares, Gissel Garcia, Ana B. Perez, Eglys Aguirre, Marisa Oliveira, Bruno Cavadas, Béatrice Regnault, Mayling Alvarez, Didye Ruiz, David C. Samuels, Anavaj Sakuntabhai, Luisa Pereira, Maria G. Guzman

**Affiliations:** 1 Virology Department, PAHO/WHO Collaborating Center for the Study of Dengue and its Vector, Pedro Kourí Institute of Tropical Medicine (IPK),Havana, Cuba; 2 i3S - Instituto de Investigação e Inovação em Saúde, Universidade do Porto, Porto, Portugal; 3 Instituto de Patologia e Imunologia Molecular da Universidade do Porto (IPATIMUP), Porto, Portugal; 4 Instituto de Ciências Biomédicas Abel Salazar (ICBAS), Universidade do Porto, Porto, Portugal; 5 Eukaryote Genotyping Platform, Genopole Pasteur Institute, Paris, France; 7 Vanderbilt Genetics Institute, Department of Molecular Physiology and Biophysics, Vanderbilt University School of Medicine, Nashville, TN, United States of America; 6 Functional Genetics of Infectious Diseases Unit, Pasteur Institute, Paris, France; 8 Faculdade de Medicina da Universidade do Porto (FMUP), Porto, Portugal; Purdue University, UNITED STATES

## Abstract

Ethnic groups can display differential genetic susceptibility to infectious diseases. The arthropod-born viral dengue disease is one such disease, with empirical and limited genetic evidence showing that African ancestry may be protective against the haemorrhagic phenotype. Global ancestry analysis based on high-throughput genotyping in admixed populations can be used to test this hypothesis, while admixture mapping can map candidate protective genes. A Cuban dengue fever cohort was genotyped using a 2.5 million SNP chip. Global ancestry was ascertained through ADMIXTURE and used in a fine-matched corrected association study, while local ancestry was inferred by the RFMix algorithm. The expression of candidate genes was evaluated by RT-PCR in a Cuban dengue patient cohort and gene set enrichment analysis was performed in a Thai dengue transcriptome. *OSBPL10* and *RXRA* candidate genes were identified, with most significant SNPs placed in inferred weak enhancers, promoters and lncRNAs. *OSBPL10* had significantly lower expression in Africans than Europeans, while for *RXRA* several SNPs may differentially regulate its transcription between Africans and Europeans. Their expression was confirmed to change through dengue disease progression in Cuban patients and to vary with disease severity in a Thai transcriptome dataset. These genes interact in the LXR/RXR activation pathway that integrates lipid metabolism and immune functions, being a key player in dengue virus entrance into cells, its replication therein and in cytokine production. Knockdown of *OSBPL10* expression in THP-1 cells by two shRNAs followed by DENV2 infection tests led to a significant reduction in DENV replication, being a direct functional proof that the lower *OSBPL10* expression profile in Africans protects this ancestry against dengue disease.

## Author summary

Dengue is a concern of worldwide health authorities given the increase on virus and vector dispersions. So far only one traditional GWAS survey has been performed in Vietnamese children. This disease is also epidemic in tropical and subtropical regions of the Americas, where most populations descend from a dynamic admixture between African, European and Native American backgrounds. Empirical evidence claimed that African descent was protective against dengue haemorrhagic phenotype in the Cuban population, and this study is the first to apply admixture mapping to identify candidate genes that confer African protection. We also present evidence that two candidate genes, *OSBPL10* and *RXRA*, are differentially expressed along dengue disease progression in Cuban patients and in a Thai dengue transcriptome dataset, and directly show that knockdown of *OSBPL10* gene expression leads to a significant reduction in DENV2 replication. A very important overall result of our work is that it provides a unifying framework for many genes that have been said to be protective in dengue. Our evidence places the LXR/RXR activation pathway at the center of natural dengue protection, and supports pursuing therapeutic techniques involving synthetic ligands of nuclear receptor genes or kinases inhibitors that interact with proteins involved in lipid metabolism.

## Introduction

Dengue is an emerging arthropod-born viral disease caused by the infection with any of the four dengue viruses (DENV-1 to 4). The virus is transmitted to humans by *Aedes aegypti* and *Aedes albopictus* mosquitoes. Morbidity and mortality associated with severe dengue infection render this disease a major increasing public health problem throughout tropical and subtropical regions. Dengue illness is also attracting awareness in Europe and in the United States as climate change and globalisation enlarge the geographic dispersion of the vector and the viruses [1]. A dengue infection can evolve from a subclinical infection, a relatively mild, self-limited infection known as dengue fever (DF), to the severe disease called dengue haemorrhagic fever (DHF), which may evolve to a life-threatening hypovolemic shock (dengue shock syndrome, DSS [2]). But only a small proportion of antibody-positive individuals develops DHF/DSS, while the vast majority suffers an asymptomatic infection or the mild disease. This differential susceptibility to disease severity indicates that besides immune factors, the host genetics may influence the infection outcome, acting in a complex interplay with viral and environmental factors. Diverse single nucleotide polymorphisms (SNPs) in genes such as *HLA-I*, *HLA-II*, *TNF-α*, *IL-10*, *TGF-β1*, *FcγRIIa*, *VDR*, *CD209* and *OAS* have been associated with symptomatic dengue or considered protective against the disease, in Asian and Latin American populations [3]. Also, *MICA* and *MICB* genes have been associated with susceptibility to dengue in Cuba [4], partially overlapping previous results reported in the only genome-wide association study (GWAS) performed so far in Vietnamese children, and showing significant association of *MICB* and *PLCE1* genes with DSS [5].

Evidence supporting the impact of human genetic factors on infection outcome also comes from differences between ethnic groups in developing severe DHF/DSS symptoms [6]. As early as in 1906, it was reported that Cuban dark-skinned individuals showed a remarkable resistance against dengue disease compared with light-skinned individuals [7]. This early observation was confirmed during the 1981 Cuban DHF/DSS epidemic of DENV-2 when ethnicity was recognized for the first time as a possible host risk factor, and confirmed afterwards in several other dengue Cuban outbreaks [8]. The low occurrence of dengue disease in Haitians [9] and in African populations [10] adds further support for this ancestry influence. A genetic characterization of 30 ancestry informative markers conducted in the Colombian population [11] confirmed the protective effect of African ancestry against severe dengue outcomes (odds ratios, ORs in 0.963–0.971 interval).

Till recently, population structure as occurs in admixed populations was a major confounding factor, requiring strategies for correction of the association p-values [12]. But admixed populations are a great advantage in cases of differential ancestry-conferred susceptibility/resistance to a disease through the use of admixture mapping [13]. The rationale of admixture mapping is that the ancestry blocks will be distributed at random across the genome, reflecting the admixture proportions of the parental ancestries, except in candidate gene locations where statistically significantly different proportions for the ancestry with higher disease levels will be observed in cases versus controls. It has been shown that this test is statistically more powerful than traditional GWAS [14]: around 250 samples can provide a 60% power to detect a two-fold risk due to ancestry, compared to the thousands of samples required in GWAS. In fact, because of the recentness of admixture, the typical ancestry blocks are significantly larger than haplotype blocks, thus lowering the multiple testing burden. This strategy has been successfully applied in African-Americans and Latin-Americans, in association with various diseases, such as asthma [15] and type 2 diabetes [16].

Cuba is advantageous for studies of ancestry-conferred DHF susceptibility/protection. The current Cuban population is mainly derived from the mix of two well-defined ancestral populations: European colonizers, who began to arrive in 1492 from the Iberian Peninsula, followed by other countries; and enslaved Africans, arriving in the 16^th^ century, mainly from West Africa. The contribution of the first aboriginal Cuban inhabitants, almost totally exterminated during the Spanish conquest, is almost negligible [17]. The Western and Eastern sides of the island show differences in historic settlement, with European descendants concentrating in the capital [17]. DENV experience has also been different among them. After an absence of 40 years, DENV1 was reported in 1977 and transmitted to nearly one-half of the Cuban population. Four years later, DENV2 (Asian origin) infected approximately 25% of the population, and a large DHF/DSS epidemic occurred. In 1997, another Asian DENV2 virus entered the country, producing a local epidemic in the municipality of Santiago de Cuba. In 2001, DENV3 (Asian genotype), was detected in Havana city [18]. In 2006, the circulation of DENV4 was reported in Havana, while DENV3 affected Guantanamo [19].

We conducted a GWAS of 2.5 million SNPs in 274 Cubans, including patients (DF and DHF) of the 2006 dengue epidemic, from Havana (west) and Guantanamo (east) cities, and in geographically matched asymptomatic individuals and population controls. The high level of admixture in Cuba enabled us to apply the first admixture mapping, thus facilitating the identification of candidate markers ethnically associated with dengue infection. Firstly, we conducted a global admixture analysis which allowed us to confirm the statistically significant decrease of African ancestry in the DHF cohort and to fine tune the association analysis in the admixed Cubans (cases and controls were paired to not differ more than 2% in African ancestry), whose statistical burden is 2.6x10^-8^. Secondly, we performed a local ancestry assignment along chromosomes which identified the precise regions (and genes) where the African ancestry was significantly higher in asymptomatic compared with DHF (above 3SD threshold in the difference in African ancestry between controls-cases). The fine-tuned association analysis can identify a few SNPs (located in a small chromosomal region) that confer reasonable individual risk/protection; the local ancestry assignment can identify ancestry-related blocks (medium to big chromosomal regions, depending on the time since admixture) containing SNPs conferring low-medium risk/protection, which would escape the association test. We also performed a functional test consisting in knocking down the expression of one candidate gene by shRNA, followed by DENV infection assays.

## Results

### Global ancestry influence in dengue infection outcome

**Fig 1:**
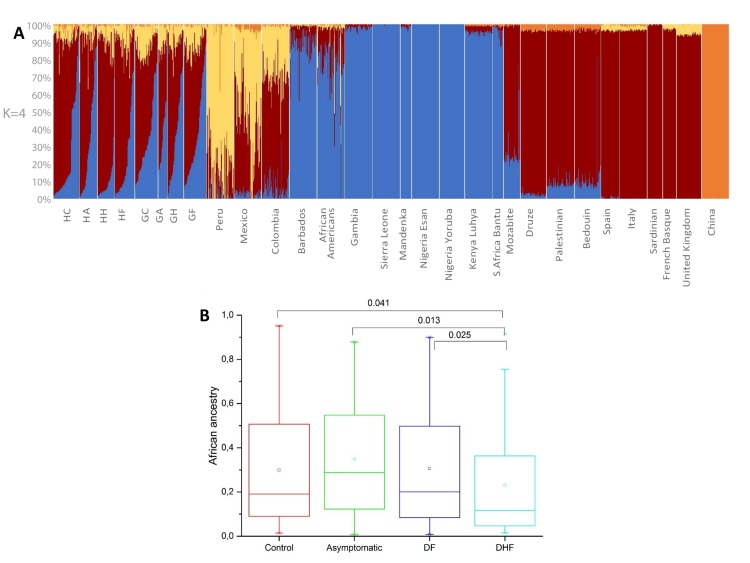
The global ancestry in Cuba and its influence on susceptibility to dengue. **(A)** ADMIXTURE results for four ancestral populations (blue component represents the European ancestry, red the African, yellow the Native American and Orange the East Asian). HC: Havana controls, HA: Havana individuals with asymptomatic infection, HH: Havana DHF cases, HF: Havana DF cases, GC: Guantanamo controls, GA: Guantanamo individuals with asymptomatic infection, GH: Guantanamo DHF cases, GF: Guantanamo DF cases. **(B)** Box plots for the African ancestry in the Cuban groups: controls; individuals with asymptomatic infection; DF (dengue fever); DHF (dengue haemorrhagic fever). The boxes represent the interquartile range and the whiskers are the 5% and 95% quartiles. The significant p-values for the two-tailed Wilcoxon rank-sum test between pairs of groups are displayed; non-significant ones are not displayed.

The genome-wide Cuban screening confirmed that all individuals in this study have a mixed ancestral composition. The main ancestry backgrounds (Fig 1A for K = 4) derive from Africa (represented by the blue colour) and Europe (red), but the range follows the entire spectrum of admixture, from nearly 0% African and 90% European to the inverse ratio, while the remaining 10% are from Native American (yellow) and East Asian (orange) influences. Comparing the population control groups from Havana and Guantanamo (HC and GC) as references for the two geographical regions, the average proportions of the African component are statistically different (25.2% and 35.3%, respectively; two-tailed Wilcoxon rank-sum test p = 1.43x10^-3^), identical to published values [20]. The Native American component was 6.5% in Havana and 13.5% in Guantanamo, values statistically significantly different (p = 1x10^-6^); while the East Asian component was of 1.9% and 0.7% respectively (p = 0.224). A finer description of Cuban ancestry is presented in Supplementary section 1.2 (Figs A-C in S1 Text). This includes confirmation that, despite the statistical differences in the African/European components between Havana and Guantanamo, the sub-structures within those two components in the two cities are identical, not favouring differential migration events into the two parts of the island (Figs D-E in S1 Text).

Focusing on the global ancestry among the Cuban cohorts (Fig 1B), the average African ancestry is significantly lower in DHF (22.9%) when compared with DF, controls and especially asymptomatic groups (30.6%, p-value = 0.025; 30.0%, p-value = 0.041; 34.7%, p-value = 0.013, respectively). These results confirmed that African ancestry is protective against DHF phenotype in Cuba, and the odds ratios are very similar to the ones reported in Colombia [11] (Table 1). Nevertheless, the evidence of ancestry influence in dengue was not so straightforward when the samples were divided according to the city of origin: in Havana, the African ancestry is even more significantly lower in DHF (10.3%) compared with DF, controls and especially asymptomatic (24.4%, p-value = 0.009; 25.2%, p-value = 0.015; 33.0%, p-value = 0.002, respectively) and OR even more protective (Table 1); while in Guantanamo, the African averages are statistically identical between all groups (35.3% in controls, 38.1% asymptomatic, 36.4% DHF and 36.0% DF). This complex relation was confirmed by an iterative model (Fig F in S1 Text). Our data show that there is an African protection conferred against DHF in Cuba, but other currently unknown confounding factors render it a complex relation, even in such a geographical restricted scenario as Cuba.

**Table 1:** Odds ratios of the African ancestry influence in DHF phenotype when compared to asymptomatic subjects, in Cuba in general, only Havana city and in Colombia.

	Odds ratio		
	1% African ancestry	50% African ancestry	100% African ancestry
Cuba	0.979	0.396	0.151
Havana	0.920	0.045	0.012
Colombia*	0.962	0.204	0.042

* From [11].

### Fine-matched corrected population structure followed by association evaluation

**Fig 2:**
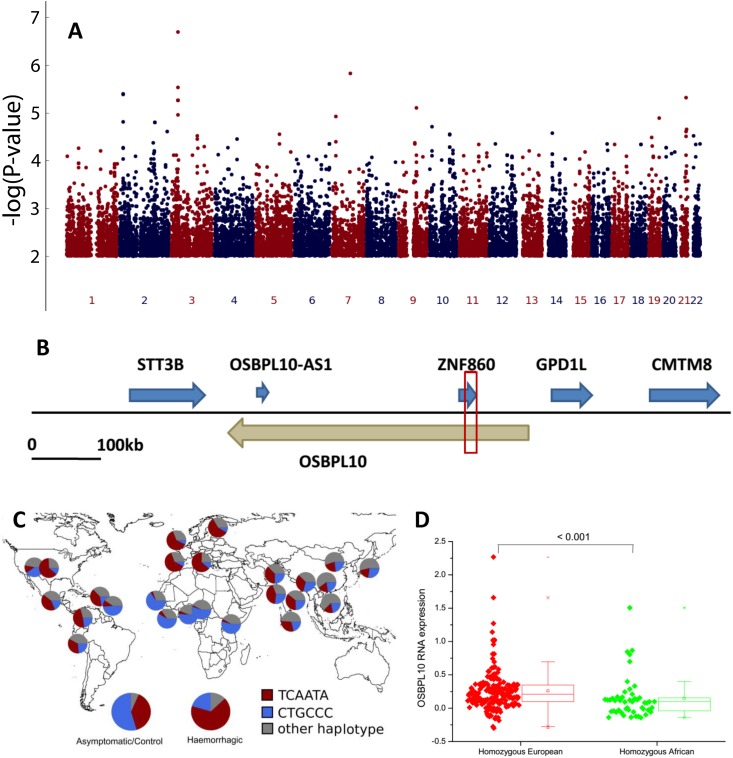
The relevant region on chromosome 3 containing the *OSBPL10* gene **(A)** Manhattan plot for the association analysis in the 54 fine-matched population structure corrected Cuban pairs of asymptomatic/control versus DHF subjects. **(B)** The region on chromosome 3, with the haplotype defined by the six significantly associated SNPs indicated by the red box. Genes on the forward sense are indicated in blue; genes on the reverse sense are indicated in light brown. **(C)** Worldwide frequency of the African (blue), European (red) and other (grey) *OSBPL10* haplotypes for populations of the 1000 Genomes project, and also for asymptomatic/control and DHF in Cuba. **(D)** mRNA expression for homozygous genotypes for African and European *OSBPL10* haplotypes in the 1000 Genomes project transcriptome information.

The fine-matched corrected association tests (Tables C-E in S1 Text) identified the lowest p-values (10^−6^–10^−7^) in the DHF comparison (HCG–haemorrhagic comparison group) for six highly linked (Fig 2A; Table I and Figs N-P in S1 Text) SNPs extending for 8,370 bps in chromosome 3, in a region containing the *OSBPL10* (oxysterol binding protein-like 10) gene (Fig 2B). These p-values are close to the significant cutoff of 10^−8^. OSBPL10 protein is involved in lipid transport and steroid metabolism. Curiously, the most frequent haplotypes in African and European populations consist totally of the alternative alleles for all six SNPs, and attain frequencies higher than 50% in each continent (Fig 2C). The odds ratio calculated in DHF is 0.25 [95% CI 0.13–0.47] for the African haplotype (Table 2).

**Table 2:** Odds ratios of the African *OSBPL10* haplotype and *RXRA* alleles in DHF when compared with asymptomatic/control and tests (identified by Y) where statistical significant evidence was detected for each gene.

Gene	Haplotype SNP	Position	Haplotype allele	Odds ratio	OR 95% confidence interval	A	B	C	D	E	F
*OSBPL10*	African haplotype rs4600849 rs11129475 rs6419811 rs11718700 rs975406 rs7639637	32027672 32030544 32031135 32033248 32035587 32036042	CTGCCC	0.25	[0.13–0.47]	Y		Y	Y	Y	Y
*RXRA*	rs12339163	137205188	G	0.36	[0.17–0.77]		Y		Y	Y	
	rs62576287	137214888	C	0.10	[0.01–0.83]						
	rs3118593	137426334	A	0.44	[0.25–0.77]						
	rs4262378	137515156	G	0.41	[0.24–0.72]						
	rs4424343	137515158	A	0.43	[0.24–0.76]						

A—Association; B—Admixture mapping; C—Positive selection (XP-EHH and iHS); D—Expression changes in Cuban patients RT-PCR; E—Expression changes in Thai transcriptome dataset; F—Expression changes between African and European haplotypes/genotypes in 1000 Genomes transcriptome.

By using the 1000 Genomes transcriptome [21], we confirmed that the homozygous individuals for the *OBSPL10* African haplotype have a significantly reduced (by half) expression (mean value = 0.145) when compared with the homozygous for the European haplotype (mean value = 0.257; p<0.001; Fig 2D). This indicates that the haplotype affects mRNA expression. We further ascertained that these non-coding SNPs are not located in the inferred promoter, but they can be related with weaker enhancer regions and are recognised by many transcription factors, including *STAT* and *RXRA* (Fig Q in S1 Text). Besides the haplotype region, the segment immediately 5’ to it (within the gene) has some SNPs with significant p-values in the association test that are also regulatory regions in several cell types (Table O and Fig R in S1 Text). Although there is no current good database to check eQTLs in African populations, we used GTEx despite its highly European-biased dataset (84.3%). The six identified *OSBPL10* SNPs are not identified in GTEx as eQTLs, but surveying the eQTLs located on that chromosomal region, there is one with frequency differences between African and European populations (rs7642435, at position 32036787; 1000 Genomes frequency in Africa–A = 0.092, G = 0.908; 1000 Genomes frequency in Europe–A = 0.723, G = 0.277), which presents alternative homozygous genotypes associated with our African/European haplotypes: “African” haplotype with rs7642435_GG and “European” haplotype with rs7642435_AA. As can be confirmed in Fig S in S1 Text, rs7642435_GG has a significant reduction of *OSBPL10* expression compared with rs7642435_AA, and this SNP is predicted to have an eQTL effect (posterior probability>0.9) in several tissues: artery (aorta, coronary and tibial), brain (nucleus accumbens and caudate), liver, adrenal gland, pancreas, esophagus (muscularis and gastroesophageal junction), stomach, nerve and cells-transformed fibroblasts. The remaining eQTLs in the region are almost fixed in both African and European populations. Possibly, our haplotype association is identifying the rs7642435 eQTL, although more information on African eQTLs is needed in order to ascertain other possible eQTLs in the *OSBPL10* region.

This extended *OSBPL10* segment (haplotype and immediate 5’ region) has probably been under positive selection in the African Yoruban population, as inferred in the Haplotter tool based on HapMap dataset (iHS measure; Fig T in S1 Text). The positive selection of *OSBPL10* gene was confirmed in Cuban HCG when applying XP-EHH measure (Fig M and Tables M-N in S1 Text). Therefore, it seems that the *OSBPL10* haplotype and its 5’ region, although non-coding, bear several expression regulatory SNPs with large frequency differences between Africans and Europeans, probably due to selection events.

Several other SNPs located on genes (mostly one or two SNPs per gene) reached association p-values of 10^−5^. By checking which SNPs are more frequent in Africa (information from the 1000 Genomes database) and which genes would have differential gene expression between dengue patients and control/convalescent subjects (in the whole blood transcriptome obtained in a Thai dengue dataset [22] and using a linear discriminant analysis effect size method, LEfSe [23], for high-dimensional class comparisons), besides *OSBPL10*, a few kinases or kinase-related genes are amongst the most significant genes (Fig G in S1 Text): *CAMK1D* (calcium/calmodulin-dependent protein kinase ID), *MAPKAPK5* (mitogen-activated protein kinase-activated protein kinase 5), *PIK3AP1* (phosphoinositide-3-kinase adaptor protein 1), *SNRK* (sucrose nonfermenting related kinase), *GNA14* (guanine nucleotide binding protein (G protein), alpha 14) and *DAB-1* (dab, reelin signal transducer, homolog 1 (*Drosophila*)).

The non-African set (Fig H in S1 Text) has more variable and generalized functions, dealing with hemostasis (*DOCK10*), phospholipid binding (*PLEKHM1L*), protein serine/threonine kinase activity and ribosomal protein S6 kinase activity (*RPS6KA2*), protein homodimerization activity and HMG box domain binding (*OLIG2*), sequence-specific DNA binding transcription factor activity and chromatin binding (*TSHZ3)*.

### Fine-matched corrected population structure followed by admixture mapping

The admixture mapping led to the identification of locally enriched African regions across the genome in the asymptomatic/controls (Figs I-K and Tables F-H in S1 Text). One of these regions is located on chromosome 9 (detected at HCG and FCG/fever comparisons), containing the *RXRA* (retinoid X receptor alpha), *COL5A1* (collagen type V alpha 1) and *FCN2* (ficolin (collagen/fibrinogen domain containing lectin) 2) genes. We confirmed from the expression data on Thai dengue patients [22] that only *RXRA* has statistically significant altered expression (Fig L in S1 Text), supporting several lines of evidence linking retinoid receptors with infectious diseases and dengue [24]. Detailed description of other significant regions is presented in supplementary material sections 1.5 and 1.6 (Tables K-L in S1 Text).

**Fig 3:**
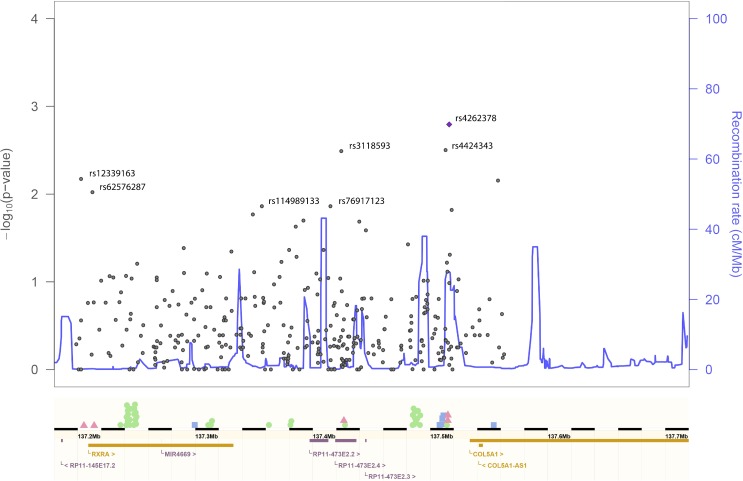
The *RXRA-COL5A1* region with the most significant SNPs highlighted. Map obtained with the LocusZoom tool, using recombination rate information from the Yoruba reference population. The symbols above the rule represent significant SNPs, for the Cuban data (the pink triangles), the African comparison between individuals having low (n = 6; lower than 10 RPKM) and high (n = 8; higher than 20 RPKM) *RXRA* expression (green circles), the same for European individuals (n = 42 and n = 39, respectively; blue squares).

We checked the SNPs with significant association p-values in the *RXRA*-*COL5A1* region in the Cuban HCG (Fig 3; Table J in S1 Text), and verified that two locations have higher significant p-values and OR between 0.103–0.436 (Table 2). The most significant p-values are for three intergenic SNPs (rs4262378, rs4424343 and rs3118593) located in inferred enhancers (Table P in S1 Text). The other location is immediately before and in the beginning of *RXRA*, and contains SNPs rs12339163 and rs62576287 (the latter only exists in Africa with a 9% frequency) placed in poised and weak promoters and weak enhancers. From these various significant SNPs, only rs3118593 is within a promoter and a lncRNA (*RP11-473E2*.*4*; Fig 3), and interestingly, according to the GTEx portal, the patterns of expression for *RXRA* and *RP11-473E2·4* genes in the various human tissues are totally opposite, with *RXRA* being mainly expressed in liver, muscle, skin and whole blood while almost not expressed in brain and testis, and the other way around for *RP11-473E2·4* (Figs U-W in S1 Text). The opposite expression pattern between the *RXRA* and *RP11-473E2·4* genes raises the possibility that the lncRNA silences the expression of *RXRA*. When checking the *RXRA* mRNA expression for the typical African and European genotypes in those SNPs (based on the 1000 Genomes transcriptome from lymphoblastoid cell lines), no statistical differences were observed (data not presented). We further performed association tests for the whole *RXRA-COL5A1* region in the 1000 Genomes transcriptome comparing *RXRA* high- and low-expression groups within Africans and Europeans. The African test shows that there are 37 significant (at the 1% level) SNPs surrounding *RXRA* gene, including rs3118593, that are differently frequent between the high- with low-expression groups, and these can be regions for expression regulation of the gene (Fig 3; Table Q in S1 Text). While in Europe, the number of significantly differentiated SNPs is much lower, only six (Fig 3; Table R in S1 Text). A reasonable hypothesis would be that the advantage in Africans is related with a faster control of *RXRA* expression.

### *OSBPL10* and *RXRA* expression in dengue patients and focused enrichment analysis

**Fig 4:**
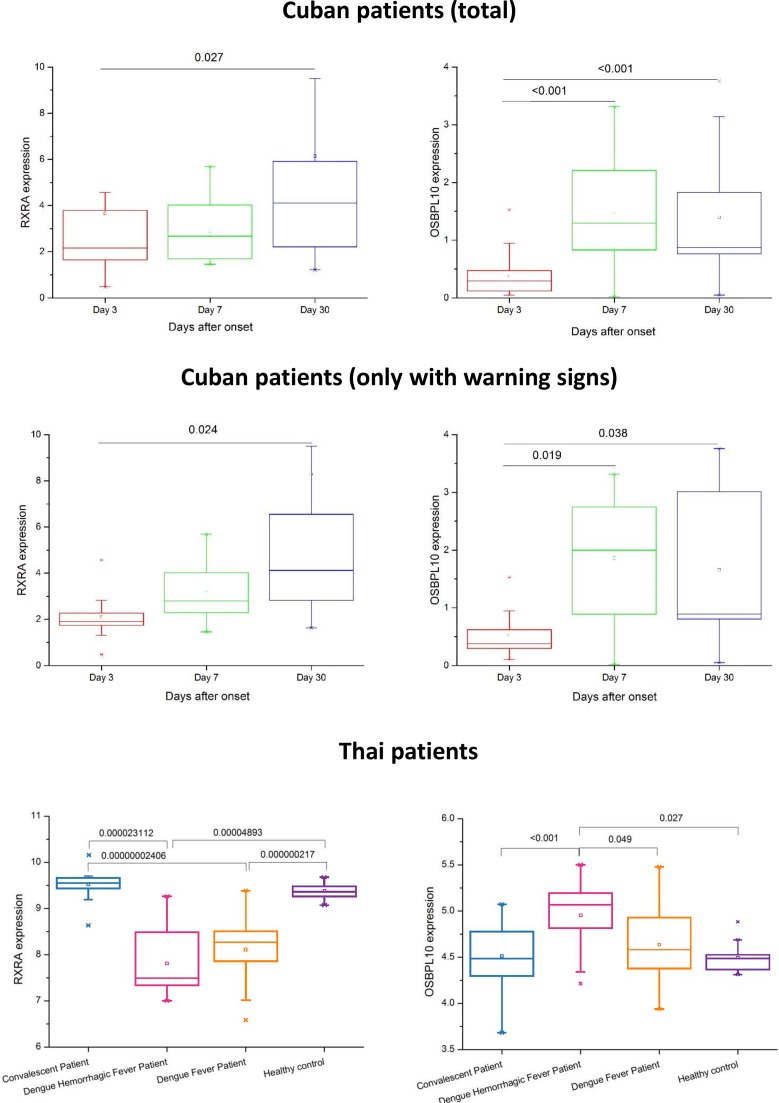
Gene expression for *RXRA* and *OSBPL10* in Cuban dengue patients along the course of disease Data is shown for all Cuban patients, Cuban patients with warning signs, and the Thai transcriptome dataset for whole blood. [22]

The genetic evidence collected in this work, summed up in Table 2, for the involvement of *OSBPL10* and *RXRA* genes in the protection against DHF, led us to check their expression in Cuban patients throughout the infection process (Fig 4). The mRNA expression of *RXRA* was significantly higher during convalescence (day 30) compared to day 3 (p = 0.027), and also higher than at day 7 after fever onset (not significant). There were no significant differences between days 3 and 7 after fever onset. These results are comparable with the ones for the Thai dataset, where *RXRA* expression is significantly decreased in DF and DHF cohorts when compared with controls and convalescent, indicating that this gene expression is decreased along the disease course, and only returns to normal values in convalescence. The differences between DF and DHF cohorts did not reach statistical significance, what can be explained by the small sample size.

The mRNA expression of *OSBPL10* was significantly increased by day 7 and in convalescence, when compared with day 3 (p<0.001 for both). There is a decrease between day 7 and convalescence, but it is not significant. In Thai, the DHF group has a significantly higher *OSBPL10* expression compared with all other groups. Considering both datasets, it seems that *OSBPL10* expression is very low in the acute phase, increases significantly at the end of the acute phase, and decreases again in convalescence. For both genes, results in Cuban patients showing warning signs were similar to the ones observed in the totality of patients. These results show that both gene expressions are altered along the dengue disease progression.

We further checked which pathways could have both genes acting together (in Ingenuity database; https://targetexplorer.ingenuity.com/index.htm), and found that they play a central role in the LXR/RXR activation pathway, related to cholesterol metabolism and cytokine production in macrophages (Fig X and Table S in S1 Text). We performed a gene enrichment pathway analysis in the Thai dengue transcriptome in whole blood as a surrogate of the LXR/RXR interaction pathway in macrophages. Unfortunately, there are no robust transcriptome for hepatocytes, in which the same pathway occurs without the cytokine production. Results for comparisons of DHF and DF versus convalescent and controls (Figs Y-AA in S1 Text) showed that the lipid metabolism set of genes is always significantly upregulated in patients, and the main contributing genes are *OSBPL10*, *LDLR* and *MSR1*, the two last mediating the entrance of LDL (low-density lipoprotein) to the cell. *NF-kB* expression is always upregulated in the convalescents (significantly in DHF comparison), or, biologically more meaningful, downregulated in patients, with main genes been *RXRA* and *NF-kB*. The LXR/RXR activation set of genes is never statistically significant, but the most contributing genes are *NR1H3/LXRA*, *ABCA1*, *ARG2* and *NCOR1* up-regulated in DF, and *ABCG1*, *LPL* and *RXRA* down-regulated in DHF. This analysis reinforces the importance in dengue disease of the enlarged pathway for LXR/RXR activation, including the cholesterol/lipids metabolism and the NF-kB control of cytokines. We demonstrated that the African protective genes *OSBPL10* and *RXRA*, which play central roles in this pathway, are always identified as top differentially expressed genes.

### Effect of *OSBPL10* knockdown on DENV replication

**Fig 5:**
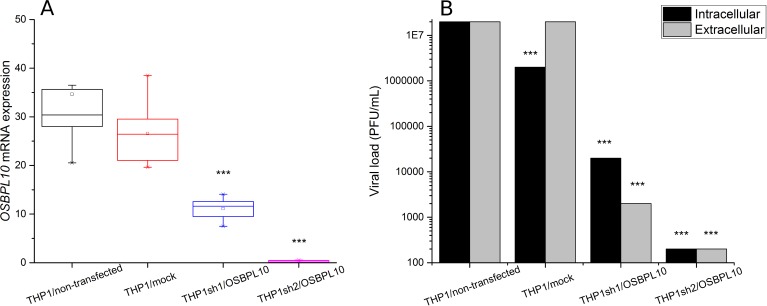
Functional assay of *OSBPL10* knockdown and its effect on DENV-replication **(A)** Box plots for the *OSBPL10* mRNA expression in the various THP1 cells (non-transfected and transfected with mock, sh1 and sh2 plasmids). The boxes represent the interquartile range, the little square the mean, the line the median and the whiskers are the 5% and 95% quartiles. The *** symbol indicates that the two-tailed t-test p-values for comparisons between THP1sh1/OSBPL10 and THP1/non-transfected or THP1/mock, as well as for THP1sh2/OSBPL10 and THP1/non-transfected or THP1/mock are significant. **(B)** Viral load (log10 of PFU/mL), measured intracellularly and extracellularly, in the various THP1 cells (non-transfected and transfected with mock, sh1 and sh2 plasmids) when infected by DENV2. The *** symbol indicates that the two-tailed t-test p-values for pairwise comparisons (THP1sh1/OSBPL10 vs. THP1/non-transfected or THP1/mock, THP1sh2/OSBPL10 vs. THP1/non-transfected or THP1/mock) are significant.

The two shRNA plasmids used to induce *OSBPL10* knockdown in the THP-1 cell line were very efficient, as measured by mRNA expression (Fig 5A). In fact, no significant differences in the *OSBPL10* expression were observed among THP1/non-transfected and THP1/mock (mean values of 34.62 and 26.58, respectively; two-tailed t-test p = 0.15). While a noteworthy reduction in *OSBPL10* expression levels was observed in THP1sh1/OSBPL10 assay (mean of 11.11; p = 0.00022 and p<0.00001, compared with THP-1/non-transfected and THP1/mock, respectively), and an almost complete non-expression in THP1sh2/OSBPL10 assay (mean of 0.30; p<0.00001 and p<0.00001, compared with THP-1/non-transfected and THP1/mock, respectively).

DENV2 infection assays in these OSBPL10-knockdown cell lines provides robust evidence that down-modulation of *OSBPL10* affects in a direct way DENV replication (Fig 5B). Significant (p<0.00001) viral load reductions were observed intracellularly in both OSBPL10 knockdown cell lines (mean values of 2x10^7^ in THP1/non-transfected, 2x10^6^ in THP1/mock, 2x10^4^ in THP1sh1/OSBPL10 and 2x10^2^ in THP1sh2/OSBPL10), as well as extracellularly (mean values of 2x10^7^, 2x10^7^, 2x10^3^ and 2x10^2^, respectively).

## Discussion

Human populations are structured in three main groups, African, European and Asian. The independent selective pressures acting upon these groups can lead different genes to be selected in adaptation to the same pathogen, and those genes can interact in a common crucial pathway or in different pathways of additive importance to the disease process.

The current study supports the African-ancestry-conferred resistance to DHF, in comparison with European background, concurring with the report in Colombia [11]. It is unlikely that DENV has exerted this selective pressure, since its associated mortality is low and it does not alter reproduction. But yellow fever virus, another African-originated flavivirus, which has a remarkable mortality level (being 6.8 times higher in Caucasians), could have generated protective genetic variants against itself, hepatitis C virus (HCV) and DENV [25]. It has been demonstrated that besides infection-related genes, metabolic genes are also under intense selective pressure, and both can interact as in the case of the low-activity alleles of glucose-6-phosphatase dehydrogenase providing reduced risk to malaria infection [26]. It is known that Africans have a lower atherogenic lipid profile, characterized by low triglyceride (T), total cholesterol, and LDL levels, and high high-density lipoproteins (HDL) levels, when compared to Europeans [27]. Signs of selection were already identified in West Africans for *APOL1* and *CD36* genes involved in lipid metabolism and probably driven by pathogen resistance [28]. Our evidence adds two new genes to the differential lipid profile between Africans and Europeans, which play a role in infectious resistance. We detected signs of positive selection on the *OSBPL10* gene, while balancing selection may have generated additional polymorphisms regulating *RXRA* expression in Africans.

A direct link between dengue infection progression and lipid profile changes has been made, so that lipids may be used as predictors of clinical outcome [29]: increased T and HDL and decreased LDL were observed in severe dengue. Recent metabolic and lipid profiling in serum samples from dengue cohorts [30, 31] have reinforced these earlier findings, showing that DENV infection causes temporary changes in metabolites involved in acute inflammatory responses, with major perturbed metabolic pathways including fatty acid biosynthesis, fatty acid beta-oxidation, phospholipid catabolism and steroid hormone pathway, and that progression into DSS is associated with certain metabolites (phosphatidylcholines, diacylglycerol, phosphatidic acid, phosphatidylserine, triglycerides, and diacylglycerophosphoglycerol) that may act in endothelial cell homeostasis and vascular barrier function. Definitely, lipids are essential to DENV entrance and replication [32]. Flavivirus RNA synthesis and replication occur on an extended network of modified endoplasmic reticulum (ER) membranes, and then maturation takes place in the ER-Golgi complex [33]. These are the main cellular components where OSBPs play a key role on cholesterol and other sterols homeostasis [34]. Our functional assay clearly shows that knockdown of *OSBPL10* expression reduces significantly DENV replication, a direct proof that the lower *OSBPL10* expression profile in Africans protects this ancestry against dengue disease. Interestingly, replication of HCV and poliovirus has been shown to be dependent on OSBP in a PI4-kinase dependent manner, but DENV did not, possibly due to its ligand being PI3P and not PI4P [35]. This evidence and our detected African-related signs of association with kinases in Cuba calls for future research to be conducted in this group of enzymes.

If the *OSBPL10* gene is involved in signaling and transport of lipids, *RXRA* plays a role in the second part of the cholesterol homeostasis through the LXR/RXR activation pathway, in hepatocytes and macrophages. When cholesterol is in excess, as occurs in infection, the consequent lowering of its cellular concentration is reached by oxysterol and desmosterol binding to *LXR*, which forms heterodimers with *RXRA* in order to control the transcription activation of many genes regulating lipid metabolism. In macrophages, *RXRA* also regulates the integration of immune functions, thus defining the clinical outcome of dengue infection. Indeed, macrophages are key DENV target cells and also the principal source of pro-inflammatory mediators linked to severe dengue disease. LXR/RXRA heterodimers and sumoylated LXR inhibit the *NF-kB* transcription factor complex, which *per se* acts upon inflammatory mediators [36]. But a negative control of LXR/RXRA dimers is exerted by *IRF3*, which is activated by viruses and bacteria that entered the cell through *TLR3* and *TLR4* receptors, deregulating the cholesterol control and allowing the action of *NF-kB* transcription factor. It has been demonstrated that during dengue infection [24] there is down-regulation of *RXRA* expression in an *IRF3*-dependent manner in the host cell, to achieve optimal *IFN* expression. After infection, *RXRA* expression resumes, suppressing the type I IFN induction. Therefore, *RXRA* not only maintains the basal type I IFN and modulates the host antiviral response, but also regulates the antiviral inflammatory response.

Remarkably, our results offer a comprehensive explanation for various independent observations made in association with dengue. *RXRA* forms heterodimers with *VDR*, previously identified as dengue protective in Vietnamese [37]. The RXRA-VDR heterodimers negatively control the expression of several immune function genes. Also, *PLCE1*, protective against DSS in Vietnamese children [5], interacts with *RXRA* in the PPARA/RXRA activation pathway, positively controlling the expression of genes again related with lipid metabolism. Perhaps flavivirus resistance arose in Asians through *VDR* and *PLCE1* selection, while *OSBPL10* and *RXRA* selection provided resistance in Africans, all related with central lipids and cytokines pathways. Even the immune system genes associated with dengue illness, such as *TNF-α*, *IL-10*, *TGF-β1*, *FcγRIIa* and *CD209* [3], can also be related with the *RXRA* gene in several pathways, as their expression is controlled by dimers formed by RXRA and other nuclear factors.

Even the signals we detected in the kinases or kinase-related genes contribute to integrate our results in the general picture. *CAMK1D* is associated with chemokine, thrombin signalling and xenobiotic metabolism (through RXRA-CAR dimers), activation of neutrophil cells and apoptosis of erythroleukemia cells; *MAPKAPK5*, that responds to cellular stress and pro-inflammatory cytokines, and the use of a specific inhibitor of this gene blocked DENV assembly [38]; *PIK3AP1* which may interact with *OSBPL10*, as it is possible that this gene is activated by phosphatidylinositol-3-phosphate (PI3P) [39], and links Toll-like receptor signaling to PI3K activation preventing excessive inflammatory cytokine production; *SNRK* possibly expressed in the liver secretome; *GNA14* activates PLC (detected in the Vietnamese dengue GWAS) protein that generates diacylglycerol which further activates *PPARA* to forms heterodimers with *RXRA*, positively controlling the expression of genes related with lipid metabolism;[40] *DAB-1* may play a role in PI3K binding.

The genomic and functional confirmation of the protection conferred by *OSBPL10*, *RXRA* and related lipid metabolism against dengue illness supported in this study points out potential therapeutic applications. The generation of synthetic ligands of *LXR* should be pursued [41], given its central role in dimerization with *RXRA*. The possible PI3P-dependent activation of *OSBPL10* could be tested through the various PI3K inhibitors being developed in cancer treatment [42]. In addition, the African protection against DHF through kinases supports pursuing the development of kinase inhibitors as they seem to be able to block DENV assembly [38].

## Materials and methods

### Samples for GWAS genotyping

Havana is the capital of the country and is located in the West with 2 million inhabitants, while Guantanamo city is located in the eastern part with over 200 thousand inhabitants. Case samples from the two cities, collected during the 2006 dengue outbreak and classified according to WHO [43], were included: 67 subjects from Havana with confirmed dengue infection by DENV 4, 36 clinically classified as DF and 31 as DHF; and 70 subjects from Guantanamo with confirmed dengue infection by DENV 3, 41 classified as DF and 29 cases as DHF. Dengue infection was confirmed by dengue IgM detection in serum collected at day 6 of fever onset, as well as virus isolation in *Aedes albopictus* cell line and RT-PCR in samples collected in the first four days of fever [44, 45].

A screening for identifying dengue asymptomatic cases was conducted during the peak of the 2006 outbreak. Healthy adult individuals, without any dengue clinical symptoms, relatives or neighbours of dengue confirmed patients from three neighbourhood blocks with a high dengue incidence, were daily visited and checked for clinical dengue symptoms over 15 days. The ones that remained symptom free and were PCR or IgM positive were considered as asymptomatic cases. Thirty two asymptomatic individuals from Havana and 16 from Guantanamo, who were unrelated to the patients included in the genotyping, were added. Forty seven samples from healthy blood donors from Havana and 42 from Guantanamo were included as population controls.

Havana groups are referred as HH (dengue haemorrhagic fever cases), HF (dengue fever cases), HA (individuals with asymptomatic infection) and HC (controls). Similarly, GH, GF, GA and GC codes were used for the Guantanamo groups.

### Ethics statement

The study was conducted according to the Helsinki Declaration as a statement of ethical principles for medical research involving human subjects [46] and was approved by the Institutional Ethical Review Committee of the Institute of Tropical Medicine Pedro Kourí (IPK) and by the Ethical Committee of the Cuban National Academy of Sciences. Written informed consent was obtained from all individuals.

### GWAS genotyping and data quality control

Genotyping was performed for the Illumina Human Omni 2·5 chip and calls were obtained on the Illumina IScan Microarray System by using the Genome Studio software. Quality control was performed in PLINK [47], and SNPs with more than 5% missing genotypes, minor allele frequency (MAF) below 1%, and Hardy-Weinberg equilibrium deviation p-values of less than 0.001 were filtered out. All samples were checked for missingness (threshold of 1·93%), outliers in principal-component analysis (PCA), excess rate of heterozygous *loci*, and evidence of second-degree relatedness or higher (identity by descent >30%; or identity by state >90%). All studied samples passed these criteria. SNPs located in X and Y chromosomes and in mitochondrial DNA were removed, leading to a final account of 1,922,396 autosomal SNPs.

### Global ancestry in Cuba and its influence in dengue infection outcome

Cuban samples were merged with populations from Europe, Africa, Latin America and East Asia (Table A in S1 Text), so that the total final dataset contained 389,574 common SNPs. Ancestry components (*K*, from 2 to 10) were evaluated in all Cuban groups using the program ADMIXTURE [48], after pruning SNPs for pairwise linkage disequilibrium (r^2^ > 0·4 in 50-SNP windows), ending up with a total of 77,782 SNPs. The optimal *K* was estimated through cross-validation of the logistic regression. Two-tailed Wilcoxon rank-sum tests were applied to assess the significance between ancestry proportions in the Cuban groups.

### Fine-matched population structure correction and association analysis

Global admixture information was used for fine-matched correction for population structure. Comparison groups were organized by matching paired individuals who did not differ by more than 2% in the African ancestry. Havana and Guantanamo individuals were included together, but as far as possible the comparison pair was from the same city, and asymptomatic infected individuals were considered first than controls as individuals with no dengue disease symptoms. The groups consisted in: 54 asymptomatic/control versus DHF pairs (haemorrhagic comparison group—HCG); 74 asymptomatic/control versus DF pairs (fever comparison group—FCG); 111 asymptomatic/control versus DHF and DF pairs (overall comparison group–OCG). Single-locus allelic association analyses were carried out in PLINK [47] through the χ^2^ statistics. P-values were displayed as–log_10_ in Manhattan plots, obtained in HaploView.

### Admixture mapping

Local ancestry analysis was performed through RFMix algorithm [49]. 50 Southern European (Italian) and 50 Yoruban samples from the 1000 Genomes database [50] represented the parental European and African components, respectively. These samples were phased together with the Cuban samples in SHAPEIT [51] with the fine scale HapMap phase II genetic map. A test in chromosome 22 was performed with the Spanish sample from 1000 Genomes database replacing the Italian to confirm that results were identical. The difference in the African proportion between asymptomatic/controls and cases was calculated for every position along each chromosome in the Cuban comparison groups (HCG, FCG and OCG), and genomic regions significantly enriched for the African ancestry in asymptomatic/controls were considered as being outside the range defined by the genome mean + 3SD.

### Gene expression analysis in patients with dengue illness

A group of 20 patients with suspected dengue acute illness (clinical diagnosis was made by an experienced physician in dengue illness) were recruited at the Salvador Allende Hospital, Havana in 2014. Dengue infection was confirmed as described above. General signs and symptoms as well as warning signs (clinical fluid accumulation, mucosal bleeding, restlessness, severe abdominal pain, persistent vomiting as well as thrombocytopenia<100 platelets x 10^9^/L, and 20% or higher increase of haemo-concentration indicated by haematocrit or HTC) were recorded daily from recruitment to discharge. Three serial peripheral mononuclear cell (PBMC) samples were collected during hospitalization, at days 3, 7 and 30, after symptoms onset.

DNase-treated total RNA was isolated from PBMC using the RNeasy Mini kit (Qiagen, Hilden, Germany) and evaluated by using the Agilent 2100 Bioanalyzer (Agilent Technologies, Palo Alto, CA, USA). Expression levels of *OSBPL10*, *RXRA* and β-actin (housekeeping) genes were determined by RT-PCR (LightCycler 2.0 instrument, Roche), using LightCycler RNA Master SYBR Green I kit (Roche) and designed primers (Table B in S1 Text). Each sample was duplicated and the mean Ct values were normalized to an average Ct value of the housekeeping gene. The assay specificity was evaluated by melting curve analysis. Statistical analysis was performed using the non-parametric Wilcoxon-Mann-Whitney U mean rank test for quantitative variables.

### mRNA expression in other datasets, potential functional role and positive selection

Expression of significant genes and genotypes was checked also in public datasets: 1) 465 RNASeq performed in European and African lymphoblastoid cell lines by the 1000 Genomes project [21], expressed in reads per kilobase of exon per million reads mapped (RPKM) and extracted from ArrayExpress (E-GEUV-1); 2) Thai whole blood transcriptome (GDS5093) [22] from nine healthy controls, 28 samples collected between days 2 and 9 after onset of symptoms (acute illness) from secondarily infected patients (18 DF and 10 DHF), and 19 samples collected at convalescence, four weeks or later after discharge. GSEA software [52] was used for gene set enrichment analysis of the LXR/RXR activation pathway in the Thai dengue dataset [22] by considering three sets of genes: lipid metabolism, LXR/RXR activation and NF-kB activation.

Haplotter database (http://haplotter.uchicago.edu/) was used to explore recent positive selection through iHS measure (detects selective sweeps at 50–80% frequencies). By using the selscan package [53], another measure of positive selection (XP-EHH—detects selective sweeps above 80% frequency) was applied to the Cuban HCG.

The significant SNPs were ascertained for location in regulatory regions by using EPDNew [54] and HaploReg [55] tools. EDPNew comprises organism-specific (we obviously used *H*. *sapiens*) transcription start site (TSS) collections automatically assembled from carefully selected mass sequences for genome annotation data. HaploReg annotates non-coding diversity by using LD information from the 1000 Genomes Project that allows one to visualize linked SNPs and small indels along with their predicted chromatin state, conservation across mammals and their effect on regulatory motifs.

### Cell culture, construction of plasmids and generation of *OSBPL10* stable knockdown and mock cell lines

Two *OSBPL10* short hairpin RNAs (shRNA; TRCN0000147511 and TRCN0000149806 from Sigma Aldrich, St. Louis, MO USA) were cloned in the mammalian expression vector pcDNA3. These shRNAs were previously reported to induce 80–90% reduction of the OSBPL10 protein [56]. The plasmids and the control/mock plasmid were transformed into *E*. *coli* competent cells and the positive recombinant colonies were selected and amplified. The extracted recombinant plasmids were digested, subjected to DNA sequencing and purified by QIAprep Spin Miniprep Kit (Qiagen, Hilden, Germany), following the recommended procedure.

THP-1 cells obtained from American Type Culture Collection (ATCC, Rockville, MD USA) were cultured in complete grow medium RPMI1640 supplemented with 10% fetal bovine serum (FBS), L-glutamine and 0.1 mM MEM non-essential amino acids solution (Thermo Fisher Scientific, Waltham, MA USA) at 37°C with 5%CO_2_. Transfection of the two shRNA and mock plasmids into THP-1 cells was done by using Lipofectamine LTX Reagent (Thermo Fisher Scientific, Waltham, MA USA) and the recombinant cells (THP1sh1/OSBPL10, THP1sh2/OSBPL10, THP1/mock) were selected for Neomicin resistance.

### Dengue virus preparation and infection of THP-1 cells

C6/36 cell lines from *A*. *albopictus* were grown to confluence, infected at a multiplicity of infection of 0.1 particle forming units (PFU) per cell with the dengue strain DENV-2 A15 Cuba 1981 and cultured in minimum essential medium (MEM) supplemented with 2% fetal calf serum. When a cytopathic effect higher than 50% was noted, culture supernatant was clarified by centrifugation at 10,000 rpm for 30 min at 4 °C. BHK21, clone 15 cell line was used for virus titration as previously described [57]. DENV-2 A15 Cuba titer was of 2x10^6^ PFU/ml. Absence of lipopolysaccharide contamination in the viral preparations was confirmed by the Limulus Amebocyte Lysate test (Bio- Whittaker Inc., Walkersville, MD USA).

THP-1 cells were infected with dengue virus at a multiplicity of infection (MOI) of 0.1. The virus inoculum at a 70–90% confluence was incubated in serum-free medium at 37°C for 1h. The unadsorbed viruses were removed by washing the cells three times with plain medium. The dengue-virus-infected and non-infected cells were replenished with fresh complete medium and incubated for another 72 hours.

### *OSBPL10* mRNA expression analysis and dengue viral load measurement by qRT-PCR

DNase-treated total RNA was isolated from all THP-1 cell variants by means of RNeasy Mini kit (Qiagen, Hilden, Germany) and evaluated by using the Agilent 2100 Bioanalyzer (Agilent Technologies, Palo Alto, CA, USA). The cDNA was synthesized from mRNA with poly(dT) primers and Superscript II reverse transcriptase (Life Technologies, Rockville, MD, USA) and quantified by real-time PCR analysis using the ABI Prism 7700 sequence detection system (Applied Biosystems, Foster City, CA, USA). Samples were analyzed in triplicates for the expression of *OSBPL10*, as well as for the housekeeping gene β-actin. And each experience was repeated three times (nine values for each condition). Specific expression was calculated in relation to that of β-actin, by using the delta/delta Ct method as recommended by ABI.

RNA from 140 μl of *OSBPL10* silenced cell line control THP-1 supernatant or cell culture lysate was extracted, using the QIAamp viral RNA extraction kit (Qiagen, Hilden, Germany) and amplified with TaqMan Assay. Standard curves were obtained with titrated DENV-2 supernatants serially diluted from 10^6^ to 10 PFU/mL. The standard curve obtained by serial dilution of titrated DENV-2 supernatants had a -3.4 slope, the amplification efficiency was of 92.2% and the detection limit was estimated at 10 PFU equivalents/mL. We performed quantitative RT-PCR analyses of viral mRNA isolated from cellular extracts (intracellular) and culture supernatants (extracellular). Intracellular RNA levels indicate viral replication, whereas extracellular viral RNA is a measure of released viral particles in the culture supernatant.

## Supporting information

S1 Text Supporting information containing 27 figures and 19 tables. (DOCX)
