# Supplementary material for: OSBPL10, RXRA and lipid metabolism confer African-ancestry protection against dengue haemorrhagic fever in admixed Cubans
Source: PLoS Pathog. 2017 Feb 27;13(2):e1006220. doi: 10.1371/journal.ppat.1006220 (PMC5344536; doi:10.1371/journal.ppat.1006220)
Supplement: S1 Text — (DOCX) [file ppat.1006220.s001.docx]

***OSBPL10*, *RXRA* and Lipid Metabolism Confer African-Ancestry Protection Against Dengue Haemorrhagic Fever in Admixed Cubans**

# Beatriz Sierra, Petr Triska, Pedro Soares, Gissel Garcia, Ana B. Perez, Eglys Aguirre, Marisa Oliveira, Bruno Cavadas, Béatrice Regnault, Mayling Alvarez, Didye Ruiz, David C. Samuels, Anavaj Sakuntabhai, Luisa Pereira, Maria G. Guzman

1.1- Additional material and methods

1.2- Global ancestry analysis in detail

1.3- Iterative model of global ancestry influence in dengue in Cuba

1.4- Association test

1.5- Admixture mapping

1.6- P-values of specific regions

1.7- XP-EHH selection test

1.8- In depth investigation of *OSBPL10* and *RXRA* genes

1.9- GSEA analysis

1.10- References

*1.1- Additional material and methods*

In order to ascertain the global ancestry background, the Cuban samples were compared with populations from Europe, Africa, Latin America and East Asia, from the 1000 Genomes project [[1](#_ENREF_1)] and another worldwide dataset [[2](#_ENREF_2)] (Table A)

**Table A. Populations used in the global ancestry evaluation.**

| Population group | Population | Code | Sample size | Reference |
| --- | --- | --- | --- | --- |
| America | Havana controls | HC | 47 | This study |
|  | Havana asymptomatic | HA | 32 | This study |
|  | Havana haemorrhagic | HH | 31 | This study |
|  | Havana fever | HF | 36 | This study |
|  | Guantanamo controls | GC | 42 | This study |
|  | Guantanamo asymptomatic | GA | 16 | This study |
|  | Guantanamo haemorrhagic | GH | 29 | This study |
|  | Guantanamo fever | GF | 41 | This study |
|  | Barbados |  | 50 | [[1](#_ENREF_1)] |
|  | African Americans |  | 50 | [[1](#_ENREF_1)] |
|  | Peruvians (Lima) |  | 50 | [[1](#_ENREF_1)] |
|  | Mexicans (Los Angeles) |  | 50 | [[1](#_ENREF_1)] |
|  | Colombians (Medellin) |  | 50 | [[1](#_ENREF_1)] |
| Sub-Saharan Africa | Mandenka (Senegal) |  | 20 | [[2](#_ENREF_2)] |
|  | Gambian (Western Divisions) |  | 50 | [[1](#_ENREF_1)] |
|  | Mende (Sierra Leone) |  | 50 | [[1](#_ENREF_1)] |
|  | Esan (Nigeria) |  | 50 | [[1](#_ENREF_1)] |
|  | Yoruba (Nigeria) |  | 50 | [[1](#_ENREF_1)] |
|  | Luhya (Kenya) |  | 50 | [[1](#_ENREF_1)] |
|  | Bantus (South Africa) |  | 19 | [[2](#_ENREF_2)] |
| North Africa | Mozabite (Algeria) |  | 30 | [[2](#_ENREF_2)] |
| Near East | Druze (Israel) |  | 47 | [[2](#_ENREF_2)] |
|  | Palestinian (Israel) |  | 51 | [[2](#_ENREF_2)] |
|  | Bedouin (Israel) |  | 48 | [[2](#_ENREF_2)] |
| Europe | French Basque |  | 24 | [[2](#_ENREF_2)] |
|  | Sardinian |  | 28 | [[2](#_ENREF_2)] |
|  | Italians (Tuscany) |  | 50 | [[1](#_ENREF_1)] |
|  | Iberians (Spain) |  | 50 | [[1](#_ENREF_1)] |
|  | United Kingdom |  | 50 | [[1](#_ENREF_1)] |
| Asia | Han Chinese (Beijing) |  | 50 | [[1](#_ENREF_1)] |

The primers used in the RT-PCR assays are displayed in Table B.

**Table B. Sequences of oligonucleotides used to perform the RT-PCRs.**

| Gene | Sequence | Reference |
| --- | --- | --- |
| RXRα | Forward: TTCGCTAAGCTCTTGCTC | [[3](#_ENREF_3)] |
|  | Reverse: ATAAGGAAGGTGTCAATGGG |  |
| β-Actin | Forward: CCCCAGGCACCAGGGCGTGAT | [[3](#_ENREF_3)] |
|  | Reverse: GTCATCTTCTCGCGGTTGGCCTTGGGGT |  |
| OSBPL10 | Forward: cat gct ggt agt gta ctc tgc t | [[4](#_ENREF_4)] |
|  | Reverse: cgg gag ctt gga gca ctc tt |  |
| β-Actin | Forward: cac act gtg ccc atc tac ga | [[4](#_ENREF_4)] |
|  | Reverse: gcc atc tct tgc tcg aag tc |  |

*1.2- Global ancestry analysis in detail*

Fig A reports the Admixture results from K=2 to K=9. As can be observed, first components to be separated are African and non-African, and then, the European from the Asian. K=4 already allows to distinguish between East Asian and Amerindian. K=5 separates a Near Eastern/North African/Arabian component from the European one, and K=6 separates between Western African and Western/Central African components. K=7 separates Near Eastern/North African from Arabian, K=8 isolates the Near Eastern from North African, and K=9 identifies the Eastern/Southern African component. As can be seen in Fig B, which reports the results for the error cross-validation, the models from K=5 to K=9 have similarly low errors.


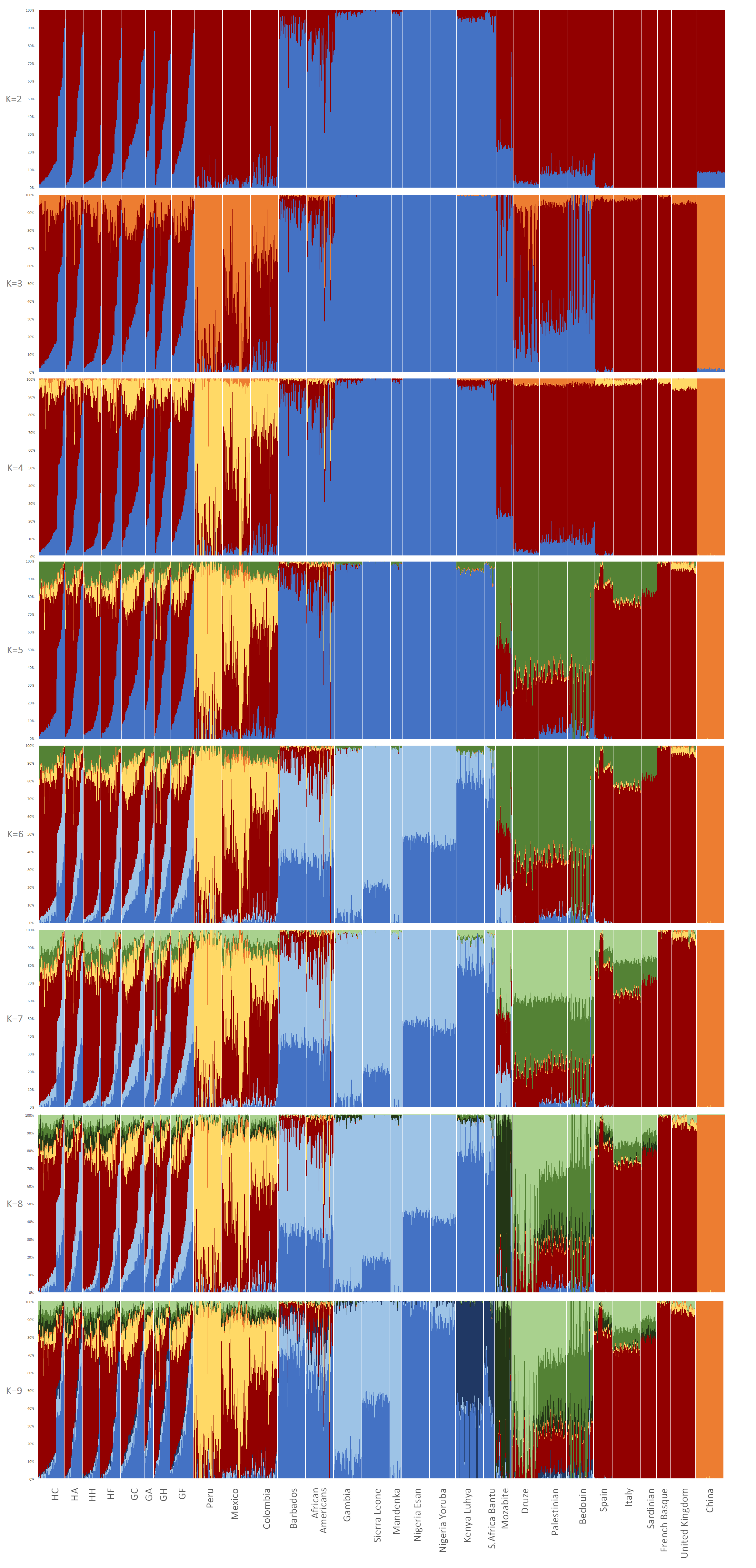


**Fig A. ADMIXTURE results for K between 2 and 9.**

**Fig B. Cross-validation error of diverse K in ADMIXTURE analysis.**

As we have already referred in the main text, African and European ancestries are negatively correlated (Fig C) and their frequencies are statiscally different between Havana and Guantanamo, the same being true for the Native American component (6.5% in Havana and 13.5% in Guantanamo; p=1x10^-6^), while the East Asian component is equally vestigial in both (1.9% and 0.7% respectively; p=0.224).

**Fig C. Correlation between European and African proportions in Cuban individuals analysed here.**

Comparing the Cuban admixture results with other Latin American populations, the most significant difference is the lower proportion of Native American background in Cubans than in others: 80% in Peru, 51% in Mexico and 28% in Colombia. This is in accordance with the dramatic reduction of the native population in Cuba after the European settlement, with population records of only 3.4% of natives by 1555. In contrast, the Caribbean population of Barbados has a dominant African ancestry, even higher than the African-American sample, 88% and 76% respectively, mixed essentially with a European background.

We also checked the sub-structure within the African and European components between the two cities. At K=9 ADMIXTURE (Fig A), there are three African sub-components: a main Western African background (light blue) present in Gambia and Mandenka samples; a Western/Central African one (intermediate blue) which dominates the Nigerian sample; while the Kenyan Luhya and the South African Bantu have 60-50% of an Eastern/Southern African component (dark blue). In Cuba, the relative proportions from the diverse African regions to the African component in Havana and Guantanamo are the same (Fig D): 20-24% of Western African; 66%-70% of Western/Central African and 10% of Eastern/Southern African. These relative African contributions in Cuba are identical to the ones observed in Barbados and African Americans, despite the high differences in the global African proportions between Cuba and these two samples. Within Europe, there is a main European component (dark red - dominant in United Kingdom and French Basque), but Spanish and Sardinians have Berber/North African (dark green), Near Eastern (light green) and Arabian (intermediate green) influences, similarly to the Italians. The Southern European influence in Cuba (Fig E) is well supported by the observation of similar relative contributions of the “pure” European, North African, Near Eastern and Arabian pools to the Spanish/Italian ones. We did not identify the particular Iberian component reported by Moreno-Estrada et al. [[5](#_ENREF_5)], which according to these authors would be low frequent in Spain and high frequent in the Americas, due to a strong founder effect. Historical records in Cuba testify that Spanish came between 15^th^ and 20^th^ centuries from all regions of the country (including Canarias, who have autochthonous Berber descendent lineages), that they established mainly in Havana and surroundings, and that this migrant component was further enriched in the 20^th^ century with other waves mainly from Spain, England, Italy, France, Russia and Portugal. It is easier to conciliate these historical records with results observed by us than the ones reported in Moreno-Estrada et al. [[5](#_ENREF_5)].

**Fig D. The relative proportions from the diverse African regions to the African component in Havana, Guantanamo, Barbados and African-Americans.**

**Fig E. The relative proportions from the “pure” European, North African, Near Eastern and Arabian backgrounds to the broad European component in Havana, Guantanamo, Latin American and European populations.**

*1.3- Iterative model of global ancestry influence in dengue in Cuba*

We used R to apply an iterative model to the global ancestry influence in dengue. As the African, European, Native American and East Asian components sum up necessarily to 100%, enforcing co-linearity between the variables, and the European and African components are highly negatively correlated (r^2^=0.9319; Fig C), we discarded the European component from the analysis, and East Asian ancestry was also ignored as it is negligible (<3%). Thus, the following variables were considered: African and Native American ancestries as discontinuous variables; Havana and Guantanamo locations as binary variables; and asymptomatic and DHF phenotypes as binary variables.

A significant p-value was observed for the protection conferred by African ancestry against DHF in Havana (p-value=0.002; Fig F), while the Native American protection in Havana was not statistically significant and no significant ancestry influence was observed in Guantanamo.


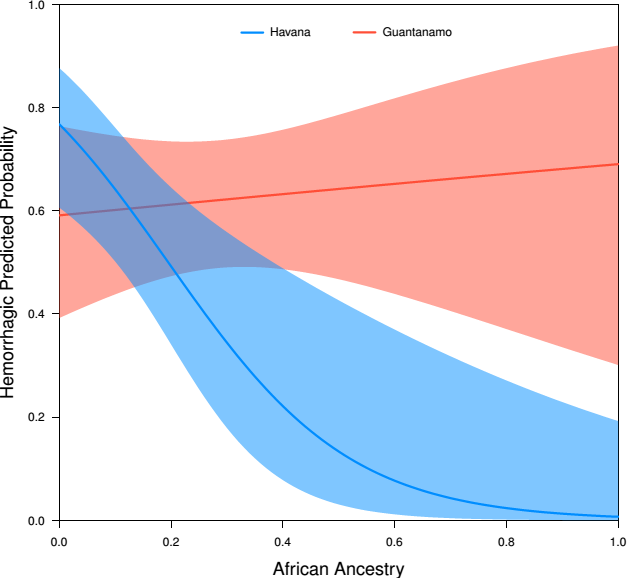


**Fig F. The DHF predicted probability curves in function of the African ancestry in Havana (blue) and Guantanamo (pink), by comparison with asymptomatic.**

*1.4- Association test*

The lowest p-values for the association tests applied to the fine-matched population corrected comparison groups (HCG, FCG and OCG) are reported in Tables C-E.

**Table C. Lowest p-values in the association test in all SNPs in the HCG.** NA – not assigned. MAF in the 108 individuals higher or equal to 5%.

| Chr | ID in the chip | ID in Ensembl | Position in GRCh37 | Gene | Allele | Frequency in hemorrhagic | Frequency in asymptomatic control | Other allele | χ2 | p-value | Odds ratio | Odds ratio alternative allele |
| --- | --- | --- | --- | --- | --- | --- | --- | --- | --- | --- | --- | --- |
| 2 | rs1564004 | rs1564004 | 18098857 | KCNS3 | A | 0.1111 | 0.3611 | G | 18.71 | 1.52e-05 | 0.2212 |  |
| 2 | kgp3066410 | rs13008425 | 18124348 | KCNS3 | G | 0.1296 | 0.4074 | T | 21.21 | 4.109e-06 | 0.2166 |  |
| 2 | kgp9463911 | NA | 18128149 | KCNS3 | C | 0.1275 | 0.4135 | T | 21.28 | 3.967e-06 | 0.2072 |  |
| 2 | rs10495673 | rs10495673 | 18141228 | KCNS3 | G | 0.1389 | 0.3796 | A | 16.3 | 5.417e-05 | 0.2636 |  |
| 2 | rs11903301 | rs11903301 | 18142133 | KCNS3 | T | 0.1667 | 0.4167 | C | 16.34 | 5.304e-05 | 0.28 |  |
| 2 | kgp3546000 | rs11894068 | 18143572 | KCNS3 | A | 0.1667 | 0.4074 | G | 15.29 | 9.206e-05 | 0.2909 |  |
| 2 | rs12692642 | rs12692642 | 162636614 | SLC4A10 | C | 0.5 | 0.2315 | T | 16.78 | 4.188e-05 | 3.32 | 0.3012 |
| 2 | kgp6131391 | rs1902055 | 165838846 |  | C | 0.01887 | 0.1792 | T | 15.27 | 9.294e-05 | 0.08806 |  |
| 2 | rs1993308 | rs1993308 | 165843619 |  | G | 0.05556 | 0.25 | C | 15.77 | 7.14e-05 | 0.1765 |  |
| 2 | rs17808106 | rs17808106 | 169366003 | CERS6 | T | 0.3519 | 0.1204 | C | 16.04 | 6.193e-05 | 3.967 | 0.2521 |
| 2 | rs13398999 | rs13398999 | 169386870 | CERS6 | C | 0.3241 | 0.1019 | T | 15.91 | 6.643e-05 | 4.228 | 0.2365 |
| 3 | kgp4222038 | rs4858062 | 23268897 | UBE2E2 | A | 0.6019 | 0.3333 | G | 15.64 | 7.663e-05 | 3.023 | 0.3308 |
| 3 | kgp3657536 | rs4600849 | 32027672 | OSBPL10/ZNF860 | C | 0.3113 | 0.6111 | T | 19.34 | 1.094e-05 | 0.2877 |  |
| 3 | kgp3944551 | rs11129475 | 32030544 | OSBPL10/ZNF860 | T | 0.2037 | 0.5463 | C | 27.04 | 1.991e-07 | 0.2125 |  |
| 3 | rs6419811 | rs6419811 | 32031135 | OSBPL10/ZNF860 | G | 0.2685 | 0.5833 | A | 21.89 | 2.891e-06 | 0.2622 |  |
| 3 | rs11718700 | rs11718700 | 32033248 | OSBPL10 | C | 0.2685 | 0.5741 | A | 20.68 | 5.431e-06 | 0.2724 |  |
| 3 | kgp2595741 | rs975406 | 32035587 | OSBPL10 | C | 0.2685 | 0.5741 | T | 20.68 | 5.431e-06 | 0.2724 |  |
| 3 | kgp8630333 | rs7639637 | 32036042 | OSBPL10 | C | 0.2685 | 0.5741 | A | 20.68 | 5.431e-06 | 0.2724 |  |
| 3 | kgp5843296 | rs6764354 | 126254692 | CHST13 | A | 0.1759 | 0.4352 | G | 17.11 | 3.536e-05 | 0.2771 |  |
| 3 | rs6781798 | rs6781798 | 126260163 | CHST13 | T | 0.1667 | 0.4259 | C | 17.41 | 3.016e-05 | 0.2696 |  |
| 3 | rs2981013 | rs2981013 | 128188343 | DNAJB8/DNAJB8-AS1 | G | 0.3796 | 0.1389 | A | 16.3 | 5.417e-05 | 3.794 | 0.2636 |
| 4 | rs884049 | rs884049 | 109805696 | COL25A1 | G | 0.1204 | 0.3611 | A | 17.12 | 3.505e-05 | 0.2421 |  |
| 4 | kgp8873108 | rs11098007 | 109811779 | COL25A1 | T | 0.1389 | 0.3704 | C | 15.25 | 9.44e-05 | 0.2742 |  |
| 5 | rs17138918 | rs17138918 | 115469668 | COMMD10 | C | 0.1667 | 0.009259 | T | 16.68 | 4.43e-05 | 21.4 | 0.0467 |
| 5 | kgp726185 | rs26584 | 115766776 |  | C | 0.09259 | 0.3241 | A | 17.54 | 2.808e-05 | 0.2128 |  |
| 6 | kgp9699004 | rs2181943 | 168810621 |  | C | 0.3611 | 0.6389 | T | 16.67 | 4.456e-05 | 0.3195 |  |
| 6 | kgp5788705 | rs2147457 | 168810725 |  | A | 0.6204 | 0.3426 | G | 16.69 | 4.402e-05 | 3.136 | 0.3189 |
| 6 | rs2763264 | rs2763264 | 168814455 |  | T | 0.6204 | 0.3426 | G | 16.69 | 4.402e-05 | 3.136 | 0.3189 |
| 7 | kgp12446015 | rs2390366 | 20842465 |  | T | 0.6038 | 0.3056 | C | 19.2 | 1.178e-05 | 3.463 | 0.2888 |
| 7 | rs2108520 | rs2108520 | 20844094 |  | A | 0.5833 | 0.3056 | G | 16.88 | 3.992e-05 | 3.182 | 0.3143 |
| 7 | kgp7307095 | rs7808965 | 20848979 |  | T | 0.6204 | 0.3519 | C | 15.59 | 7.883e-05 | 3.01 | 0.3322 |
| 9 | kgp11827519 | rs1663112 | 80215964 | GNA14 | G | 0.3426 | 0.6204 | T | 16.69 | 4.402e-05 | 0.3189 |  |
| 9 | rs1953330 | rs1953330 | 80221466 | GNA14 | G | 0.2315 | 0.5 | T | 16.78 | 4.188e-05 | 0.3012 |  |
| 9 | kgp3867710 | rs11140094 | 86064897 | FRMD3 | A | 0.08333 | 0.3302 | T | 19.96 | 7.914e-06 | 0.1844 |  |
| 9 | rs4877767 | rs4877767 | 86069237 | FRMD3 | T | 0.1296 | 0.3611 | C | 15.63 | 7.715e-05 | 0.2635 |  |
| 10 | rs1556408 | rs1556408 | 12840842 | CAMK1D | T | 0.5648 | 0.2778 | C | 18.25 | 1.939e-05 | 3.374 | 0.2964 |
| 10 | kgp6646411 | rs12768610 | 12842026 | CAMK1D | G | 0.5392 | 0.2685 | A | 16.02 | 6.28e-05 | 3.188 | 0.3137 |
| 10 | rs2493765 | rs2493765 | 12842920 | CAMK1D | A | 0.3426 | 0.6111 | G | 15.61 | 7.794e-05 | 0.3316 |  |
| 10 | kgp2063598 | rs17391197 | 98282160 | TM9SF3 | G | 0.01852 | 0.1759 | A | 15.24 | 9.448e-05 | 0.08838 |  |
| 10 | kgp4005656 | rs79353623 | 98289951 | TM9SF3 | C | 0.01852 | 0.1852 | T | 16.4 | 5.136e-05 | 0.08302 |  |
| 10 | rs10736113 | rs10736113 | 98312891 | TM9SF3 | C | 0.01852 | 0.1852 | T | 16.4 | 5.136e-05 | 0.08302 |  |
| 10 | kgp11284179 | rs7923910 | 98348430 | TM9SF3-PIK3AP1 | T | 0.01852 | 0.1759 | C | 15.24 | 9.448e-05 | 0.08838 |  |
| 10 | kgp8009179 | rs11592969 | 98354812 | PIK3AP1 | G | 0.01852 | 0.1759 | A | 15.24 | 9.448e-05 | 0.08838 |  |
| 10 | rs10786304 | rs10786304 | 98366585 | PIK3AP1 | A | 0.01852 | 0.1944 | G | 17.57 | 2.775e-05 | 0.07817 |  |
| 10 | kgp2633248 | rs72821772 | 98942314 | SLIT1-ARHGAP19 | A | 0 | 0.1635 | G | 17.49 | 2.886e-05 | 0 |  |
| 10 | kgp2875044 | rs200549826 | 113668975 |  | A | 0.3056 | 0.5741 | C | 15.8 | 7.029e-05 | 0.3265 |  |
| 10 | rs1421055 | rs1421055 | 113696815 |  | G | 0.2925 | 0.5566 | A | 15.14 | 9.997e-05 | 0.3293 |  |
| 10 | kgp22050550 | NA | 113734088 |  | G | 0.3019 | 0.5741 | A | 16.09 | 6.039e-05 | 0.3208 |  |
| 11 | kgp9104118 | rs58552415 | 94785503 |  | A | 0.0463 | 0.2407 | C | 16.61 | 4.592e-05 | 0.1531 |  |
| 11 | kgp7184307 | rs2895587 | 94793923 |  | A | 0.0463 | 0.2315 | G | 15.48 | 8.321e-05 | 0.1612 |  |
| 11 | kgp11939959 | rs1374666 | 94811734 |  | G | 0.06481 | 0.2642 | A | 15.54 | 8.086e-05 | 0.1931 |  |
| 11 | rs12287409 | rs12287409 | 132843990 | OPCML | G | 0.009259 | 0.1574 | T | 15.52 | 8.185e-05 | 0.05003 |  |
| 11 | kgp4100203 | rs78575779 | 132853598 | OPCML | G | 0.009259 | 0.1604 | A | 15.86 | 6.828e-05 | 0.04893 |  |
| 12 | rs7314836 | rs7314836 | 88664574 |  | G | 0.3611 | 0.1296 | T | 15.63 | 7.715e-05 | 3.795 | 0.2635 |
| 12 | rs11104784 | rs11104784 | 88670634 |  | T | 0.3611 | 0.1296 | C | 15.63 | 7.715e-05 | 3.795 | 0.2635 |
| 12 | rs7966318 | rs7966318 | 88677324 |  | A | 0.3611 | 0.1296 | C | 15.63 | 7.715e-05 | 3.795 | 0.2635 |
| 12 | kgp6194241 | NA | 112244144 | ALDH2 | A | 0.06481 | 0.2642 | C | 15.54 | 8.086e-05 | 0.1931 |  |
| 12 | rs3177647 | rs3177647 | 112277576 |  | A | 0.07407 | 0.2778 | G | 15.46 | 8.445e-05 | 0.208 |  |
| 12 | kgp6174673 | rs7970847 | 112281493 | MAPKAPK5 | C | 0.07407 | 0.2778 | T | 15.46 | 8.445e-05 | 0.208 |  |
| 12 | kgp11702452 | rs7296199 | 112291844 | MAPKAPK5 | C | 0.07407 | 0.2778 | T | 15.46 | 8.445e-05 | 0.208 |  |
| 14 | rs2749486 | rs2749486 | 40166245 |  | A | 0.3148 | 0.5833 | G | 15.74 | 7.277e-05 | 0.3282 |  |
| 14 | rs2781339 | rs2781339 | 40173091 |  | T | 0.3148 | 0.5833 | C | 15.74 | 7.277e-05 | 0.3282 |  |
| 14 | kgp8437518 | rs1958978 | 40238708 |  | C | 0.5463 | 0.2642 | A | 17.65 | 2.654e-05 | 3.354 | 0.2982 |
| 18 | kgp4544975 | rs55940802 | 49173811 |  | T | 0.2407 | 0.0463 | C | 16.61 | 4.592e-05 | 6.532 | 0.1531 |
| 18 | rs9961909 | rs9961909 | 49180597 |  | C | 0.2407 | 0.0463 | T | 16.61 | 4.592e-05 | 6.532 | 0.1531 |
| 19 | rs440597 | rs440597 | 22096519 |  | C | 0.2407 | 0.5 | T | 15.56 | 7.973e-05 | 0.3171 |  |
| 19 | kgp1244775 | rs4932746 | 22269847 | ZNF257 | T | 0.2778 | 0.07407 | C | 15.46 | 8.445e-05 | 4.808 | 0.2080 |
| 19 | kgp1866342 | rs12976555 | 22434811 |  | T | 0.2315 | 0.0463 | G | 15.48 | 8.321e-05 | 6.205 | 0.1612 |
| 19 | kgp9413209 | rs11668533 | 52052004 |  | A | 0.463 | 0.1852 | G | 19.02 | 1.293e-05 | 3.793 | 0.2636 |
| 19 | kgp8018712 | rs4802833 | 52147594 | SIGLEC14 | G | 0.1667 | 0.4118 | T | 15.44 | 8.511e-05 | 0.2857 |  |
| 20 | rs6080201 | rs6080201 | 16264511 | KIF16B | A | 0.1667 | 0.4167 | G | 16.34 | 5.304e-05 | 0.28 |  |
| 20 | kgp4148242 | rs6080207 | 16270970 | KIF16B | T | 0.1667 | 0.4167 | C | 16.34 | 5.304e-05 | 0.28 |  |
| 20 | rs6080212 | rs6080212 | 16277630 | KIF16B | A | 0.0463 | 0.2315 | G | 15.48 | 8.321e-05 | 0.1612 |  |
| 21 | rs2836344 | rs2836344 | 39737626 |  | C | 0.4327 | 0.1481 | T | 20.93 | 4.762e-06 | 4.386 | 0.2280 |
| 21 | kgp1198154 | rs2836346 | 39739665 | ERG | T | 0.1204 | 0.3491 | A | 15.62 | 7.724e-05 | 0.2552 |  |
| 21 | rs730853 | rs730853 | 39749646 | ERG | A | 0.3796 | 0.1296 | G | 17.78 | 2.477e-05 | 4.109 | 0.2434 |
| 21 | rs6517463 | rs6517463 | 39752673 | ERG | T | 0.3889 | 0.1389 | C | 17.37 | 3.069e-05 | 3.945 | 0.2535 |
| 22 | kgp57547 | rs56234606 | 27463700 |  | T | 0.0463 | 0.2315 | C | 15.48 | 8.321e-05 | 0.1612 |  |
| 22 | kgp746049 | rs55745232 | 27468857 |  | C | 0.0463 | 0.2407 | T | 16.61 | 4.592e-05 | 0.1531 |  |

**Table D. Lowest p-values in the association test in all SNPs in the FCG.** NA – not assigned. MAF in the 148 individuals higher or equal to 5%.

| Chr | ID in chip | ID in Ensembl | Position in GRCh37 | Gene | Allele | Frequency in fever | Frequency in asymptomatic control | Other allele | p-value | Odds ratio | Odds ratio alternative allele |
| --- | --- | --- | --- | --- | --- | --- | --- | --- | --- | --- | --- |
| 1 | kgp4145901 | rs11807645 | 4854949 | AJAP1 | A | 0.277 | 0.5405 | G | 4.01E-06 | 0.3257 |  |
| 1 | rs7544257 |  | 5732250 |  | G | 0.223 | 0.06164 | A | 7.74E-05 | 4.368 | 0.2289 |
| 1 | kgp4505000 | rs9724874 | 5805819 |  | C | 0.4122 | 0.1781 | T | 1.10E-05 | 3.236 | 0.3090 |
| 1 | rs2361030 |  | 13906554 | PDPN | A | 0.3784 | 0.1757 | G | 9.77E-05 | 2.856 | 0.3501 |
| 1 | rs9430069 |  | 211726975 |  | C | 0.1622 | 0.3649 | T | 7.55E-05 | 0.3369 |  |
| 2 | kgp6135765 | rs79276301 | 37048153 |  | C | 0.02027 | 0.1486 | T | 7.15E-05 | 0.1185 |  |
| 2 | kgp1374081 | rs2565633 | 38079392 |  | T | 0.3311 | 0.5608 | C | 7.02E-05 | 0.3876 |  |
| 2 | kgp1637200 | rs35548551 | 72039714 |  | T | 0.3784 | 0.1689 | G | 5.31E-05 | 2.995 | 0.3339 |
| 2 | kgp5205739 | rs2176717,  rs373780229 | 136899859 |  | C | 0.4189 | 0.1959 | T | 3.23E-05 | 2.958 | 0.3381 |
| 2 | rs1519523 |  | 136934449 |  | T | 0.4054 | 0.1824 | G | 2.55E-05 | 3.056 | 0.3272 |
| 2 | rs882300 |  | 136976255 |  | A | 0.4122 | 0.1824 | G | 1.54E-05 | 3.142 | 0.3183 |
| 2 | kgp12177071 | rs13017796 | 165454112 | GRB14 | T | 0.05405 | 0.2095 | G | 7.73E-05 | 0.2157 |  |
| 2 | rs777715 |  | 195955184 |  | T | 0.3176 | 0.1149 | C | 2.28E-05 | 3.586 | 0.2789 |
| 2 | kgp595863 | rs17790821 | 218733667 | TNS1 | A | 0 | 0.1081 | G | 3.91E-05 | 0 |  |
| 2 | rs16866294 |  | 225759978 | DOCK10 | T | 0.08108 | 0.25 | C | 9.24E-05 | 0.2647 |  |
| 2 | kgp3444795 | rs283478 | 233649377 | GIGYF2 | A | 0.1338 | 0.3592 | G | 1.05E-05 | 0.2756 |  |
| 3 | rs4685867 |  | 5134494 |  | A | 0.3649 | 0.6081 | C | 2.83E-05 | 0.3702 |  |
| 3 | kgp12265183 | rs17201070 | 18117384 | TBC1D5 | G | 0.06849 | 0.2635 | A | 7.25E-06 | 0.2055 |  |
| 3 | kgp2412052 | rs4688910 | 18140116 | TBC1D5 | G | 0.09589 | 0.277 | A | 6.83E-05 | 0.2768 |  |
| 3 | rs4688908 |  | 18152950 | TBC1D5 | G | 0.2986 | 0.5274 | A | 7.65E-05 | 0.3815 |  |
| 3 | rs1876168 |  | 31039428 |  | G | 0.3716 | 0.6014 | A | 7.69E-05 | 0.3921 |  |
| 3 | rs1567025 |  | 31042458 |  | C | 0.3378 | 0.5743 | T | 4.42E-05 | 0.3782 |  |
| 3 | kgp117611 | rs7642265 | 31049205 |  | G | 0.3311 | 0.5676 | T | 4.32E-05 | 0.3771 |  |
| 3 | rs7647494 |  | 36573091 | STAC | A | 0.04054 | 0.1892 | C | 6.06E-05 | 0.1811 |  |
| 3 | kgp2102777 | rs76876765 | 36582555 | STAC | T | 0.04054 | 0.1892 | C | 6.06E-05 | 0.1811 |  |
| 3 | rs11130000 |  | 43387934 | SNRK | C | 0.3243 | 0.1284 | A | 5.63E-05 | 3.259 | 0.3068 |
| 3 | kgp12486580 | rs9815800 | 67876667 |  | T | 0.08333 | 0.2671 | C | 3.95E-05 | 0.2494 |  |
| 3 | kgp1886552 | rs1108584 | 129320301 | PLXND1 | G | 0.2808 | 0.09459 | A | 4.24E-05 | 3.737 | 0.2676 |
| 3 | kgp5410233 | rs73873389 | 156181246 | KCNAB1 | T | 0.02055 | 0.1622 | C | 2.62E-05 | 0.1084 |  |
| 3 | rs13315136 |  | 156182208 | KCNAB1 | C | 0.09459 | 0.2838 | A | 3.25E-05 | 0.2637 |  |
| 3 | kgp1058828 | rs41267915 | 156183382 | KCNAB1 | G | 0.06081 | 0.226 | A | 5.17E-05 | 0.2217 |  |
| 3 | kgp4923452 | rs13095723 | 156211203 | KCNAB1 | G | 0.03472 | 0.1757 | T | 9.26E-05 | 0.1688 |  |
| 3 | kgp84775 | rs9880257 | 188643729 |  | C | 0.08108 | 0.25 | T | 9.24E-05 | 0.2647 |  |
| 4 | kgp3432145 | rs17768776 | 2306259 | ZFYVE28 | T | 0.0473 | 0.1959 | C | 9.14E-05 | 0.2037 |  |
| 4 | kgp5886524 | rs4689736 | 7413267 | SORCS2 | A | 0.1959 | 0.4054 | T | 8.52E-05 | 0.3574 |  |
| 4 | kgp9191017 | rs6840085 | 8155373 | ABLIM2 | T | 0.1959 | 0.0473 | C | 9.14E-05 | 4.909 | 0.2037 |
| 4 | rs13102223 |  | 30991363 | PCDH7 | C | 0.06081 | 0.223 | T | 6.40E-05 | 0.2256 |  |
| 4 | kgp663875 | rs4336208 | 152332114 | FAM160A1 | G | 0.1622 | 0.02703 | A | 7.12E-05 | 6.968 | 0.1435 |
| 4 | kgp968528 | rs28479832 | 152332872 | FAM160A1 | T | 0.1622 | 0.02703 | G | 7.12E-05 | 6.968 | 0.1435 |
| 4 | kgp985368 | rs28378042 | 152379794 | FAM160A1 | C | 0.1622 | 0.02703 | T | 7.12E-05 | 6.968 | 0.1435 |
| 4 | kgp3012633 | rs28404083 | 189138387 |  | A | 0.06757 | 0.2297 | C | 8.81E-05 | 0.243 |  |
| 4 | kgp6159572 | rs10033063 | 189139054 |  | G | 0.07432 | 0.2432 | A | 7.01E-05 | 0.2498 |  |
| 5 | rs1078375 |  | 40977760 | C7 | T | 0.5405 | 0.2635 | C | 1.17E-06 | 3.288 | 0.3041 |
| 5 | kgp5765195 | rs28489073 | 81626372 | ATP6AP1L | C | 0.4459 | 0.223 | T | 4.79E-05 | 2.805 | 0.3565 |
| 5 | rs6867982 |  | 81630324 | ATP6AP1L | T | 0.4459 | 0.223 | C | 4.79E-05 | 2.805 | 0.3565 |
| 5 | kgp7083636 | rs2436389 | 139416265 | NRG2 | C | 0.1689 | 0.3819 | A | 4.49E-05 | 0.3289 |  |
| 5 | kgp7686575 | rs10058307 | 142037091 | FGF1 | A | 0.04054 | 0.1849 | G | 8.80E-05 | 0.1862 |  |
| 5 | rs2964243 |  | 151921873 |  | T | 0.5608 | 0.3311 | C | 7.02E-05 | 2.58 | 0.3876 |
| 5 | rs6899161 |  | 171922211 |  | G | 0.5203 | 0.2905 | A | 5.70E-05 | 2.648 | 0.3776 |
| 5 | kgp7131605 | rs13189040 | 171928783 |  | A | 0.527 | 0.3041 | G | 9.94E-05 | 2.55 | 0.3922 |
| 5 | kgp4556147 | rs77483990 | 172638617 |  | A | 0.05405 | 0.2162 | G | 4.50E-05 | 0.2071 |  |
| 5 | kgp2741014 | rs57675864 | 174602666 |  | C | 0.1622 | 0.3649 | A | 7.55E-05 | 0.3369 |  |
| 6 | kgp4599108 | rs11759008 | 7258523 |  | C | 0.09459 | 0.3311 | T | 6.69E-07 | 0.2111 |  |
| 6 | rs9270600 |  | 32561693 | HLA-DRB1 | T | 0.3784 | 0.1644 | C | 3.75E-05 | 3.094 | 0.3232 |
| 6 | rs9270665 |  | 32566232 |  | T | 0.3784 | 0.1689 | G | 5.31E-05 | 2.995 | 0.3339 |
| 6 | kgp2592721 | rs617058 | 32574479 |  | T | 0.3919 | 0.1757 | G | 3.70E-05 | 3.024 | 0.3307 |
| 6 | rs674313 |  | 32578082 |  | A | 0.4054 | 0.1959 | G | 8.52E-05 | 2.798 | 0.3574 |
| 6 | rs2858863 |  | 32578278 |  | A | 0.3919 | 0.1757 | G | 3.70E-05 | 3.024 | 0.3307 |
| 6 | rs2858862 |  | 32580321 |  | A | 0.4324 | 0.2095 | G | 3.98E-05 | 2.876 | 0.3488 |
| 6 | rs642093 |  | 32582075 |  | A | 0.3919 | 0.1689 | G | 1.95E-05 | 3.171 | 0.3154 |
| 6 | kgp12577045 | rs9271334 | 32582711 |  | C | 0.4324 | 0.2095 | A | 3.98E-05 | 2.876 | 0.3477 |
| 6 | kgp10931469 | rs28383221 | 32583212 |  | A | 0.527 | 0.2973 | G | 5.95E-05 | 2.634 | 0.3797 |
| 6 | rs9271352 |  | 32583938 |  | C | 0.3784 | 0.1622 | T | 2.81E-05 | 3.145 | 0.3180 |
| 6 | rs9271562 |  | 32590411 |  | A | 0.4459 | 0.2297 | G | 8.41E-05 | 2.699 | 0.3705 |
| 6 | rs2097432 |  | 32590771 |  | G | 0.4247 | 0.1918 | A | 1.64E-05 | 3.111 | 0.3214 |
| 6 | rs3135003 |  | 32591476 | HLA-DQA1 | A | 0.3311 | 0.1351 | G | 6.70E-05 | 3.168 | 0.3157 |
| 6 | rs9271613 |  | 32591666 | HLA-DQA1 | T | 0.3699 | 0.1575 | C | 3.84E-05 | 3.139 | 0.3186 |
| 6 | kgp11901668 | rs1886884 | 44192158 | SLC29A1 | T | 0.3649 | 0.5946 | C | 7.63E-05 | 0.3917 |  |
| 7 | rs3218660 |  | 44112873 | POLM | A | 0.1689 | 0.3986 | C | 1.17E-05 | 0.3066 |  |
| 7 | kgp8575184 | rs11769882 | 44124083 | POLM | A | 0.1486 | 0.3649 | G | 2.07E-05 | 0.3039 |  |
| 7 | rs2711870 |  | 103226907 | RELN | A | 0.2838 | 0.1014 | G | 6.89E-05 | 3.513 | 0.2847 |
| 7 | rs7805374 |  | 120883911 | CPED1 | G | 0.2635 | 0.4865 | T | 7.43E-05 | 0.3777 |  |
| 7 | rs4731007 |  | 120904659 | CPED1 | G | 0.2635 | 0.4865 | A | 7.43E-05 | 0.3777 |  |
| 7 | rs6466899 |  | 123860214 |  | T | 0.3851 | 0.1757 | C | 6.04E-05 | 2.939 | 0.3403 |
| 7 | kgp7916752 | rs201690638 | 127828173 |  | G | 0.6149 | 0.3851 | A | 7.74E-05 | 2.549 | 0.3923 |
| 7 | rs4075517 |  | 135018924 |  | A | 0.25 | 0.07432 | G | 4.13E-05 | 4.152 | 0.2408 |
| 7 | kgp1224246 | rs12707228 | 135050994 | CNOT4 | G | 0.223 | 0.05405 | A | 2.59E-05 | 5.022 | 0.1991 |
| 7 | rs11768962 |  | 135254692 | NUP205 | C | 0.2568 | 0.08108 | T | 5.50E-05 | 3.915 | 0.2554 |
| 7 | kgp11975543 | rs11772708 | 135264737 | NUP205 | G | 0.25 | 0.08108 | A | 9.24E-05 | 3.778 | 0.2646 |
| 7 | kgp5669907 | rs3110778 | 135360106 | C7orf73 | A | 0.4527 | 0.223 | G | 2.94E-05 | 2.883 | 0.3469 |
| 7 | kgp2234782 |  | 135360119 | C7orf73 | T | 0.4527 | 0.223 | C | 2.94E-05 | 2.883 | 0.3469 |
| 7 | rs3110789 |  | 135364526 | C7orf73/SLC13A4 | A | 0.4527 | 0.2095 | G | 8.74E-06 | 3.122 | 0.3203 |
| 8 | kgp9061730 | rs3886414 | 5445567 |  | T | 0.05405 | 0.2095 | G | 7.73E-05 | 0.2157 |  |
| 8 | rs630757 |  | 16709730 |  | A | 0.4122 | 0.2027 | C | 9.43E-05 | 2.758 | 0.3636 |
| 9 | kgp6650316 | rs10975267 | 5737209 | KIAA1432 | C | 0.3243 | 0.5541 | T | 6.83E-05 | 0.3863 |  |
| 9 | rs6477040 |  | 6133074 |  | T | 0.09459 | 0.2838 | C | 3.25E-05 | 0.2637 |  |
| 9 | kgp18611014 | rs148498636 | 30400190 |  | G | 0.006849 | 0.1351 | A | 1.95E-05 | 0.04414 |  |
| 10 | kgp3745966 | rs72776546 | 4184660 |  | T | 0.01351 | 0.1419 | C | 3.70E-05 | 0.08284 |  |
| 10 | rs10508273 |  | 4208924 |  | A | 0.02027 | 0.1486 | C | 7.15E-05 | 0.1185 |  |
| 12 | kgp3290240 | rs201858622 | 66661985 |  | G | 0.3243 | 0.1216 | T | 2.80E-05 | 3.467 | 0.2884 |
| 12 | kgp1796781 | rs289068 | 66662003 |  | C | 0.3108 | 0.1149 | T | 3.82E-05 | 3.475 | 0.2878 |
| 12 | rs289067 |  | 66662280 |  | A | 0.3108 | 0.1216 | G | 7.71E-05 | 3.257 | 0.3070 |
| 12 | kgp7270084 | rs7961390 | 90274233 |  | A | 0.5203 | 0.2973 | G | 9.55E-05 | 2.563 | 0.3902 |
| 12 | rs6538566 |  | 95296558 | NDUFA12 | A | 0.08784 | 0.2635 | C | 7.15E-05 | 0.2691 |  |
| 12 | kgp10698505 | rs73222800 | 121105264 | CABP1 | G | 0 | 0.1014 | A | 7.04E-05 | 0 |  |
| 12 | kgp1733766 | rs77973078 | 128991988 | TMEM132C | A | 0.04054 | 0.1959 | G | 3.47E-05 | 0.1734 |  |
| 12 | kgp2165394 | rs545368 | 129460867 | GLT1D1 | A | 0.4189 | 0.1757 | G | 4.70E-06 | 3.383 | 0.2956 |
| 13 | rs6583 |  | 24881184 | SPATA13/C1QTNF9 | C | 0.2703 | 0.5068 | T | 3.00E-05 | 0.3605 |  |
| 13 | rs2038949 |  | 75206681 |  | C | 0.3784 | 0.1757 | T | 9.77E-05 | 2.856 | 0.3501 |
| 13 | kgp1416341 | rs78364105 | 81657010 |  | A | 0.006757 | 0.1216 | G | 5.54E-05 | 0.04913 |  |
| 13 | kgp676025 | rs80287076 | 81660217 |  | A | 0.006849 | 0.1301 | G | 3.04E-05 | 0.0461 |  |
| 14 | kgp10866663 | rs10483339 | 29025319 |  | T | 0.277 | 0.07432 | A | 4.60E-06 | 4.772 | 0.2096 |
| 14 | rs9322878 |  | 32341906 |  | A | 0.1419 | 0.3378 | G | 7.90E-05 | 0.3241 |  |
| 14 | rs11847817 |  | 39019800 |  | C | 0.1757 | 0.03378 | T | 6.71E-05 | 6.095 | 0.1641 |
| 15 | rs8043208 |  | 26129701 |  | C | 0.1181 | 0.3082 | T | 7.84E-05 | 0.3004 |  |
| 15 | kgp1089843 | rs17326524 | 74458181 |  | C | 0.1233 | 0.006849 | T | 5.50E-05 | 20.39 | 0.0490 |
| 15 | kgp19794435 | rs920562 | 90155428 | TICRR | G | 0 | 0.1149 | A | 2.17E-05 | 0 |  |
| 15 | rs12908968 |  | 93454382 | CHD2 | T | 0.3041 | 0.5347 | C | 6.45E-05 | 0.3802 |  |
| 16 | kgp16411776 | rs59265091 | 5776805 |  | A | 0.1081 | 0 | C | 3.91E-05 | NA |  |
| 16 | kgp6356788 | rs12325596 | 5938861 |  | T | 0.4122 | 0.2027 | C | 9.43E-05 | 2.758 | 0.3626 |
| 16 | rs13338835 |  | 28325940 | SBK1 | C | 0.3446 | 0.5743 | A | 7.33E-05 | 0.3897 |  |
| 16 | kgp3468945 | rs12598207 | 87677386 | JPH3 | A | 0.3311 | 0.1216 | G | 1.66E-05 | 3.575 | 0.2797 |
| 16 | kgp8149184 | rs368146051 | 87677456 | JPH3 | G | 0.3108 | 0.1149 | A | 3.82E-05 | 3.475 | 0.2878 |
| 17 | kgp8229316 | rs2293880 | 3599044 | P2RX5 | T | 0.1233 | 0.3333 | C | 1.99E-05 | 0.2812 |  |
| 17 | rs1550641 |  | 13651661 |  | C | 0.02703 | 0.1757 | T | 2.26E-05 | 0.1303 |  |
| 18 | kgp6002088 |  | 53142005 | TCF4 | T | 0.3311 | 0.5676 | G | 4.32E-05 | 0.3771 |  |
| 18 | rs7233312 |  | 53143573 | TCF4 | T | 0.3041 | 0.5608 | C | 8.26E-06 | 0.3421 |  |
| 19 | kgp709554 | rs12979148 | 19406869 | SUGP1 | C | 0.04054 | 0.223 | T | 3.49E-06 | 0.1472 |  |
| 19 | rs10401969 |  | 19407718 | SUGP1 | C | 0.006757 | 0.1892 | T | 1.30E-07 | 0.02915 |  |
| 19 | kgp9048653 | rs4802022 | 39677989 | PAK4 | G | 0.1736 | 0.03425 | T | 9.76E-05 | 5.924 | 0.1688 |
| 20 | rs6063154 |  | 46455577 |  | T | 0.5274 | 0.2778 | G | 1.48E-05 | 2.901 | 0.3447 |
| 20 | rs6094895 |  | 46495123 |  | T | 0.3986 | 0.1486 | C | 1.41E-06 | 3.797 | 0.2634 |
| 20 | kgp3976754 | rs6125157 | 46496755 |  | C | 0.2429 | 0.07143 | T | 8.11E-05 | 4.17 | 0.2398 |
| 21 | kgp3629758 | rs4819113 | 46868710 | COL18A1 | A | 0.08784 | 0.2703 | G | 4.25E-05 | 0.26 |  |
| 21 | rs2183590 |  | 46872167 | COL18A1 | G | 0.08784 | 0.2703 | A | 4.25E-05 | 0.26 |  |
| 22 | kgp22834527 | rs12166565 | 33057937 | SYN3 | G | 0.05405 | 0.2095 | A | 7.73E-05 | 0.2157 |  |

**Table E. Lowest p-values in the association test in all SNPs in the OCG.** NA – not assigned. MAF in the 222 individuals higher or equal to 5%.

| Chr | ID in the chip | ID in Ensembl | Position in GRCh37 | Gene | Allele | Frequency in hemorrhagic fever | Frequency in asymptomatic control | Other allele | p-value | Odds ratio | Odds ratio alternative allele |
| --- | --- | --- | --- | --- | --- | --- | --- | --- | --- | --- | --- |
| 1 | rs4949509 | rs4949509 | 30351529 |  | C | 0.3604 | 0.1937 | T | 0.00008722 | 2.345 | 0.4264392 |
| 1 | rs553133 | rs553133 | 58700416 | DAB1 | A | 0.5811 | 0.3739 | C | 0.00001239 | 2.323 | 0.4304778 |
| 1 | kgp2740709 | rs626282 | 58700871 | DAB1 | C | 0.5856 | 0.3964 | T | 0.00006688 | 2.152 | 0.464684 |
| 1 | rs4912184 | rs4912184 | 58704644 | DAB1 | C | 0.4775 | 0.2973 | A | 0.00009749 | 2.16 | 0.462963 |
| 1 | kgp7365728 | rs495179 | 58707234 | DAB1 | T | 0.4189 | 0.2364 | C | 0.00004365 | 2.329 | 0.4293688 |
| 1 | kgp7932404 | rs10493249 | 58772127 | DAB1 | T | 0.2227 | 0.07658 | G | 0.00001628 | 3.455 | 0.2894356 |
| 1 | rs12567514 | rs12567514 | 58800475 | DAB1 | C | 0.2342 | 0.07658 | T | 0.000004544 | 3.689 | 0.2710762 |
| 1 | kgp10767894 | rs10873997 | 79263030 |  | G | 0.4144 | 0.2117 | A | 0.000004121 | 2.635 | 0.3795066 |
| 1 | kgp9192569 | rs61800490 | 157353129 |  | A | 0.1847 | 0.3694 | G | 0.00001375 | 0.3867 |  |
| 1 | kgp7064588 | rs11589226 | 157359491 |  | G | 0.1712 | 0.3636 | A | 0.000004799 | 0.3614 |  |
| 1 | rs12073794 | rs12073794 | 157375078 |  | T | 0.1698 | 0.3472 | C | 0.00002826 | 0.3845 |  |
| 1 | rs4376717 | rs4376717 | 194347944 |  | T | 0.02703 | 0.1261 | G | 0.00008626 | 0.1925 |  |
| 1 | rs10863669 | rs10863669 | 222097527 |  | T | 0.3288 | 0.518 | C | 0.00005482 | 0.4559 |  |
| 1 | rs17463586 | rs17463586 | 222719037 | HHIPL2 | A | 0.5909 | 0.4009 | G | 0.00006474 | 2.159 | 0.4631774 |
| 1 | rs1155739 | rs1155739 | 233204338 | PCNXL2 | C | 0.2928 | 0.4775 | A | 0.00006375 | 0.4531 |  |
| 1 | rs6424276 | rs6424276 | 233214528 | PCNXL2 | C | 0.2838 | 0.4685 | A | 0.000059 | 0.4496 |  |
| 2 | kgp8608378 | rs7579897 | 13069140 |  | G | 0.4685 | 0.2793 | A | 0.00003794 | 2.274 | 0.4397537 |
| 2 | rs12465302 | rs12465302 | 23585867 |  | A | 0.235 | 0.075 | G | 0.000009822 | 3.789 | 0.2639219 |
| 2 | kgp9646386 | rs12622644 | 23601593 |  | A | 0.2568 | 0.0991 | G | 0.00001405 | 3.14 | 0.3184713 |
| 2 | rs4145831 | rs4145831 | 33700305 | RASGRP3 | C | 0.1955 | 0.06881 | T | 0.00009233 | 3.288 | 0.3041363 |
| 2 | kgp3310635 | NA | 54467065 | ACYP2 | T | 0.1727 | 0.3364 | G | 0.00008152 | 0.4119 |  |
| 2 | kgp1289193 | rs2628196 | 54493335 | ACYP2 | A | 0.1982 | 0.3649 | T | 0.0000945 | 0.4303 |  |
| 2 | rs10177225 | rs10177225 | 61594498 | USP34 | T | 0.3919 | 0.5901 | C | 0.00002954 | 0.4477 |  |
| 2 | rs10206850 | rs10206850 | 138420996 | THSD7B | A | 0.3604 | 0.545 | G | 0.00009265 | 0.4703 |  |
| 2 | rs7589528 | rs7589528 | 154420012 |  | G | 0.0991 | 0.2387 | A | 0.00008618 | 0.3508 |  |
| 2 | rs2892792 | rs2892792 | 162640725 | SLC4A10 | T | 0.1532 | 0.3198 | C | 0.0000359 | 0.3846 |  |
| 2 | kgp2397725 | rs6730118 | 170205350 | LRP2 | G | 0.1577 | 0.3198 | A | 0.00006134 | 0.3981 |  |
| 2 | rs4972452 | rs4972452 | 175467910 | WIPF1 | T | 0.3739 | 0.5631 | G | 0.00006485 | 0.4634 |  |
| 2 | kgp9542830 | rs6729176 | 175475176 | WIPF1 | C | 0.3899 | 0.5872 | T | 0.00003793 | 0.4494 |  |
| 2 | kgp7216668 | rs35112591 | 179543217 | TTN/TTN-AS1 | T | 0.2162 | 0.08108 | C | 0.00006277 | 3.126 | 0.3198976 |
| 2 | rs777715 | rs777715 | 195955184 |  | T | 0.2568 | 0.1126 | C | 0.00009092 | 2.722 | 0.3673769 |
| 2 | rs10932212 | rs10932212 | 208735106 | PLEKHM3 | T | 0.1847 | 0.3514 | C | 0.00007358 | 0.4182 |  |
| 3 | kgp9483807 | rs200704287 | 16407519 | RFTN1 | T | 0.07207 | 0.2387 | C | 0.000001255 | 0.2477 |  |
| 3 | kgp9854759 | rs79691057 | 16408251 | RFTN1 | A | 0.07207 | 0.2387 | G | 0.000001255 | 0.2477 |  |
| 3 | kgp17955983 | rs75180423 | 16408723 | RFTN1 | A | 0.07207 | 0.2364 | C | 0.000001697 | 0.2509 |  |
| 3 | kgp12265183 | rs17201070 | 18117384 | TBC1D5 | G | 0.08636 | 0.2297 | A | 0.00003662 | 0.3169 |  |
| 3 | rs11128951 | rs11128951 | 20375546 |  | G | 0.2703 | 0.1227 | A | 0.00009591 | 2.647 | 0.3777862 |
| 3 | rs1876168 | rs1876168 | 31039428 |  | G | 0.3964 | 0.5856 | A | 0.00006688 | 0.4648 |  |
| 3 | rs1567025 | rs1567025 | 31042458 |  | C | 0.3694 | 0.5676 | T | 0.00002856 | 0.4463 |  |
| 3 | kgp117611 | rs7642265 | 31049205 |  | G | 0.367 | 0.5586 | T | 0.00005596 | 0.4582 |  |
| 3 | rs7649557 | rs7649557 | 70450505 |  | G | 0.5856 | 0.4009 | A | 0.00009946 | 2.112 | 0.4734848 |
| 3 | kgp11374004 | rs75011914 | 73781759 |  | A | 0.08108 | 0.2162 | G | 0.00006277 | 0.3199 |  |
| 3 | rs17756459 | rs17756459 | 73786057 |  | T | 0.09009 | 0.2252 | G | 0.0000935 | 0.3406 |  |
| 3 | rs7631627 | rs7631627 | 73861226 |  | A | 0.2387 | 0.0991 | G | 0.00008618 | 2.851 | 0.3507541 |
| 3 | kgp8835486 | rs13089675 | 74776213 |  | A | 0.2297 | 0.4144 | G | 0.00003125 | 0.4214 |  |
| 3 | rs7635628 | rs7635628 | 74783321 |  | A | 0.2477 | 0.4279 | G | 0.0000598 | 0.4403 |  |
| 3 | kgp3776032 | rs1921440 | 74784754 |  | T | 0.2477 | 0.4279 | C | 0.0000598 | 0.4403 |  |
| 3 | kgp6579124 | rs60272712 | 119732508 | GSK3B | A | 0.1937 | 0.06757 | G | 0.00008042 | 3.315 | 0.3016591 |
| 3 | rs1445221 | rs1445221 | 135049624 |  | G | 0.2252 | 0.3964 | A | 0.00009759 | 0.4427 |  |
| 3 | kgp18045791 | rs76056700 | 178193821 | KCNMB2 | A | 0.1171 | 0.02252 | G | 0.00009203 | 5.757 | 0.1737016 |
| 4 | rs11940485 | rs11940485 | 22256034 |  | G | 0.5766 | 0.3773 | T | 0.00002736 | 2.248 | 0.4448399 |
| 4 | kgp4663656 | rs12646653 | 40913318 | APBB2 | C | 0.3409 | 0.1757 | T | 0.00007183 | 2.427 | 0.4120313 |
| 4 | kgp8989108 | rs13138844 | 40917128 | APBB2 | C | 0.3468 | 0.1802 | T | 0.00006722 | 2.416 | 0.4139073 |
| 4 | kgp10915301 | rs72910257 | 114023278 | ANK2 | A | 0.1081 | 0.01802 | G | 0.00009431 | 6.606 | 0.1513775 |
| 4 | kgp5870923 | rs59147579 | 139564705 |  | G | 0.5676 | 0.3829 | A | 0.00009766 | 2.115 | 0.4728132 |
| 4 | kgp1673078 | rs78968228 | 158609897 |  | G | 0.1171 | 0.02252 | A | 0.00009203 | 5.757 | 0.1737016 |
| 4 | kgp7665007 | rs35565048 | 170064666 | SH3RF1 | T | 0.009009 | 0.1081 | C | 0.000008719 | 0.075 |  |
| 4 | kgp1113254 | rs67620067 | 170085697 | SH3RF1 | A | 0.009009 | 0.1036 | G | 0.00001536 | 0.07866 |  |
| 5 | kgp5662036 | rs16879046 | 7781069 | ADCY2 | T | 0.1577 | 0.04505 | C | 0.00008448 | 3.968 | 0.2520161 |
| 5 | kgp5151023 | rs12523098 | 24020014 | C5orf17 | T | 0.01351 | 0.1036 | C | 0.00005289 | 0.1185 |  |
| 5 | rs6871776 | rs6871776 | 24036642 | C5orf17 | C | 0.01351 | 0.1036 | T | 0.00005289 | 0.1185 |  |
| 5 | kgp14150 | rs10461906 | 31998191 | PDZD2 | C | 0.1591 | 0.3243 | T | 0.00005015 | 0.3941 |  |
| 5 | kgp8931326 | rs34286796 | 73933174 | ENC1/HEXB | C | 0.2117 | 0.08108 | A | 0.00009891 | 3.044 | 0.3285151 |
| 6 | kgp4599108 | rs11759008 | 7258523 |  | C | 0.1532 | 0.3108 | T | 0.00008314 | 0.401 |  |
| 6 | rs4618530 | rs4618530 | 16795923 |  | G | 0.3468 | 0.1757 | A | 0.00004044 | 2.492 | 0.4012841 |
| 6 | kgp521176 | rs16879224 | 16803120 |  | A | 0.2973 | 0.1441 | C | 0.00009999 | 2.512 | 0.3980892 |
| 6 | rs2517917 | rs2517917 | 29781020 | MICG | G | 0.1455 | 0.3045 | A | 0.00006449 | 0.3887 |  |
| 6 | rs625051 | rs625051 | 79268831 |  | G | 0.3559 | 0.5495 | T | 0.00004136 | 0.4528 |  |
| 6 | kgp10256432 | rs654628 | 79287086 |  | G | 0.3559 | 0.5405 | A | 0.00009131 | 0.4696 |  |
| 6 | rs236875 | rs236875 | 79304684 |  | A | 0.3153 | 0.5405 | C | 0.00000162 | 0.3914 |  |
| 6 | rs236877 | rs236877 | 79305484 |  | G | 0.5676 | 0.3649 | A | 0.00001861 | 2.285 | 0.4376368 |
| 6 | kgp9904225 | rs70478 | 79308180 |  | T | 0.2568 | 0.482 | C | 8.808E-07 | 0.3713 |  |
| 6 | rs299416 | rs299416 | 114427189 | HS3ST5 | G | 0.05405 | 0.1757 | A | 0.00005855 | 0.2681 |  |
| 6 | rs1408277 | rs1408277 | 121811989 |  | G | 0.08108 | 0.2207 | A | 0.00003956 | 0.3115 |  |
| 6 | kgp272848 | rs4629700 | 123395606 | CLVS2 | A | 0.289 | 0.1364 | G | 0.0000941 | 2.574 | 0.3885004 |
| 6 | kgp2910087 | rs3799606 | 166993078 | RPS6KA2 | T | 0.133 | 0.3257 | A | 0.000001715 | 0.3177 |  |
| 7 | kgp1374146 | rs59082935 | 38724868 | FAM183B | T | 0.1955 | 0.06757 | C | 0.00006848 | 3.353 | 0.2982404 |
| 7 | rs1464888 | rs1464888 | 53155331 |  | C | 0.491 | 0.3063 | T | 0.00007066 | 2.185 | 0.4576659 |
| 7 | rs17142110 | rs17142110 | 67122981 |  | T | 0.1306 | 0.02703 | C | 0.00005108 | 5.409 | 0.1848771 |
| 7 | rs9785984 | rs9785984 | 91108429 |  | T | 0.0991 | 0.3102 | C | 4.042E-08 | 0.2446 |  |
| 7 | kgp2753217 | rs2299276 | 95665963 | DYNC1I1 | A | 0.1712 | 0.3468 | G | 0.00002391 | 0.3889 |  |
| 7 | kgp8157861 | rs1048666 | 95668664 | DYNC1I1 | A | 0.1577 | 0.3288 | C | 0.0000263 | 0.382 |  |
| 7 | rs4731007 | rs4731007 | 120904659 | CPED1 | G | 0.2883 | 0.4685 | A | 0.00009071 | 0.4596 |  |
| 7 | rs6466899 | rs6466899 | 123860214 |  | T | 0.3559 | 0.1802 | C | 0.00002932 | 2.514 | 0.3977725 |
| 8 | rs630757 | rs630757 | 16709730 |  | A | 0.4045 | 0.2297 | C | 0.00007811 | 2.278 | 0.4389816 |
| 8 | kgp3568407 | rs9644119 | 26517817 |  | T | 0.3333 | 0.1712 | G | 0.0000836 | 2.421 | 0.4130525 |
| 8 | rs11783368 | rs11783368 | 26541693 |  | A | 0.3559 | 0.1892 | G | 0.00008025 | 2.368 | 0.4222973 |
| 8 | rs889981 | rs889981 | 57775940 |  | T | 0.4676 | 0.2838 | C | 0.00007073 | 2.217 | 0.45106 |
| 8 | kgp8462291 | rs73250525 | 65782120 |  | A | 0.1351 | 0.2864 | G | 0.00009614 | 0.3894 |  |
| 8 | rs2953950 | rs2953950 | 69693543 | C8orf34 | C | 0.5901 | 0.3964 | A | 0.0000447 | 2.192 | 0.4562044 |
| 8 | kgp10244713 | rs2978242 | 69693958 | C8orf34 | A | 0.5901 | 0.3964 | G | 0.0000447 | 2.192 | 0.4562044 |
| 8 | rs10091347 | rs10091347 | 69799082 |  | C | 0.1396 | 0.2928 | T | 0.00008868 | 0.392 |  |
| 8 | kgp6669808 | rs72606654 | 91259303 |  | C | 0.1757 | 0.05405 | T | 0.00005855 | 3.73 | 0.2680965 |
| 9 | kgp7188202 | rs10969131 | 2949732 | CARM1P1 | G | 0.4685 | 0.2748 | A | 0.00002411 | 2.326 | 0.4299226 |
| 9 | kgp4161004 | rs66837256 | 122956926 |  | G | 0.04505 | 0.1577 | A | 0.00008448 | 0.252 |  |
| 10 | rs11005218 | rs11005218 | 57868529 |  | G | 0.1667 | 0.04955 | T | 0.00007076 | 3.836 | 0.2606882 |
| 10 | kgp9476777 | rs10829356 | 129999182 |  | G | 0.2748 | 0.1261 | A | 0.00009154 | 2.625 | 0.3809524 |
| 11 | rs11034573 | rs11034573 | 4919979 | MMP26 | G | 0.06818 | 0.2072 | A | 0.00002265 | 0.28 |  |
| 11 | kgp3055458 | rs10768101 | 34811218 |  | G | 0.03636 | 0.1441 | A | 0.00007845 | 0.2241 |  |
| 11 | kgp553120 | rs4756172 | 34817156 |  | A | 0.03153 | 0.1351 | G | 0.00007838 | 0.2084 |  |
| 11 | kgp5991453 | rs10791070 | 130240541 |  | T | 0.3559 | 0.5495 | C | 0.00004136 | 0.4528 |  |
| 11 | rs10736582 | rs10736582 | 130241003 |  | T | 0.3559 | 0.5495 | C | 0.00004136 | 0.4528 |  |
| 11 | kgp7746757 | rs4937504 | 130247700 |  | G | 0.2838 | 0.4727 | A | 0.00004196 | 0.4419 |  |
| 12 | kgp6649501 | rs10332 | 29655637 | TMTC1 | T | 0.1577 | 0.3468 | C | 0.000004444 | 0.3525 |  |
| 13 | kgp22825086 | rs201729933 | 63954193 |  | T | 0.2909 | 0.473 | C | 0.00008209 | 0.4571 |  |
| 13 | rs4884473 | rs4884473 | 63957127 |  | T | 0.2883 | 0.473 | C | 0.00006138 | 0.4514 |  |
| 13 | kgp8553006 | rs9528615 | 63962296 |  | G | 0.2793 | 0.4595 | A | 0.00008381 | 0.4559 |  |
| 13 | kgp6408373 | rs2324286 | 63962844 |  | C | 0.2793 | 0.4595 | T | 0.00008381 | 0.4559 |  |
| 13 | rs1475142 | rs1475142 | 109500374 | MYO16 | G | 0.4545 | 0.2703 | A | 0.00005561 | 2.25 | 0.4444444 |
| 14 | rs11160462 | rs11160462 | 20868751 | TEP1 | A | 0.3559 | 0.1892 | G | 0.00008025 | 2.368 | 0.4222973 |
| 14 | kgp10866663 | rs10483339 | 29025319 |  | T | 0.2207 | 0.08559 | A | 0.00007707 | 3.026 | 0.3304693 |
| 14 | rs12435683 | rs12435683 | 82292820 |  | A | 0.473 | 0.2928 | G | 0.00009412 | 2.168 | 0.4612546 |
| 14 | kgp10568878 | rs77348321 | 105060880 | TMEM179/C14orf180 | T | 0.1937 | 0.06422 | C | 0.00005262 | 3.5 | 0.2857143 |
| 14 | rs2582492 | rs2582492 | 105443695 | AHNAK2 | A | 0.3194 | 0.5091 | G | 0.00005868 | 0.4526 |  |
| 14 | rs2033932 | rs2033932 | 105447075 | AHNAK2 | A | 0.2 | 0.3727 | G | 0.0000614 | 0.4207 |  |
| 15 | kgp2068147 | rs4776016 | 52498360 | MYO5C | G | 0.5405 | 0.3559 | A | 0.00009131 | 2.13 | 0.4694836 |
| 15 | rs11070884 | rs11070884 | 52501831 | MYO5C | A | 0.3761 | 0.5682 | G | 0.00005698 | 0.4582 |  |
| 15 | kgp7042899 | rs4774612 | 52511465 | MYO5C | T | 0.2162 | 0.08108 | C | 0.00006277 | 3.126 | 0.3198976 |
| 15 | kgp8249459 | rs1500631 | 98079217 |  | C | 0.1622 | 0.04054 | T | 0.00002178 | 4.581 | 0.2182929 |
| 15 | kgp3218668 | rs12324742 | 98081991 |  | C | 0.1622 | 0.04054 | A | 0.00002178 | 4.581 | 0.2182929 |
| 15 | rs1840300 | rs1840300 | 100366944 |  | A | 0.3909 | 0.5946 | G | 0.00001847 | 0.4376 |  |
| 16 | rs10521060 | rs10521060 | 3620927 | NLRC3 | C | 0.02703 | 0.1261 | T | 0.00008626 | 0.1925 |  |
| 16 | kgp1234511 | rs2159307 | 5940948 |  | C | 0.2793 | 0.464 | T | 0.00005661 | 0.4477 |  |
| 16 | rs13338835 | rs13338835 | 28325940 | SBK1 | C | 0.3739 | 0.5766 | A | 0.00001899 | 0.4385 |  |
| 16 | rs282957 | rs282957 | 77533525 |  | T | 0.2207 | 0.4144 | C | 0.00001167 | 0.4002 |  |
| 16 | rs11861812 | rs11861812 | 83473113 | CDH13 | T | 0.1982 | 0.05909 | C | 0.00001283 | 3.936 | 0.254065 |
| 17 | rs12936861 | rs12936861 | 11665034 | DNAH9 | T | 0.2432 | 0.4234 | G | 0.00005651 | 0.4377 |  |
| 17 | kgp4942059 | rs7207154 | 78454756 | NPTX1 | C | 0.05046 | 0.1682 | T | 0.00008029 | 0.2628 |  |
| 18 | rs16941779 | rs16941779 | 23307937 |  | G | 0.3018 | 0.4955 | A | 0.00003074 | 0.4401 |  |
| 18 | rs2277720 | rs2277720 | 56165164 | ALPK2 | T | 0.2207 | 0.07658 | C | 0.00001964 | 3.416 | 0.29274 |
| 19 | kgp11414451 | NA | 11275394 | KANK2 | A | 0.545 | 0.3604 | C | 0.00009265 | 2.126 | 0.4703669 |
| 19 | kgp9826137 | rs9636166 | 31829613 | TSHZ3 | C | 0.07658 | 0.2091 | A | 0.00006763 | 0.3137 |  |
| 19 | kgp21470650 | rs201678012 | 31829752 | TSHZ3 | T | 0.09009 | 0.2523 | C | 0.000005736 | 0.2935 |  |
| 19 | rs10418984 | rs10418984 | 31838378 | TSHZ3 | T | 0.0991 | 0.2477 | C | 0.00003527 | 0.334 |  |
| 20 | rs227130 | rs227130 | 8432959 | PLCB1 | A | 0.2883 | 0.473 | G | 0.00006138 | 0.4514 |  |
| 20 | kgp10613555 | rs2207312 | 8439060 | PLCB1 | C | 0.3559 | 0.5495 | G | 0.00004136 | 0.4528 |  |
| 20 | kgp819345 | rs2234197 | 43589041 | TOMM34/PABPC1L | A | 0.1126 | 0.2568 | G | 0.00009092 | 0.3674 |  |
| 21 | rs6517137 | rs6517137 | 34400779 | OLIG2 | C | 0.1802 | 0.04955 | T | 0.00001587 | 4.216 | 0.2371917 |
| 21 | rs9976404 | rs9976404 | 35551600 |  | C | 0.3919 | 0.5811 | A | 0.00006666 | 0.4646 |  |
| 21 | rs2836989 | rs2836989 | 40733567 |  | G | 0.1892 | 0.06306 | A | 0.00006266 | 3.467 | 0.2884338 |
| 22 | rs16986747 | rs16986747 | 27466842 |  | A | 0.2297 | 0.4054 | G | 0.00007014 | 0.4374 | 2.2862369 |


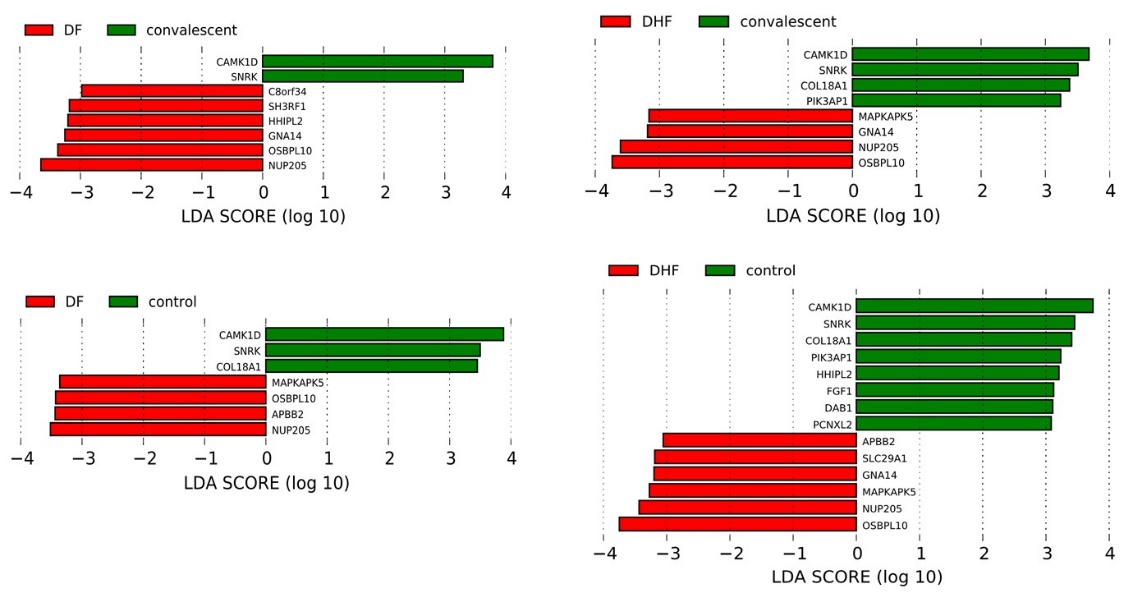


**Fig G. LefSe for African-related associated genes with dengue fever in the three comparison groups (HCG, FCG and OCG).**


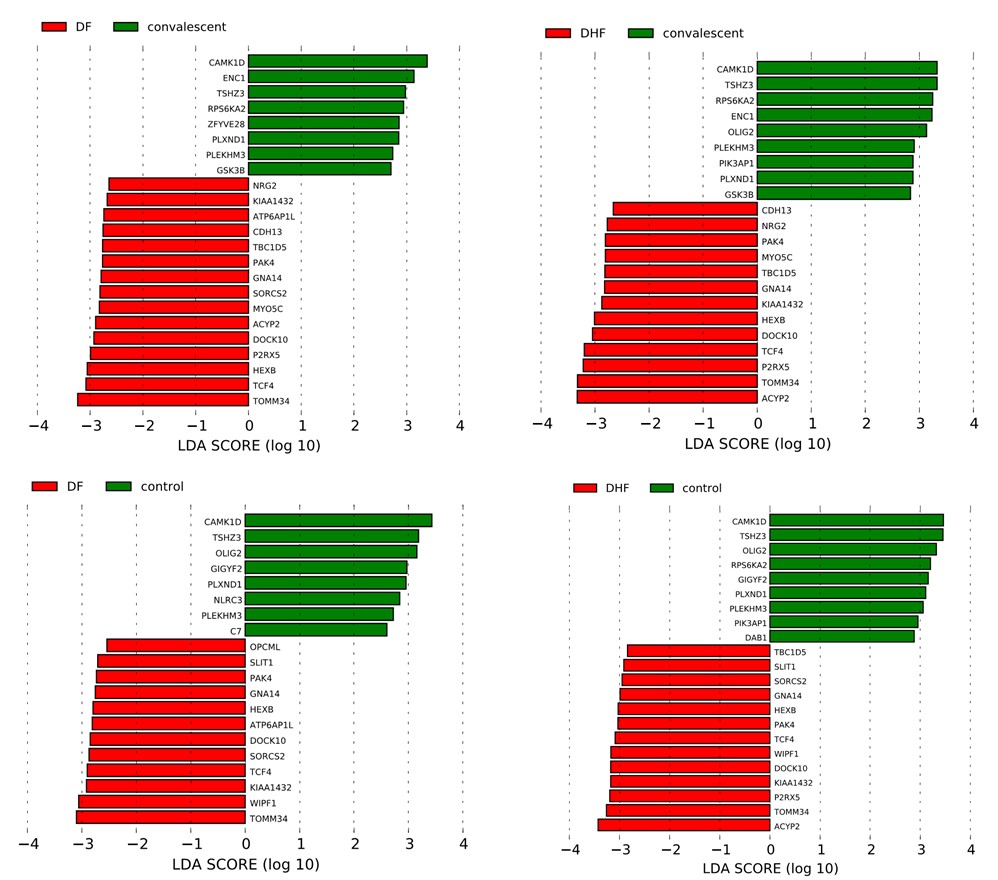


**Fig H. LefSe for non-African-related associated genes with dengue fever in the three comparison groups (HCG, FCG and OCG).**

*1.5- Admixture mapping*

Figs I-K and Tables F-H report theresults of the admixture mapping performed in HCG, FCG and OCG.

**Fig I. Difference in African ancestry in HCG along the 22 autosomes.** The line defines the 99% confidence interval.

**Fig J. Difference in African ancestry in the FCG along the 22 autosomes.** The line defines the 99% confidence interval.


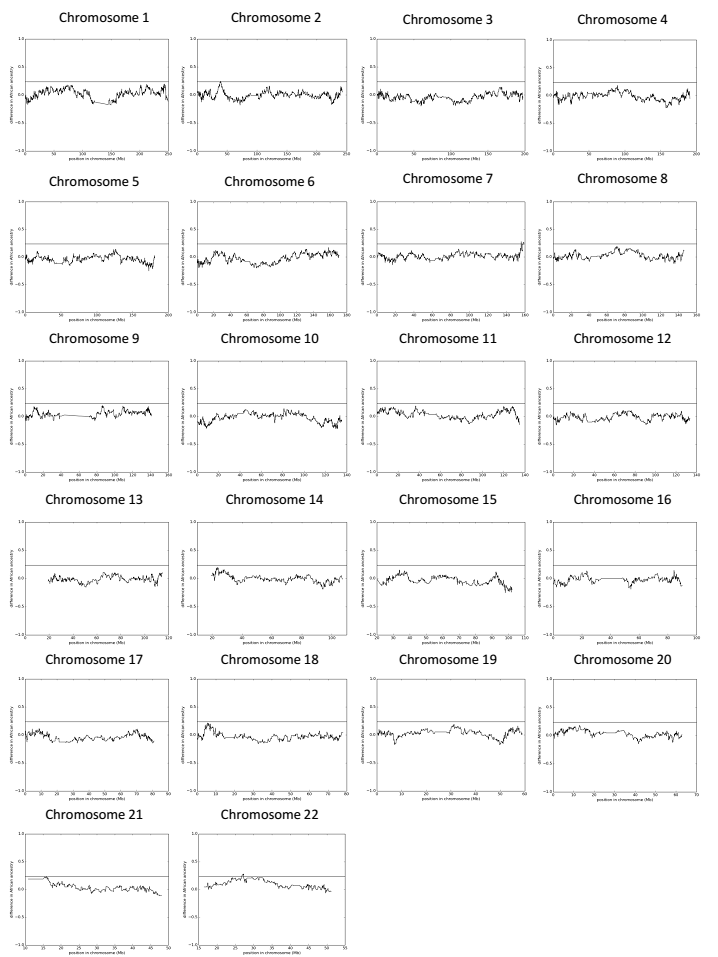


**Fig K. Difference in African ancestry in the OCG along the 22 autosomes.** The line defines the 99% confidence interval.

**Table F. Regions along chromosomes identified in RFMix analysis in the HCG above the 99% confidence interval for the difference in African ancestry.**

| Chr | Block start | Block end | No. of African blocks in asymptomatic control | No. of African blocks in hemorrhagic | p-value | Protein coding genes |
| --- | --- | --- | --- | --- | --- | --- |
| 1 | 79666332 | 80267726 | 33 | 18 | 0.024319984 |  |
| 1 | 80269886 | 80916102 | 33 | 17 | 0.015016031 |  |
| 1 | 80921812 | 81159163 | 34 | 17 | 0.009941148 |  |
| 1 | 81159521 | 81352249 | 32 | 17 | 0.022331877 |  |
| 1 | 81655249 | 81768121 | 33 | 18 | 0.024319984 |  |
| 1 | 81768971 | 81892985 | 33 | 17 | 0.015016031 |  |
| 1 | 81895097 | 81989420 | 31 | 16 | 0.020339182 |  |
| 1 | 211798430 | 212093158 | 33 | 18 | 0.024319984 | NEK2 LPGAT1 |
| 1 | 229499636 | 238391155 | 32 | 17 | 0.022331877 | ACTA1 NUP133 ABCB10 TAF5L C1orf198 URB2 GALNT2 PGBD5 COG2 AGT CAPN9 TTC13 ARV1 FAM89A TSNAX DISC1 TRIM67 C1orf131 GNPAT EXOC8 SPRTN EGLN1 DISC2 SIPA1L2 MAP10 NTPCR PCNXL2 KIAA1804 KCNK1 AK054726 SLC35F3 COA6 TARBP1 IRF2BP2 TOMM20 RBM34 ARID4B GGPS1 TBCE B3GALNT2 AX747026 LYST GNG4 GPR137B NID1 ERO1LB EDARADD LGALS8 HEATR1 ACTN2 MTR MT1HL1 RYR2 ZP4 |
| 1 | 242010116 | 242077448 | 27 | 12 | 0.01257109 | EXO1 |
| 2 | 134054312 | 134213436 | 28 | 13 | 0.014454423 | NCKAP5 |
| 2 | 134363401 | 134589967 | 29 | 14 | 0.01638702 |  |
| 2 | 134591340 | 134643199 | 30 | 14 | 0.010725139 |  |
| 2 | 137591618 | 137769652 | 29 | 14 | 0.01638702 | THSD7B |
| 7 | 155921264 | 155967382 | 35 | 20 | 0.028242831 |  |
| 7 | 158069264 | 158176618 | 36 | 19 | 0.012049956 | PTPRN2 |
| 7 | 158178405 | 158536345 | 36 | 21 | 0.030160496 | PTPRN2 NCAPG2 ESYT2 |
| 7 | 158763288 | 159124481 | 37 | 22 | 0.032039229 | VIPR2 |
| 9 | 86676854 | 86771998 | 35 | 20 | 0.028242831 |  |
| 9 | 86988321 | 87289280 | 34 | 19 | 0.02629332 | NTRK2 |
| 9 | 87292328 | 87530935 | 35 | 20 | 0.028242831 | NTRK2 |
| 9 | 87694716 | 87939277 | 34 | 19 | 0.02629332 |  |
| 9 | 137516213 | 137694369 | 34 | 19 | 0.02629332 | RXRA-COL5A1 |
| 9 | 137699002 | 137730956 | 34 | 17 | 0.009941148 | COL5A1 |
| 9 | 137732458 | 137780823 | 33 | 18 | 0.024319984 | FCN2 COL5A1 |
| 9 | 138668073 | 138817178 | 35 | 19 | 0.017938274 | KCNT1 CAMSAP1 |
| 14 | 24114490 | 24159882 | 30 | 14 | 0.010725139 | DHRS2 |

**Table G. Regions along chromosomes identified in RFMix analysis in the FCG above the 99% confidence interval for the difference in African ancestry.**

| Chr | Block start | Block end | No. of African blocks in asymptomatic control | No. of African blocks in fever | p-value | Protein coding genes |
| --- | --- | --- | --- | --- | --- | --- |
| 2 | 38131976 | 38491993 | 56 | 38 | 0.033508865 | CYP1B1 RMDN2 |
| 2 | 38492587 | 38654657 | 57 | 38 | 0.024758922 | ATL2 |
| 2 | 38871161 | 38956947 | 59 | 39 | 0.018711642 | GALM |
| 2 | 38960491 | 39748858 | 58 | 39 | 0.025559912 | MAP4K3 GEMIN6 ARHGEF33 GALM MORN2 SRSF7 DHX57 SOS1 CDKL4 |
| 2 | 39751029 | 40009531 | 58 | 39 | 0.025559912 | TMEM178A THUMPD2 |
| 2 | 116414990 | 117135584 | 47 | 29 | 0.023337755 | DPP10 |
| 2 | 121481056 | 121562595 | 51 | 33 | 0.028072356 | GLI2 |
| 4 | 187415755 | 187586210 | 51 | 32 | 0.019551431 | MTNR1A FAT1 |
| 7 | 155844449 | 155920358 | 59 | 40 | 0.026338125 |  |
| 7 | 155921264 | 155967382 | 60 | 39 | 0.013541145 |  |
| 7 | 155968611 | 156037378 | 60 | 38 | 0.009319363 |  |
| 7 | 156039150 | 156042560 | 60 | 37 | 0.006295188 |  |
| 7 | 156043710 | 156107390 | 60 | 38 | 0.009319363 |  |
| 7 | 156112148 | 156232935 | 58 | 37 | 0.012562033 |  |
| 7 | 156236883 | 156268109 | 56 | 37 | 0.023936394 |  |
| 7 | 157653065 | 157744289 | 59 | 39 | 0.018711642 | PTPRN2 |
| 7 | 157744752 | 157784649 | 58 | 39 | 0.025559912 | PTPRN2 |
| 7 | 157785543 | 157873975 | 57 | 38 | 0.024758922 | PTPRN2 |
| 7 | 157875083 | 158028901 | 58 | 38 | 0.018084008 | PTPRN2 |
| 7 | 158031109 | 158064729 | 59 | 37 | 0.008944187 | PTPRN2 |
| 7 | 158069264 | 158176618 | 62 | 39 | 0.006850041 | PTPRN2 |
| 7 | 158178405 | 158759602 | 62 | 38 | 0.004585764 | PTPRN2 NCAPG2 ESYT2 WDR60 |
| 7 | 158763288 | 158933239 | 61 | 38 | 0.006575204 | VIPR2 |
| 7 | 158940121 | 159124481 | 61 | 38 | 0.006575204 |  |
| 8 | 145502926 | 145635313 | 52 | 33 | 0.020459321 | TMEM249 CPSF1 SLC52A2 FBXL6 DGAT1 HSF1 SCRT1 BOP1 ADCK5 |
| 8 | 145637148 | 146294106 | 52 | 34 | 0.029208899 | ZNF250 ZNF7 COMMD5 CYHR1 ARHGAP39 ZNF251 FOXH1 LRRC14 VPS28 SLC39A4 PPP1R16A C8orf33 RECQL4 ZNF16 KIFC2 ZNF252P LRRC24 ZNF34 ZNF517 TONSL RPL8 C8orf82 MFSD3 GPT TMED10P1 |
| 9 | 9793846 | 9893071 | 56 | 37 | 0.023936394 | PTPRD |
| 9 | 137732458 | 137780823 | 56 | 38 | 0.033508865 | FCN2 COL5A1 |
| 18 | 4478414 | 4511540 | 58 | 40 | 0.035489905 |  |
| 18 | 5564481 | 5615156 | 59 | 40 | 0.026338125 | EPB41L3 |
| 18 | 5615842 | 5836382 | 59 | 41 | 0.036433749 | EPB41L3 |
| 18 | 6388022 | 6429648 | 58 | 39 | 0.025559912 | L3MBTL4 |
| 18 | 7061984 | 7103146 | 57 | 38 | 0.024758922 | LAMA1 |
| 18 | 7105988 | 7132587 | 59 | 37 | 0.008944187 | LAMA1 |
| 18 | 7133686 | 7172388 | 55 | 37 | 0.032474033 |  |
| 21 | 15909251 | 15959473 | 54 | 36 | 0.031411422 | SAMSN1 |
| 22 | 23062864 | 23062864 | 48 | 30 | 0.024545127 |  |
| 22 | 23872936 | 23932181 | 51 | 32 | 0.019551431 | IGLL1 |
| 22 | 23933452 | 23998474 | 50 | 30 | 0.012618585 | DRICH1 GUSBP11 |
| 22 | 24184850 | 24340940 | 51 | 32 | 0.019551431 | GSTTP1 GSTT2 SLC2A11 MIF DDTL DDT GSTT2B |
| 22 | 25286983 | 25373391 | 48 | 30 | 0.024545127 | SGSM1 TMEM211 |
| 22 | 25373985 | 25415632 | 49 | 31 | 0.025738293 |  |
| 22 | 26737544 | 26818675 | 52 | 33 | 0.020459321 | SEZ6L |
| 22 | 26821469 | 26974741 | 52 | 32 | 0.014037891 | HPS4 ASPHD2 TFIP11 SRRD TPST2 |
| 22 | 26975517 | 27149768 | 52 | 31 | 0.009427431 | MIAT TPST2 CRYBB1 CRYBA4 |
| 22 | 27152964 | 27158663 | 51 | 30 | 0.008890401 |  |
| 22 | 27159832 | 27192401 | 52 | 31 | 0.009427431 |  |
| 22 | 27193844 | 27208422 | 55 | 33 | 0.007387108 |  |
| 22 | 27210691 | 27292868 | 54 | 33 | 0.010494815 |  |
| 22 | 27644841 | 27662213 | 51 | 33 | 0.028072356 |  |
| 22 | 27662934 | 27680780 | 51 | 33 | 0.028072356 |  |
| 22 | 28145214 | 28173898 | 52 | 32 | 0.014037891 | MN1 |
| 22 | 28174632 | 28191895 | 52 | 31 | 0.009427431 | MN1 |
| 22 | 28198577 | 29239766 | 52 | 33 | 0.020459321 | PITPNB CHEK2 XBP1 CCDC117 TTC28 HSCB |
| 22 | 29244395 | 29456360 | 52 | 34 | 0.029208899 | ZNRF3 C22orf31 |
| 22 | 29456699 | 29612508 | 52 | 34 | 0.029208899 | KREMEN1 EMID1 C22orf31 |
| 22 | 29613441 | 29743779 | 53 | 34 | 0.021353508 | GAS2L1 EWSR1 AP1B1 EMID1 RASL10A RHBDD3 SNORD125 |
| 22 | 29746253 | 30060389 | 52 | 34 | 0.029208899 | NF2 RFPL1 NIPSNAP1 AP1B1 NEFH RFPL1S THOC5 |
| 22 | 30652447 | 30953295 | 51 | 32 | 0.019551431 | SEC14L3 SF3A1 GATSL3 SEC14L4 SEC14L6 TBC1D10A GAL3ST1 SEC14L2 MTFP1 SDC4P OSM RNF215 CCDC157 KIAA1656 |
| 22 | 30957832 | 31250664 | 52 | 31 | 0.009427431 | TCN2 DUSP18 OSBP2 PES1 GAL3ST1 SLC35E4 |
| 22 | 31253230 | 31533967 | 51 | 33 | 0.028072356 | INPP5J SMTN PLA2G3 MORC2 OSBP2 TUG1 SELM |
| 22 | 31536133 | 32358588 | 54 | 33 | 0.010494815 | DEPDC5 PRR14L RNF185 LIMK2 C22orf24 PIK3IP1 SFI1 EIF4ENIF1 PLA2G3 PATZ1 PISD YWHAH DRG1 |
| 22 | 32365973 | 32656022 | 54 | 34 | 0.015430585 | RFPL2 SLC5A4 C22orf42 SLC5A1 |
| 22 | 32657104 | 32710985 | 51 | 33 | 0.028072356 |  |
| 22 | 32828224 | 32857285 | 52 | 32 | 0.014037891 | BPIFC |
| 22 | 32857824 | 32915743 | 52 | 33 | 0.020459321 | BPIFC SYN3 FBXO7 |
| 22 | 33513085 | 33697034 | 54 | 36 | 0.031411422 | LARGE |
| 22 | 33755061 | 33847076 | 55 | 37 | 0.032474033 | LARGE |

**Table H. Regions along chromosomes identified in RFMix analysis in the OCG above the 99% confidence interval for the difference in African ancestry.**

| Chr | Block start | Block end | No. of African blocks in asymptomatic control | No. of African blocks in fever and haemorrhagic | p-value | Protein coding genes |
| --- | --- | --- | --- | --- | --- | --- |
| 1 | 211798430 | 212093158 | 69 | 48 | 0.030948857 | NEK2 LPGAT1 |
| 1 | 242010116 | 242077448 | 67 | 46 | 0.029068241 | EXO1 |
| 1 | 242660224 | 242698177 | 71 | 49 | 0.024597111 | PLD5 |
| 2 | 36895027 | 36955005 | 67 | 46 | 0.029068241 | VIT |
| 2 | 37009282 | 37281063 | 66 | 45 | 0.028115888 | HEATR5B VIT STRN |
| 2 | 38025151 | 38027770 | 69 | 48 | 0.030948857 |  |
| 2 | 38029427 | 38126963 | 69 | 48 | 0.030948857 |  |
| 2 | 38131976 | 38491993 | 72 | 46 | 0.007089807 | CYP1B1 RMDN2 |
| 2 | 38492587 | 38654657 | 72 | 46 | 0.007089807 | ATL2 |
| 2 | 38656353 | 38865867 | 72 | 48 | 0.013788851 | HNRNPLL |
| 2 | 38871161 | 38956947 | 75 | 49 | 0.008038756 | GALM |
| 2 | 38960491 | 39748858 | 74 | 47 | 0.005462351 | MAP4K3 GEMIN6 ARHGEF33 GALM MORN2 SRSF7 DHX57 SOS1 CDKL4 |
| 2 | 39751029 | 40009531 | 73 | 48 | 0.010360769 | TMEM178A THUMPD2 |
| 2 | 40014232 | 40279616 | 70 | 47 | 0.017574821 |  |
| 2 | 40485074 | 40558573 | 68 | 47 | 0.030012842 |  |
| 2 | 40560630 | 40626855 | 67 | 46 | 0.029068241 |  |
| 7 | 155788982 | 155844112 | 74 | 52 | 0.026847843 |  |
| 7 | 155844449 | 155920358 | 77 | 51 | 0.008671413 |  |
| 7 | 155921264 | 155967382 | 78 | 49 | 0.003192102 |  |
| 7 | 155968611 | 156037378 | 79 | 48 | 0.001573862 |  |
| 7 | 156039150 | 156042560 | 78 | 48 | 0.002198222 |  |
| 7 | 156043710 | 156107390 | 79 | 51 | 0.004762144 |  |
| 7 | 156112148 | 156232935 | 75 | 50 | 0.011162494 |  |
| 7 | 156236883 | 156268109 | 73 | 49 | 0.014292006 |  |
| 7 | 156271582 | 156347684 | 72 | 50 | 0.025355034 |  |
| 7 | 157631868 | 157650469 | 73 | 52 | 0.034592061 |  |
| 7 | 157653065 | 157744289 | 76 | 50 | 0.008355373 |  |
| 7 | 157744752 | 157784649 | 75 | 50 | 0.011162494 |  |
| 7 | 157785543 | 157873975 | 73 | 49 | 0.014292006 |  |
| 7 | 157875083 | 158028901 | 74 | 49 | 0.010762154 |  |
| 7 | 158031109 | 158064729 | 75 | 48 | 0.005708912 |  |
| 7 | 158069264 | 158176618 | 79 | 50 | 0.003339217 |  |
| 7 | 158178405 | 158536345 | 80 | 51 | 0.003486982 | PTPRN2 NCAPG2 ESYT2 |
| 7 | 158543734 | 158759602 | 80 | 52 | 0.004954641 | ESYT2 WDR60 |
| 7 | 158763288 | 158933239 | 80 | 51 | 0.003486982 | VIPR2 |
| 7 | 158940121 | 159124481 | 80 | 51 | 0.003486982 |  |
| 8 | 70374961 | 70450071 | 63 | 42 | 0.025221428 | SULF1 |
| 8 | 70451302 | 70478804 | 62 | 41 | 0.02424746 |  |
| 8 | 70617568 | 70744812 | 64 | 43 | 0.026191515 |  |
| 8 | 72015209 | 72132939 | 66 | 45 | 0.028115888 | EYA1 |
| 9 | 85592943 | 85800943 | 71 | 50 | 0.03279204 | RASEF |
| 9 | 86184504 | 86669426 | 75 | 54 | 0.036343855 | KIF27 C9orf64 HNRNPK UBQLN1 RMI1 GKAP1 IDNK |
| 9 | 86676854 | 86716976 | 76 | 54 | 0.028305839 |  |
| 9 | 86718749 | 86771998 | 77 | 55 | 0.029020368 |  |
| 9 | 109063830 | 109205296 | 64 | 43 | 0.026191515 |  |
| 9 | 109210458 | 109296536 | 64 | 43 | 0.026191515 |  |
| 11 | 36472800 | 36864766 | 60 | 39 | 0.022292404 | C11orf74 PRR5L TRAF6 RAG1 RAG2 |
| 14 | 23317223 | 23525653 | 72 | 50 | 0.025355034 | PRMT5 HAUS4 AJUBA PSMB5 LRP10 CDH24 RBM23 C14orf93 REM2 PSMB11 |
| 14 | 24114490 | 24159882 | 67 | 46 | 0.029068241 | DHRS2 |
| 18 | 5203885 | 5313164 | 66 | 45 | 0.028115888 | ZBTB14 |
| 18 | 5316070 | 5346862 | 65 | 44 | 0.027156667 |  |
| 18 | 5436263 | 5563859 | 67 | 46 | 0.029068241 | EPB41L3 |
| 18 | 5564481 | 5615156 | 69 | 45 | 0.01227117 |  |
| 18 | 5615842 | 5742915 | 68 | 46 | 0.022285484 | EPB41L3 |
| 18 | 5744872 | 5836382 | 69 | 46 | 0.016944517 |  |
| 18 | 5840255 | 5932869 | 68 | 46 | 0.022285484 | TMEM200C |
| 18 | 5937732 | 5990570 | 68 | 46 | 0.022285484 | L3MBTL4 |
| 18 | 6024678 | 6069747 | 70 | 47 | 0.017574821 |  |
| 18 | 6237821 | 6386658 | 67 | 46 | 0.029068241 |  |
| 18 | 6388022 | 6429648 | 68 | 46 | 0.022285484 | L3MBTL4 |
| 18 | 6433650 | 6484816 | 67 | 46 | 0.029068241 |  |
| 18 | 7105988 | 7132587 | 69 | 47 | 0.023061605 | LAMA1 |
| 19 | 31143402 | 31323090 | 65 | 44 | 0.027156667 |  |
| 21 | 10873592 | 15024549 | 68 | 47 | 0.030012842 | BAGE4 BAGE3 BAGE2 BAGE TPTE BAGE5 ANKRD30BP2 POTED |
| 21 | 15170179 | 15244894 | 69 | 47 | 0.023061605 | C21orf15 |
| 21 | 15270083 | 15448820 | 70 | 46 | 0.012777804 | ANKRD20A11P |
| 21 | 15449991 | 15580827 | 70 | 45 | 0.009155606 | LIPI |
| 21 | 15588213 | 15726002 | 68 | 45 | 0.016312034 | RBM11 ABCC13 |
| 21 | 15729517 | 15853435 | 70 | 46 | 0.012777804 | HSPA13 |
| 21 | 15855430 | 15906590 | 71 | 47 | 0.013283912 | SAMSN1 |
| 21 | 15909251 | 15959473 | 72 | 46 | 0.007089807 | SAMSN1 |
| 21 | 15961371 | 16131349 | 68 | 45 | 0.016312034 |  |
| 21 | 16201773 | 16216399 | 66 | 45 | 0.028115888 |  |
| 21 | 16219585 | 16298857 | 70 | 46 | 0.012777804 |  |
| 21 | 16315838 | 16451439 | 71 | 48 | 0.018202224 | NRIP1 |
| 21 | 16451845 | 16499656 | 69 | 48 | 0.030948857 |  |
| 22 | 23933452 | 23998474 | 64 | 41 | 0.013775922 | DRICH1 GUSBP11 |
| 22 | 24184850 | 24220160 | 65 | 41 | 0.010253011 | SLC2A11 |
| 22 | 24220358 | 24340940 | 65 | 43 | 0.01993327 | GSTTP1 GSTT2 SLC2A11 MIF DDTL DDT GSTT2B |
| 22 | 24400360 | 24612115 | 63 | 42 | 0.025221428 | CABIN1 SUSD2 GSTTP2 |
| 22 | 24615506 | 24977286 | 63 | 41 | 0.018354125 | SPECC1L SNRPD3 ADORA2A UPB1 GGT5 GUCD1 POM121L9P |
| 22 | 24983110 | 25131985 | 61 | 38 | 0.011891336 | LRRC75B PIWIL3 GGT1 BCRP3 POM121L10P |
| 22 | 25832419 | 25931606 | 65 | 44 | 0.027156667 | CRYBB2P1 |
| 22 | 25934878 | 26149462 | 69 | 47 | 0.023061605 | ADRBK2 MYO18B |
| 22 | 26150584 | 26223107 | 67 | 46 | 0.029068241 |  |
| 22 | 26463024 | 26521071 | 67 | 45 | 0.021504826 |  |
| 22 | 26528017 | 26691719 | 67 | 45 | 0.021504826 | SEZ6L |
| 22 | 26691965 | 26737148 | 67 | 44 | 0.015678127 | SEZ6L |
| 22 | 26737544 | 26787309 | 70 | 44 | 0.006462491 | SEZ6L |
| 22 | 26790999 | 26818675 | 71 | 44 | 0.004731202 |  |
| 22 | 26821469 | 26974741 | 71 | 43 | 0.003252799 | HPS4 ASPHD2 TFIP11 SRRD TPST2 |
| 22 | 26975517 | 27149768 | 71 | 41 | 0.001464716 | MIAT TPST2 CRYBB1 CRYBA4 |
| 22 | 27152964 | 27158663 | 70 | 41 | 0.002067654 |  |
| 22 | 27159832 | 27192401 | 71 | 41 | 0.001464716 |  |
| 22 | 27193844 | 27208422 | 75 | 46 | 0.002757063 |  |
| 22 | 27210691 | 27292868 | 75 | 44 | 0.001253378 |  |
| 22 | 27295234 | 27325416 | 71 | 47 | 0.013283912 |  |
| 22 | 27326221 | 27364610 | 71 | 48 | 0.018202224 |  |
| 22 | 27367952 | 27425790 | 70 | 49 | 0.031875502 |  |
| 22 | 27773685 | 27805463 | 69 | 48 | 0.030948857 |  |
| 22 | 28174632 | 28191895 | 70 | 47 | 0.017574821 |  |
| 22 | 28198577 | 29456360 | 71 | 48 | 0.018202224 | PITPNB CHEK2 ZNRF3 XBP1 CCDC117 TTC28 C22orf31 HSCB |
| 22 | 29456699 | 29612508 | 72 | 48 | 0.013788851 | KREMEN1 EMID1 C22orf31 |
| 22 | 29613441 | 29743779 | 72 | 48 | 0.013788851 | GAS2L1 EWSR1 AP1B1 EMID1 RASL10A RHBDD3 SNORD125 |
| 22 | 29746253 | 30060389 | 70 | 48 | 0.023832397 | NF2 RFPL1 NIPSNAP1 AP1B1 NEFH RFPL1S THOC5 |
| 22 | 30652447 | 30953295 | 69 | 47 | 0.023061605 | SEC14L3 SF3A1 GATSL3 SEC14L4 SEC14L6 TBC1D10A GAL3ST1 SEC14L2 MTFP1 SDC4P OSM RNF215 CCDC157 KIAA1656 |
| 22 | 30957832 | 31250664 | 70 | 46 | 0.012777804 | TCN2 DUSP18 OSBP2 PES1 GAL3ST1 SLC35E4 |
| 22 | 31536133 | 32358588 | 72 | 48 | 0.013788851 | DEPDC5 PRR14L RNF185 LIMK2 C22orf24 PIK3IP1 SFI1 EIF4ENIF1 PLA2G3 PATZ1 PISD YWHAH DRG1 |
| 22 | 32365973 | 32656022 | 72 | 49 | 0.018826034 | RFPL2 SLC5A4 C22orf42 AP1B1P1 SLC5A1 |
| 22 | 32828224 | 32857285 | 70 | 49 | 0.031875502 | BPIFC |

Two of the regions displaying a significantly higher proportion of African ancestry in the asymptomatic/control subjects overlap in DHF and DF comparison groups (HCG and FCG). One is the region on chromosome 9, containing the *RXRA* (retinoid X receptor alpha) gene referred in the main text. The other overlapping region is on chromosome 7, containing the *PTPRN2* (protein tyrosine phosphatase, receptor type, N polypeptide 2), *NCAPG2* (non-SMC condensin II complex, subunit G2), *ESYT2* (extended synaptotagmin-like protein 2), *WDR60* (WD repeat domain 60) and *VIPR2* (vasoactive intestinal peptide receptor 2) genes. Of these, *PTPRN2*, *NCAPG2* and *ESYT2* have significantly differentiated expressions in the Thai dengue dataset [[6](#_ENREF_6)]. *PTPRN2* is an interesting finding as it may dephosphorylate PI3P [[7](#_ENREF_7)], but it is surpassed in terms of the top significantly associated gene in this region by *VIPR2* (Table K), which is not differently expressed in Thai dengue patients. This gene was also detected as being under selection in the DHF group in the XP-EHH analysis (Table N), and encodes a receptor for the small neuropeptide vasoactive intestinal peptide, involved in water and ion flux.

In the overall comparison group (OCG), this region 7 is also detected (Table H), as well as a long region in chromosome 22 overlapping with the FCG results (Table G). Interestingly, the *OSBP2* gene is in this region, being up-regulated in controls/convalescents (Fig L), while the chromosome 3 located *OSBPL10* is up-regulated in DHF. Another interesting gene in chromosome 22 is *SEC14L2* (and other family members), which has recently been shown to be essential for hepatitis C virus (HCV) replication in cell culture [[8](#_ENREF_8)]. No differential expression for the *SEC14L2* gene was reported in the Thai dataset. Nevertheless, the top p-values (Table L) in this region are for genes *SYN3* (synapsin III; one SNP detected also in the association test in FCG) and *TTC28* (tetratricopeptide repeat domain 28), with no evident link to viral diseases in the literature so far.


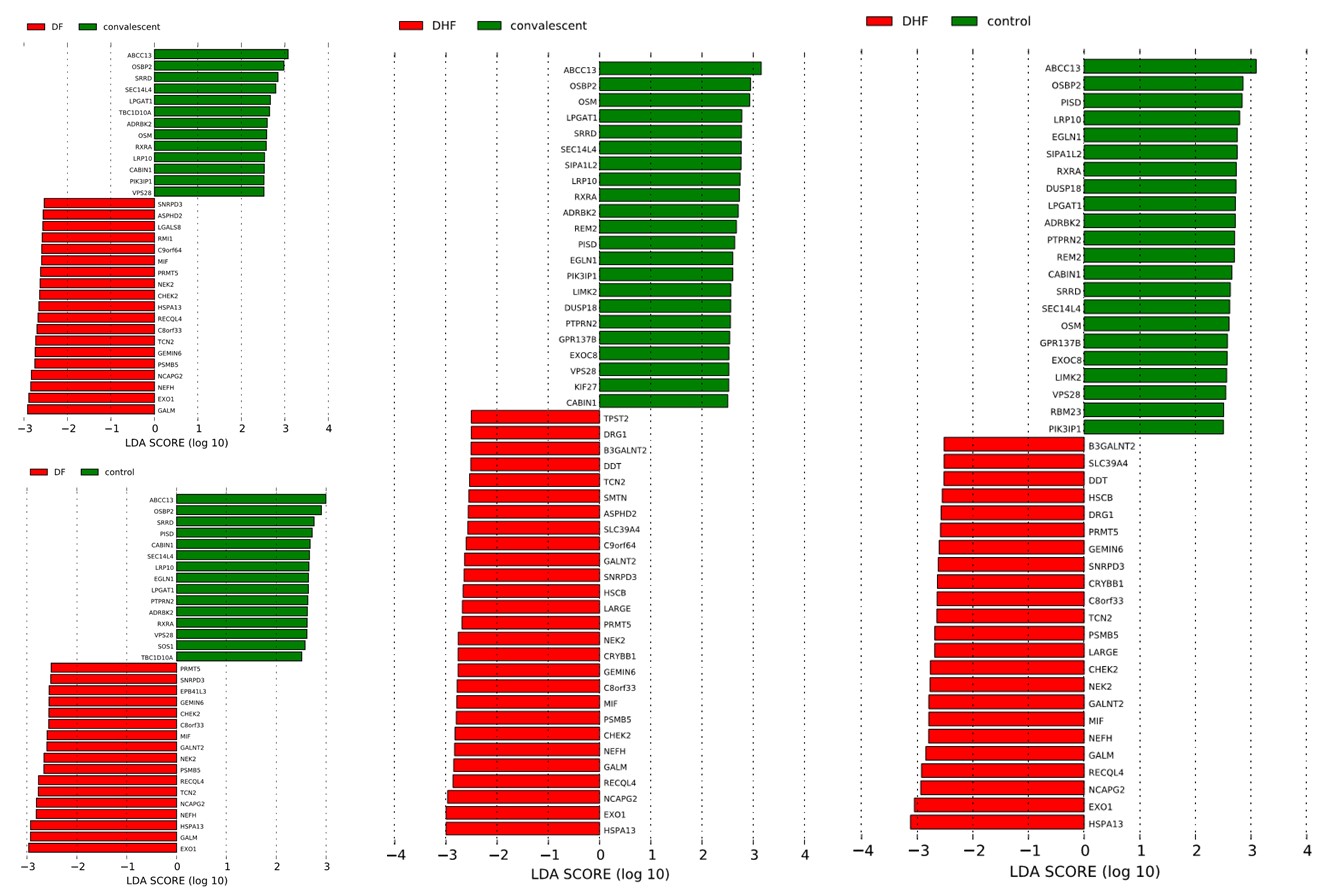


**Fig 12. LefSe for African-related genes detected in RFMix with dengue fever in the three comparison groups (HCG, FCG and OCG).**

*1.6- P-values of specific regions*

The following tables report p-values in the HCG in specific regions discussed in the main text and here.

**Table I. Significant p-values (5% level) of the association test in the HCG for *OSBPL10* gene region.** In blue the haplotype region. MAF in the 108 individuals higher or equal to 5%.

| Chr | ID in the chip | ID in Ensembl | Position in GRCh37 | Allele | Frequency in hemorrhagic | Frequency in asymptomatic control | Other allele | Pval | Pval-log(10) | OR | OR alternative allele |
| --- | --- | --- | --- | --- | --- | --- | --- | --- | --- | --- | --- |
| 3 | kgp3944551 | rs11129475 | 32030544 | T | 0.2037 | 0.5463 | C | 0.0000001991 | 6.70092873997 | 0.2125 |  |
| 3 | rs6419811 | rs6419811 | 32031135 | G | 0.2685 | 0.5833 | A | 0.000002891 | 5.53895190833 | 0.2622 |  |
| 3 | rs11718700 | rs11718700 | 32033248 | C | 0.2685 | 0.5741 | A | 0.000005431 | 5.26512019721 | 0.2724 |  |
| 3 | kgp2595741 | rs975406 | 32035587 | C | 0.2685 | 0.5741 | T | 0.000005431 | 5.26512019721 | 0.2724 |  |
| 3 | kgp8630333 | rs7639637 | 32036042 | C | 0.2685 | 0.5741 | A | 0.000005431 | 5.26512019721 | 0.2724 |  |
| 3 | kgp3657536 | rs4600849 | 32027672 | C | 0.3113 | 0.6111 | T | 0.00001094 | 4.960982678 | 0.2877 |  |
| 3 | kgp3235019 | rs35742300 | 31848340 | A | 0.2685 | 0.07407 | C | 0.0001492 | 3.82623117686 | 4.589 | 0.2179 |
| 3 | rs9853939 | rs9853939 | 31989487 | A | 0.5 | 0.2593 | C | 0.000267 | 3.57348873864 | 2.857 | 0.3500 |
| 3 | kgp18227612 | rs35313927 | 32012295 | C | 0.3056 | 0.5463 | T | 0.0003467 | 3.46004615834 | 0.3654 |  |
| 3 | kgp2422896 | rs9826785 | 32012257 | C | 0.3077 | 0.5463 | T | 0.0004501 | 3.34669098706 | 0.3691 |  |
| 3 | rs7634861 | rs7634861 | 32016209 | T | 0.3019 | 0.537 | G | 0.0004939 | 3.30636097384 | 0.3728 |  |
| 3 | rs6773772 | rs6773772 | 31851726 | C | 0.1759 | 0.3704 | T | 0.001342 | 2.87224748417 | 0.3629 |  |
| 3 | kgp7572224 | rs13070370 | 32059168 | C | 0.5 | 0.287 | T | 0.001358 | 2.86710023006 | 2.484 | 0.4026 |
| 3 | rs9847800 | rs9847800 | 32001042 | C | 0.4537 | 0.25 | A | 0.001721 | 2.76421912967 | 2.492 | 0.4013 |
| 3 | rs4073766 | rs4073766 | 31988094 | C | 0.213 | 0.4074 | T | 0.002008 | 2.69723629153 | 0.3936 |  |
| 3 | kgp707171 | rs9824465 | 31979027 | A | 0.4907 | 0.2963 | C | 0.003446 | 2.46268472689 | 2.289 | 0.4369 |
| 3 | rs6800757 | rs6800757 | 31980606 | T | 0.4907 | 0.2963 | G | 0.003446 | 2.46268472689 | 2.289 | 0.4369 |
| 3 | kgp129940 | rs6772029 | 31970860 | G | 0.4906 | 0.2963 | A | 0.003616 | 2.4417715782 | 2.287 | 0.4373 |
| 3 | kgp12172992 | rs9816870 | 31989813 | C | 0.5556 | 0.3611 | T | 0.004135 | 2.38352448611 | 2.212 | 0.4521 |
| 3 | rs11719930 | rs11719930 | 32011584 | T | 0.1019 | 0.25 | C | 0.004247 | 2.37191773901 | 0.3402 |  |
| 3 | kgp5272087 | rs4521276 | 32023014 | A | 0.1667 | 0.3333 | G | 0.004678 | 2.32993978253 | 0.4 |  |
| 3 | rs6806060 | rs6806060 | 32033850 | G | 0.03704 | 0.1481 | A | 0.00485 | 2.3142582614 | 0.2212 |  |
| 3 | kgp10892024 | rs35158456 | 31848096 | T | 0.2593 | 0.1111 | C | 0.005069 | 2.29507770878 | 2.8 | 0.3571 |
| 3 | kgp5787221 | rs11920907 | 31974753 | A | 0.1132 | 0.2593 | G | 0.006141 | 2.21176090262 | 0.3647 |  |
| 3 | rs11916514 | rs11916514 | 32002469 | C | 0.1574 | 0.3148 | A | 0.006457 | 2.18996921359 | 0.4066 |  |
| 3 | kgp4451931 | rs74550005 | 32030111 | C | 0.1019 | 0.2407 | T | 0.006751 | 2.17063189201 | 0.3577 |  |
| 3 | kgp3664427 | rs4645161 | 31977744 | T | 0.2925 | 0.4717 | A | 0.00724 | 2.1402614338 | 0.4629 |  |
| 3 | kgp4967303 | rs76132765 | 31796150 | G | 0.06481 | 0.1852 | A | 0.007482 | 2.12598229614 | 0.305 |  |
| 3 | kgp5655288 | rs72852994 | 32002714 | A | 0.06481 | 0.1852 | G | 0.007482 | 2.12598229614 | 0.305 |  |
| 3 | kgp10451916 | rs58739627 | 31958368 | A | 0.25 | 0.4167 | G | 0.009375 | 2.0280287236 | 0.4667 |  |
| 3 | kgp12544555 | rs61466561 | 31958989 | G | 0.25 | 0.4167 | A | 0.009375 | 2.0280287236 | 0.4667 |  |
| 3 | kgp4444858 | rs61289065 | 31959100 | C | 0.25 | 0.4167 | A | 0.009375 | 2.0280287236 | 0.4667 |  |
| 3 | rs1597666 | rs1597666 | 32064607 | T | 0.3519 | 0.5185 | C | 0.0135 | 1.8696662315 | 0.5041 |  |
| 3 | rs4334675 | rs4334675 | 31933619 | A | 0.07407 | 0.1852 | G | 0.01507 | 1.82188674769 | 0.352 |  |
| 3 | kgp445975 | rs11129474 | 31991741 | A | 0.2037 | 0.3519 | C | 0.01507 | 1.82188674769 | 0.4712 |  |
| 3 | kgp793893 | rs73824712 | 31936992 | A | 0.05556 | 0.1574 | G | 0.01525 | 1.81673015632 | 0.3149 |  |
| 3 | rs13317583 | rs13317583 | 31951420 | A | 0.1296 | 0.2593 | G | 0.01609 | 1.7934439559 | 0.4255 |  |
| 3 | kgp8522254 | rs7609659 | 31992506 | T | 0.1759 | 0.3148 | C | 0.0177 | 1.75202673364 | 0.4646 |  |
| 3 | kgp4328171 | rs75240811 | 31787238 | T | 0.0463 | 0.1389 | C | 0.01891 | 1.72330847115 | 0.301 |  |
| 3 | rs6798458 | rs6798458 | 31831109 | G | 0.3611 | 0.5185 | A | 0.01979 | 1.70355420579 | 0.5248 |  |
| 3 | rs11916062 | rs11916062 | 31989920 | A | 0.1481 | 0.2778 | G | 0.01998 | 1.69940451611 | 0.4522 |  |
| 3 | kgp8981594 | rs11716431 | 32014104 | T | 0.1759 | 0.3056 | C | 0.02587 | 1.58720357128 | 0.4852 |  |
| 3 | rs4383559 | rs4383559 | 32019685 | C | 0.217 | 0.3519 | T | 0.02888 | 1.5394028111 | 0.5105 |  |
| 3 | rs11925027 | rs11925027 | 31978325 | A | 0.1481 | 0.2685 | C | 0.0294 | 1.53165266959 | 0.4738 |  |
| 3 | kgp5821792 | rs13086512 | 31867937 | T | 0.2963 | 0.4352 | C | 0.03408 | 1.46750041391 | 0.5465 |  |
| 3 | rs12489744 | rs12489744 | 32003769 | T | 0.1481 | 0.2642 | C | 0.03578 | 1.44635966377 | 0.4845 |  |
| 3 | kgp3791873 | rs9637510 | 31966929 | C | 0.5189 | 0.375 | G | 0.03606 | 1.44297427761 | 1.797 | 0.5565 |
| 3 | kgp10877154 | rs34941285 | 31890716 | G | 0.463 | 0.3241 | A | 0.03669 | 1.43545228824 | 1.798 | 0.5562 |
| 3 | rs6769296 | rs6769296 | 32017806 | G | 0.05556 | 0.1389 | A | 0.03873 | 1.41195250301 | 0.3647 |  |
| 3 | kgp1115889 | rs62243012 | 31897366 | A | 0.1389 | 0.25 | G | 0.03911 | 1.40771218405 | 0.4839 |  |
| 3 | rs11921166 | rs11921166 | 31999834 | T | 0.1574 | 0.2685 | C | 0.04611 | 1.33620487778 | 0.5089 |  |
| 3 | kgp3592840 | rs80170080 | 31829979 | C | 0.06481 | 0.1481 | T | 0.04711 | 1.32688689576 | 0.3985 |  |
| 3 | kgp2308058 | rs6550093 | 32051193 | T | 0.0463 | 0.1204 | C | 0.0489 | 1.31069114088 | 0.3547 |  |

**Table J. Significant p-values (5% level) of the association test in the *RXRA*-*COL5A1* region in HCG.**

| Chr | ID in the chip | ID in Ensembl | Position in GRCh37 | Allele | Frequency in hemorrhagic | Frequency in asymptomatic  control | Other allele | Pval | Pval-log(10) | OR | Location |
| --- | --- | --- | --- | --- | --- | --- | --- | --- | --- | --- | --- |
| 9 | kgp6266655 | rs4262378 | 137515156 | G | 0.3241 | 0.537 | A | 0.001575 | 2.802719442 | 0.4133 | intergenic RXRA-COL5A1 |
| 9 | rs4424343 | rs4424343 | 137515158 | A | 0.2778 | 0.4722 | G | 0.003163 | 2.499900808 | 0.4299 | intergenic RXRA-COL5A1 |
| 9 | rs3118593 | rs3118593 | 137426334 | A | 0.3113 | 0.5093 | C | 0.003259 | 2.48691564 | 0.4356 | intergenic RXRA-COL5A1; lnc-RXRA-3:5 or RP11-473E2.4 |
| 9 | kgp7779586 | rs7036416 | 137723551 | T | 0.2222 | 0.4074 | C | 0.003389 | 2.469928431 | 0.4156 | COL5A1 |
| 9 | kgp1560973 | rs9409996 | 137717379 | C | 0.5833 | 0.3889 | A | 0.004252 | 2.371406744 | 2.2 | COL5A1 |
| 9 | rs7044529 | rs7044529 | 137568051 | T | 0.1296 | 0.287 | C | 0.004397 | 2.356843534 | 0.3699 | COL5A1 |
| 9 | rs11103544 | rs11103544 | 137735043 | C | 0.1111 | 0.2593 | T | 0.005069 | 2.295077709 | 0.3571 | COL5A1 |
| 9 | kgp11204176 | rs9409921 | 137717224 | T | 0.3981 | 0.2222 | G | 0.005193 | 2.284581677 | 2.315 | COL5A1 |
| 9 | rs4842173 | rs4842173 | 137718113 | C | 0.3981 | 0.2222 | T | 0.005193 | 2.284581677 | 2.315 | COL5A1 |
| 9 | kgp2730115 | rs4072790 | 137590710 | A | 0.5288 | 0.3396 | G | 0.005663 | 2.246953438 | 2.183 | COL5A1 |
| 9 | kgp4309653 | rs72772548 | 137582724 | T | 0.1759 | 0.05556 | C | 0.005694 | 2.244582537 | 3.629 | COL5A1 |
| 9 | rs12339163 | rs12339163 | 137205188 | G | 0.1019 | 0.2407 | A | 0.006751 | 2.170631892 | 0.3577 | Before RXRA |
| 9 | rs6537942 | rs6537942 | 137559556 | G | 0.1481 | 0.3019 | A | 0.007023 | 2.153477332 | 0.4022 | COL5A1 |
| 9 | rs3109677 | rs3109677 | 137630568 | G | 0.5556 | 0.3796 | A | 0.009569 | 2.019133445 | 2.043 | COL5A1 |
| 9 | kgp18666579 | rs62576287 | 137214888 | C | 0.009259 | 0.08333 | A | 0.009584 | 2.018453195 | 0.1028 | RXRA |
| 9 | kgp7101445 | rs114989133 | 137358930 | G | 0 | 0.05556 | T | 0.01384 | 1.85886391 | 0 | intergenic RXRA-COL5A1 |
| 9 | kgp18401799 | rs76917123 | 137417212 | A | 0 | 0.05556 | G | 0.01384 | 1.85886391 | 0 | intergenic RXRA-COL5A1 |
| 9 | kgp11177565 | rs9308278 | 137717146 | G | 0.4167 | 0.2593 | A | 0.01447 | 1.839531469 | 2.041 | COL5A1 |
| 9 | kgp3057400 | rs11103439 | 137520289 | A | 0.05556 | 0.1574 | C | 0.01525 | 1.816730156 | 0.3149 | lnc-COL5A1-1:3 |
| 9 | kgp8290346 | rs10858272 | 137592737 | T | 0.0283 | 0.1132 | C | 0.01592 | 1.798056937 | 0.2282 | COL5A1 |
| 9 | rs3118546 | rs3118546 | 137351129 | T | 0.07407 | 0.009259 | C | 0.01715 | 1.765735876 | 8.56 | intergenic RXRA-COL5A1 |
| 9 | kgp1859411 | rs45443491 | 137716422 | T | 0.07407 | 0.009259 | C | 0.01715 | 1.765735876 | 8.56 | COL5A1 |
| 9 | kgp598926 | rs56377304 | 137578387 | T | 0.09259 | 0.01852 | C | 0.01748 | 1.757458572 | 5.408 | COL5A1 |
| 9 | kgp10287409 | rs3109673 | 137644905 | A | 0.5093 | 0.3519 | G | 0.01949 | 1.710188161 | 1.912 | COL5A1 |
| 9 | kgp10791741 | rs7041099 | 137629831 | T | 0.5278 | 0.3704 | C | 0.02005 | 1.697885623 | 1.9 | COL5A1 |
| 9 | kgp5430913 | rs3132331 | 137394085 | A | 0.5189 | 0.3611 | C | 0.02008 | 1.697236292 | 1.908 | intergenic RXRA-COL5A1 |
| 9 | rs3118520 | rs3118520 | 137441595 | G | 0.4074 | 0.5648 | A | 0.02065 | 1.685079944 | 0.5297 | intergenic RXRA-COL5A1 |
| 9 | rs3128615 | rs3128615 | 137641411 | G | 0.4167 | 0.2685 | A | 0.02179 | 1.66174277 | 1.946 | COL5A1 |
| 9 | kgp10611233 | rs76009850 | 137387756 | G | 0 | 0.0463 | A | 0.02367 | 1.625801742 | 0 | intergenic RXRA-COL5A1 |
| 9 | rs11103276 | rs11103276 | 137447293 | G | 0.2315 | 0.3704 | A | 0.02607 | 1.583858969 | 0.512 | intergenic RXRA-COL5A1 |
| 9 | kgp4449666 | rs7031092 | 137727859 | G | 0.07547 | 0.1759 | T | 0.02691 | 1.570086302 | 0.3824 | COL5A1 |
| 9 | kgp9852808 | rs7870784 | 137730577 | C | 0.1111 | 0.2222 | T | 0.02846 | 1.545765104 | 0.4375 | COL5A1 |
| 9 | rs12554098 | rs12554098 | 137573609 | A | 0.0463 | 0.1296 | G | 0.03062 | 1.513994814 | 0.3259 | COL5A1 |
| 9 | kgp9813057 | rs76482208 | 137583718 | T | 0.1132 | 0.2222 | C | 0.03304 | 1.480959961 | 0.4468 | COL5A1 |
| 9 | kgp10334323 | rs72774465 | 137688863 | A | 0.3269 | 0.4712 | G | 0.03368 | 1.472627917 | 0.5452 | COL5A1 |
| 9 | kgp4432921 | rs7850942 | 137723224 | T | 0.3585 | 0.5 | C | 0.03656 | 1.436993813 | 0.5588 | COL5A1 |
| 9 | rs4504708 | rs4504708 | 137736544 | G | 0.2963 | 0.1759 | T | 0.03727 | 1.428640607 | 1.972 | COL5A1 |
| 9 | rs3128574 | rs3128574 | 137736913 | A | 0.2963 | 0.1759 | G | 0.03727 | 1.428640607 | 1.972 | After COL5A1 |
| 9 | kgp2256907 | rs80116549 | 137483068 | A | 0.03704 | 0.1111 | G | 0.03767 | 1.42400438 | 0.3077 | intergenic RXRA-COL5A1 |
| 9 | kgp10764509 | rs7357805 | 137713556 | T | 0.4815 | 0.3426 | C | 0.03812 | 1.418847108 | 1.782 | COL5A1 |
| 9 | kgp655589 | rs12338780 | 137584429 | T | 0.3491 | 0.2222 | C | 0.03989 | 1.399135964 | 1.877 | COL5A1 |
| 9 | rs6537998 | rs6537998 | 137292505 | C | 0.1321 | 0.2407 | T | 0.04148 | 1.382161252 | 0.4799 | RXRA |
| 9 | kgp7779428 | rs117846223 | 137629431 | A | 0 | 0.03774 | G | 0.04156 | 1.381324461 | 0 | COL5A1 |
| 9 | kgp4007279 | rs116203323 | 137382118 | A | 0 | 0.03704 | G | 0.04351 | 1.361410917 | 0 | intergenic RXRA-COL5A1 |
| 9 | kgp18307138 | rs7028646 | 137411225 | A | 0 | 0.03704 | C | 0.04351 | 1.361410917 | 0 | lnc-C9orf104-2:1 AND lnc-RXRA-3:4 |
| 9 | kgp18368068 | rs116771553 | 137661097 | G | 0.03704 | 0 | A | 0.04351 | 1.361410917 | NA | COL5A1 |
| 9 | rs4841934 | rs4841934 | 137707942 | T | 0.4074 | 0.2778 | C | 0.04473 | 1.349401102 | 1.788 | COL5A1 |
| 9 | kgp18640954 | rs34373156 | 137333067 | T | 0 | 0.03704 | G | 0.04548 | 1.342179544 | 0 | intergenic RXRA-COL5A1 |
| 9 | rs7865626 | rs7865626 | 137518733 | A | 0.2778 | 0.1667 | C | 0.04953 | 1.305131672 | 1.923 | intergenic RXRA-COL5A1 |
| 9 | rs3922914 | rs3922914 | 137598731 | G | 0.1509 | 0.2593 | A | 0.04997 | 1.301290651 | 0.5079 | COL5A1 |

**Table K. Significant p-values (5% level) of the association test in the African enriched chromosome 7 region in FCG.** MAF>5% in all individuals in the comparison group.

| Chr | ID chip | ID ensembl | Position in GRCh37 | A1 | Frequency in fever | Frequency in asymptomatic  control | A2 | P-value | OR | Location |
| --- | --- | --- | --- | --- | --- | --- | --- | --- | --- | --- |
| 7 | kgp11171420 | rs917992 | 158881399 | G | 0.287 | 0.5 | A | 0.001358 | 0.4026 | VIPR2 |
| 7 | kgp10376489 | rs3828964 | 158921112 | C | 0.09259 | 0.25 | T | 0.00214 | 0.3061 | VIPR2 |
| 7 | rs3793239 | rs3793239 | 158917999 | G | 0.3148 | 0.5185 | T | 0.002395 | 0.4266 | VIPR2 |
| 7 | kgp1351868 | rs12670064 | 158869415 | C | 0.2407 | 0.4352 | T | 0.002521 | 0.4115 | VIPR2 |
| 7 | kgp9002218 | rs67464981 | 158882703 | C | 0.25 | 0.4444 | T | 0.002689 | 0.4167 | VIPR2 |
| 7 | kgp12375789 | rs10233396 | 157512290 | T | 0.3704 | 0.5741 | C | 0.002714 | 0.4364 | PTPRN2 |
| 7 | kgp9424396 | rs6950603 | 157513940 | T | 0.3148 | 0.1481 | C | 0.003687 | 2.642 | PTPRN2 |
| 7 | rs10256772 | rs10256772 | 157514189 | A | 0.3148 | 0.1481 | G | 0.003687 | 2.642 | PTPRN2 |
| 7 | rs12668638 | rs12668638 | 158871812 | T | 0.25 | 0.4352 | C | 0.004138 | 0.4326 | VIPR2 |
| 7 | kgp12211711 | rs4289696 | 157508399 | T | 0.3981 | 0.5926 | C | 0.004265 | 0.4548 | PTPRN2 |
| 7 | rs6459805 | rs6459805 | 157510294 | C | 0.3981 | 0.5926 | T | 0.004265 | 0.4548 | PTPRN2 |
| 7 | kgp11784519 | rs12672820 | 157508940 | G | 0.2885 | 0.1296 | A | 0.004362 | 2.722 | PTPRN2 |
| 7 | rs9654699 | rs9654699 | 157512750 | G | 0.287 | 0.1296 | A | 0.004397 | 2.703 | PTPRN2 |
| 7 | rs4716793 | rs4716793 | 157649261 | C | 0.2222 | 0.08333 | T | 0.004556 | 3.143 | PTPRN2 |
| 7 | kgp11337546 | rs35311492 | 158032178 | A | 0.02778 | 0.1296 | G | 0.005444 | 0.1918 | PTPRN2 |
| 7 | kgp12348668 | rs57426645 | 157653129 | G | 0.2315 | 0.09259 | T | 0.005609 | 2.952 | PTPRN2 |
| 7 | kgp1506849 | rs73513298 | 157653953 | G | 0.2315 | 0.09259 | A | 0.005609 | 2.952 | PTPRN2 |
| 7 | kgp11427972 | rs73730148 | 158864283 | A | 0.01852 | 0.1111 | G | 0.005715 | 0.1509 | VIPR2 |
| 7 | kgp25489 | rs1362219 | 157521567 | A | 0.3056 | 0.1481 | G | 0.005745 | 2.53 | PTPRN2 |
| 7 | kgp197898 | rs10227495 | 158396348 | T | 0.2037 | 0.07407 | C | 0.005879 | 3.198 | intergenic PTPRN2-NCAPG2 |
| 7 | kgp6877683 | rs10447497 | 158862470 | T | 0.1019 | 0.2407 | C | 0.006751 | 0.3577 | VIPR2 |
| 7 | kgp7326756 | rs3757827 | 157058216 | T | 0.25 | 0.1111 | C | 0.007969 | 2.667 | UBE3C |
| 7 | rs2037905 | rs2037905 | 158972018 | C | 0.09259 | 0.2222 | T | 0.008906 | 0.3571 | After VIPR2 |
| 7 | rs399867 | rs399867 | 158910629 | A | 0.5278 | 0.3519 | G | 0.009201 | 2.059 | VIPR2 |
| 7 | kgp5653738 | rs10225098 | 157620406 | C | 0.03774 | 0.1389 | T | 0.009293 | 0.2431 | PTPRN2 |
| 7 | rs6944306 | rs6944306 | 157700614 | C | 0.5833 | 0.4074 | T | 0.009719 | 2.036 | PTPRN2 |
| 7 | kgp7514198 | rs1544598 | 158992691 | T | 0.1574 | 0.3056 | C | 0.009848 | 0.4246 | After VIPR2 |
| 7 | kgp480501 | rs3802108 | 157021066 | G | 0.3396 | 0.1852 | T | 0.01018 | 2.263 | UBE3C |
| 7 | rs1558638 | rs1558638 | 158878791 | T | 0.1019 | 0.2315 | C | 0.01059 | 0.3765 | VIPR2 |
| 7 | kgp12368540 | rs17837874 | 158896436 | A | 0.1019 | 0.2315 | G | 0.01059 | 0.3765 | VIPR2 |
| 7 | kgp8930977 | rs10271990 | 157037521 | T | 0.3148 | 0.1667 | C | 0.01088 | 2.297 | UBE3C |
| 7 | kgp11147375 | rs73730455 | 158393901 | T | 0.2037 | 0.08333 | C | 0.01164 | 2.814 | intergenic PTPRN2-NCAPG2 |
| 7 | kgp7002657 | rs10227167 | 157631868 | G | 0.1759 | 0.3241 | A | 0.01193 | 0.4453 | PTPRN2 |
| 7 | kgp10142239 | rs758927 | 157289982 | G | 0.4815 | 0.3148 | T | 0.01235 | 2.021 | intergenic DNAJB6-PTPRN2 |
| 7 | rs7784733 | rs7784733 | 157409757 | G | 0.1111 | 0.2407 | A | 0.01236 | 0.3942 | PTPRN2 |
| 7 | kgp13539119 | rs6950857 | 158910937 | A | 0.1111 | 0.2407 | G | 0.01236 | 0.3942 | VIPR2 |
| 7 | kgp7701672 | rs79703582 | 157734921 | A | 0.1604 | 0.05556 | G | 0.01331 | 3.247 | PTPRN2 |
| 7 | rs7795387 | rs7795387 | 158071511 | G | 0.1481 | 0.287 | A | 0.01338 | 0.432 | PTPRN2 |
| 7 | kgp4431732 | rs1034774 | 158754716 | T | 0.5648 | 0.3981 | C | 0.01424 | 1.962 | Intergenic WDR60-VIPR2 |
| 7 | kgp12364210 | rs7792857 | 157513676 | G | 0.3796 | 0.2264 | A | 0.01482 | 2.091 | PTPRN2 |
| 7 | rs11980462 | rs11980462 | 157289534 | T | 0.4259 | 0.2685 | C | 0.01512 | 2.021 | intergenic DNAJB6-PTPRN2 |
| 7 | kgp9283687 | rs76100699 | 157733036 | A | 0.1574 | 0.05556 | G | 0.01525 | 3.176 | PTPRN2 |
| 7 | kgp10736243 | rs73730145 | 158863345 | T | 0.01852 | 0.09259 | G | 0.01748 | 0.1849 | VIPR2 |
| 7 | kgp8242869 | rs73730153 | 158871028 | T | 0.01852 | 0.09259 | C | 0.01748 | 0.1849 | VIPR2 |
| 7 | rs11973244 | rs11973244 | 158075769 | A | 0.3426 | 0.5 | G | 0.01915 | 0.5211 | PTPRN2 |
| 7 | kgp9895086 | rs11763290 | 158057567 | A | 0.2593 | 0.1321 | G | 0.01917 | 2.3 | PTPRN2 |
| 7 | rs17837871 | rs17837871 | 158875394 | C | 0.06481 | 0.1667 | T | 0.01931 | 0.3465 | VIPR2 |
| 7 | kgp7118391 | rs73730155 | 158875670 | T | 0.06481 | 0.1667 | C | 0.01931 | 0.3465 | VIPR2 |
| 7 | kgp7873926 | rs77236243 | 158231384 | T | 0.2778 | 0.1481 | A | 0.01998 | 2.212 | PTPRN2 |
| 7 | rs4716752 | rs4716752 | 157308110 | C | 0.5556 | 0.3981 | T | 0.02056 | 1.89 | intergenic DNAJB6-PTPRN2 |
| 7 | kgp10216265 | rs12669974 | 158891838 | A | 0.1204 | 0.2407 | G | 0.02147 | 0.4316 | VIPR2 |
| 7 | kgp8901759 | rs3812311 | 158930751 | C | 0.2778 | 0.4259 | T | 0.02263 | 0.5184 | VIPR2 |
| 7 | rs17837793 | rs17837793 | 157464422 | A | 0.03704 | 0.1204 | G | 0.02296 | 0.2811 | PTPRN2 |
| 7 | kgp12537437 | rs61303676 | 157513021 | T | 0.07407 | 0.1759 | C | 0.02363 | 0.3747 | PTPRN2 |
| 7 | kgp7428720 | rs6459808 | 157521180 | C | 0.2963 | 0.1667 | T | 0.02392 | 2.105 | PTPRN2 |
| 7 | kgp2697880 | rs77925279 | 157734989 | A | 0.1481 | 0.05556 | G | 0.02447 | 2.957 | PTPRN2 |
| 7 | kgp10623764 | rs1869292 | 158175731 | T | 0.05556 | 0.1481 | C | 0.02447 | 0.3382 | PTPRN2 |
| 7 | kgp5718135 | rs1453097 | 158176618 | C | 0.05556 | 0.1481 | T | 0.02447 | 0.3382 | PTPRN2 |
| 7 | kgp11432281 | rs10282082 | 158455427 | T | 0.1481 | 0.05556 | C | 0.02447 | 2.957 | NCAPG2 |
| 7 | rs3750072 | rs3750072 | 158902741 | G | 0.2037 | 0.3396 | A | 0.02532 | 0.4974 | VIPR2 |
| 7 | rs429102 | rs429102 | 159000871 | T | 0.2593 | 0.1389 | G | 0.02675 | 2.17 | After VIPR2 |
| 7 | kgp5914796 | rs181344517 | 158028467 | A | 0.1019 | 0.02778 | T | 0.02704 | 3.969 | PTPRN2 |
| 7 | kgp10205927 | rs28716662 | 157719496 | C | 0.5185 | 0.3704 | T | 0.02846 | 1.831 | PTPRN2 |
| 7 | rs1882247 | rs1882247 | 158389294 | A | 0.2222 | 0.1111 | C | 0.02846 | 2.286 | intergenic PTPRN2-NCAPG2 |
| 7 | kgp6299172 | rs10246948 | 157598638 | A | 0.1759 | 0.3019 | G | 0.03059 | 0.4937 | PTPRN2 |
| 7 | rs10266663 | rs10266663 | 158115424 | G | 0.0463 | 0.1296 | A | 0.03062 | 0.3259 | PTPRN2 |
| 7 | kgp869251 | rs12698200 | 158070092 | C | 0.2222 | 0.3519 | T | 0.03523 | 0.5263 | PTPRN2 |
| 7 | kgp7363712 | rs1121014 | 158081769 | T | 0.3491 | 0.4907 | C | 0.0358 | 0.5565 | PTPRN2 |
| 7 | kgp8028707 | rs58459772 | 157703955 | A | 0.07407 | 0.1667 | G | 0.03652 | 0.4 | PTPRN2 |
| 7 | kgp4010743 | rs73517046 | 157713041 | C | 0.07407 | 0.1667 | A | 0.03652 | 0.4 | PTPRN2 |
| 7 | kgp2241933 | rs896767 | 158072903 | A | 0.2315 | 0.3611 | G | 0.03697 | 0.5329 | PTPRN2 |
| 7 | kgp1915155 | rs7804373 | 157509978 | T | 0.1019 | 0.2037 | C | 0.03749 | 0.4433 | PTPRN2 |
| 7 | kgp1010008 | rs13243294 | 157468453 | A | 0.1792 | 0.08333 | G | 0.03751 | 2.402 | PTPRN2 |
| 7 | kgp11078957 | rs60801665 | 157637684 | T | 0.1111 | 0.03704 | A | 0.03767 | 3.25 | PTPRN2 |
| 7 | kgp10093067 | rs78982627 | 157646434 | T | 0.1111 | 0.03704 | C | 0.03767 | 3.25 | PTPRN2 |
| 7 | kgp4559962 | rs28452577 | 157655716 | A | 0.03704 | 0.1111 | G | 0.03767 | 0.3077 | PTPRN2 |
| 7 | kgp5210770 | rs12698305 | 158752863 | C | 0.5094 | 0.3679 | T | 0.03789 | 1.784 | Intergenic WDR60-VIPR2 |
| 7 | rs7780644 | rs7780644 | 158910780 | A | 0.05556 | 0.1389 | G | 0.03873 | 0.3647 | VIPR2 |
| 7 | rs2037907 | rs2037907 | 158974421 | T | 0.05556 | 0.1389 | C | 0.03873 | 0.3647 | After VIPR2 |
| 7 | kgp116446 | rs10949751 | 158758910 | C | 0.3611 | 0.5 | A | 0.03928 | 0.5652 | Intergenic WDR60-VIPR2 |
| 7 | kgp9055248 | rs10279216 | 157690083 | A | 0.1852 | 0.3056 | C | 0.03982 | 0.5165 | PTPRN2 |
| 7 | kgp4897716 | rs10268910 | 157311053 | A | 0.3981 | 0.537 | G | 0.0408 | 0.5703 | intergenic DNAJB6-PTPRN2 |
| 7 | rs740994 | rs740994 | 157308348 | T | 0.5288 | 0.3889 | C | 0.04087 | 1.764 | intergenic DNAJB6-PTPRN2 |
| 7 | kgp8251436 | rs111597359 | 157572123 | A | 0.1111 | 0.213 | G | 0.04224 | 0.462 | PTPRN2 |
| 7 | kgp4077538 | rs6963718 | 157767010 | T | 0.1481 | 0.2593 | C | 0.04263 | 0.4969 | PTPRN2 |
| 7 | kgp594584 | rs73523919 | 158857197 | C | 0.1481 | 0.2593 | T | 0.04263 | 0.4969 | VIPR2 |
| 7 | kgp9052841 | rs428495 | 159007339 | A | 0.2593 | 0.1481 | G | 0.04263 | 2.012 | After VIPR2 |
| 7 | rs1188974 | rs1188974 | 158665748 | A | 0.08333 | 0.1759 | G | 0.0428 | 0.4258 | WDR60 |
| 7 | kgp8827307 | rs6459896 | 158506580 | G | 0.3868 | 0.2593 | A | 0.04597 | 1.802 | intergenic NCAPG2-ESYT2 |
| 7 | rs4716494 | rs4716494 | 157583166 | G | 0.4167 | 0.287 | A | 0.04607 | 1.774 | PTPRN2 |
| 7 | rs2952635 | rs2952635 | 157508424 | A | 0.1481 | 0.06481 | G | 0.04711 | 2.509 | PTPRN2 |
| 7 | kgp5841260 | rs6955096 | 158138905 | G | 0.06481 | 0.1481 | A | 0.04711 | 0.3985 | PTPRN2 |
| 7 | kgp13683794 | rs58134544 | 158178405 | A | 0.06481 | 0.1481 | G | 0.04711 | 0.3985 | PTPRN2 |
| 7 | kgp1428511 | rs2730229 | 158789265 | T | 0.2963 | 0.4259 | C | 0.04734 | 0.5675 | Intergenic WDR60-VIPR2 |
| 7 | kgp13740396 | rs75318171 | 158402030 | C | 0.0463 | 0.1204 | T | 0.0489 | 0.3547 | intergenic PTPRN2-NCAPG2 |
| 7 | kgp2128349 | rs10282118 | 158665987 | T | 0.1667 | 0.2778 | C | 0.04953 | 0.52 | WDR60 |

**Table L. Significant p-values (1% level) of the association test in the African enriched chromosome 22 region in FCG.** MAF>5% in all individuals in the comparison group.

| Chr | ID chip | ID ensembl | Position in GRCh37 | Allele | Frequency in fever | Frequency in asymptomatic control | Other allele | P-value | OR | Location |
| --- | --- | --- | --- | --- | --- | --- | --- | --- | --- | --- |
| 22 | kgp22834527 | rs12166565 | 33057937 | G | 0.05405 | 0.2095 | A | 7.73E-05 | 0.2157 | SYN3 |
| 22 | kgp3741267 | rs715527 | 28571028 | G | 0.4054 | 0.2095 | T | 0.0002592 | 2.573 | TTC28 |
| 22 | rs132893 |  | 25556577 | G | 0.3716 | 0.1824 | A | 0.0002763 | 2.65 | KIAA1671 |
| 22 | kgp12472779 | rs139646 | 25232862 | C | 0.5811 | 0.3716 | T | 0.0003089 | 2.345 | SGSM1 |
| 22 | rs9613570 |  | 28545264 | C | 0.3311 | 0.1528 | A | 0.0003839 | 2.745 | TTC28 |
| 22 | kgp5633784 | rs9612883 | 25569680 | T | 0.3581 | 0.1757 | C | 0.0003884 | 2.618 | KIAA1671 |
| 22 | rs1467387 |  | 25931372 | C | 0.5068 | 0.3082 | T | 0.0005341 | 2.306 |  |
| 22 | kgp7956758 | rs9613658 | 29036465 | G | 0.3243 | 0.1554 | A | 0.0006664 | 2.609 | TTC28/RN7SL162P |
| 22 | rs3788374 |  | 24954832 | C | 0.1892 | 0.3649 | T | 0.0007333 | 0.4062 | GUCD1/SNRPD3 |
| 22 | rs738786 |  | 24034288 | A | 0.3851 | 0.5811 | G | 0.0007433 | 0.4516 | RGL4/GUSBP11 |
| 22 | rs3788372 |  | 24894856 | G | 0.3851 | 0.5811 | A | 0.0007433 | 0.4516 | UPB1/ADORA2A-AS1 |
| 22 | kgp6733693 | rs111794229 | 33257378 | A | 0.1622 | 0.3311 | C | 0.0007487 | 0.391 | SYN3-TIMP3 |
| 22 | rs1065314 |  | 33258288 | C | 0.1622 | 0.3311 | T | 0.0007487 | 0.391 | SYN3-TIMP3 |
| 22 | rs5754315 |  | 33259064 | T | 0.1622 | 0.3311 | C | 0.0007487 | 0.391 | SYN3-TIMP3 |
| 22 | rs9704 |  | 24936970 | T | 0.1959 | 0.3716 | C | 0.0008021 | 0.4121 | GUCD1 |
| 22 | rs762286 |  | 24970107 | A | 0.1959 | 0.3716 | G | 0.0008021 | 0.4121 | SNRPD3 |
| 22 | kgp7965377 | rs738820 | 24947452 | C | 0.1892 | 0.363 | T | 0.0008515 | 0.4094 | GUCD1/SNRPD3 |
| 22 | rs5754425 |  | 33498970 | C | 0.2568 | 0.1081 | T | 0.0009295 | 2.85 |  |
| 22 | kgp12174355 | rs1012366 | 33048925 | A | 0.25 | 0.4324 | C | 0.0009329 | 0.4375 | SYN3 |
| 22 | kgp1859085 |  | 33103668 | G | 0.2568 | 0.4392 | T | 0.0009854 | 0.4411 | SYN3 |
| 22 | rs5754222 |  | 33103968 | C | 0.2568 | 0.4392 | T | 0.0009854 | 0.4411 | SYN3 |
| 22 | rs7288416 |  | 28799436 | A | 0.3176 | 0.1554 | G | 0.001028 | 2.529 | TTC28 |
| 22 | kgp10178980 | rs5760470 | 24935250 | T | 0.1892 | 0.3581 | C | 0.001117 | 0.4182 | GUCD1 |
| 22 | rs3176991 |  | 24964128 | T | 0.1892 | 0.3581 | C | 0.001117 | 0.4182 | SNRPD3 |
| 22 | rs9608667 |  | 28540492 | G | 0.3649 | 0.1959 | A | 0.001217 | 2.357 | TTC28 |
| 22 | rs9621414 |  | 32628436 | G | 0.1014 | 0.2432 | A | 0.001228 | 0.3509 | CPSF1P1 |
| 22 | kgp321192 | rs3788338 | 23412058 | A | 0.1419 | 0.2973 | G | 0.001241 | 0.3908 | RTDR1/GNAZ |
| 22 | rs2267184 |  | 33258050 | T | 0.1689 | 0.3311 | C | 0.001275 | 0.4107 | SYN3-TIMP3 |
| 22 | kgp5592480 | rs713672 | 24955700 | T | 0.1892 | 0.3562 | C | 0.001298 | 0.4218 | GUCD1/SNRPD3 |
| 22 | kgp7602289 | rs2330555 | 24013288 | A | 0.5775 | 0.3873 | G | 0.001345 | 2.162 | GUSBP11 |
| 22 | kgp2769414 | rs4822886 | 27869118 | A | 0.5 | 0.3151 | G | 0.001349 | 2.174 |  |
| 22 | kgp915943 | rs4822939 | 28172577 | T | 0.4257 | 0.25 | G | 0.001398 | 2.224 | MN1 |
| 22 | rs2283885 |  | 33248902 | A | 0.3041 | 0.1486 | G | 0.0014 | 2.502 | SYN3-TIMP3 |
| 22 | kgp2839573 | rs1543803 | 33265922 | T | 0.1486 | 0.3041 | G | 0.0014 | 0.3996 | SYN3 |
| 22 | kgp11323669 | rs11703116 | 24031712 | T | 0.5 | 0.3176 | G | 0.001412 | 2.149 | RGL4/GUSBP11 |
| 22 | kgp4592016 | rs10439908 | 32664241 | G | 0.1081 | 0.25 | A | 0.001454 | 0.3636 | CPSF1P1 |
| 22 | rs8140340 |  | 24029646 | G | 0.5 | 0.3176 | T | 0.001462 | 2.149 | RGL4/GUSBP11 |
| 22 | kgp9213634 | rs5754189 | 33048338 | C | 0.2568 | 0.4324 | T | 0.001473 | 0.4534 | SYN3 |
| 22 | kgp11645856 | rs5994482 | 32570873 | G | 0.3311 | 0.5135 | C | 0.001487 | 0.4689 |  |
| 22 | rs1427384 |  | 33257322 | G | 0.1824 | 0.3446 | A | 0.001543 | 0.4244 | SYN3-TIMP3 |
| 22 | rs5994479 |  | 32557939 | G | 0.1554 | 0.3108 | T | 0.001568 | 0.408 | C22orf42 |
| 22 | rs12168696 |  | 32611862 | T | 0.1554 | 0.3108 | G | 0.001568 | 0.408 | SLC5A4 |
| 22 | kgp4837399 | rs718810 | 33077216 | A | 0.1554 | 0.3108 | G | 0.001568 | 0.408 | SYN3 |
| 22 | kgp11899862 | rs1811024 | 24016474 | T | 0.1712 | 0.3311 | C | 0.001591 | 0.4174 | GUSBP11 |
| 22 | kgp15051213 |  | 24959974 | T | 0.1918 | 0.3562 | A | 0.001638 | 0.4289 | SNRPD3 |
| 22 | kgp2155857 | rs7291871 | 33107956 | G | 0.223 | 0.3919 | T | 0.001638 | 0.4453 | SYN3 |
| 22 | kgp12412442 | rs738785 | 24022631 | C | 0.4054 | 0.5878 | T | 0.001697 | 0.4781 | GUSBP11 |
| 22 | rs9621409 |  | 32616657 | T | 0.1149 | 0.2568 | C | 0.0017 | 0.3757 | SLC5A4 |
| 22 | kgp7349471 | rs10427608 | 23702130 | C | 0.08108 | 0.2095 | T | 0.001724 | 0.333 |  |
| 22 | kgp5219543 | rs17415919 | 29094828 | C | 0.3176 | 0.1622 | A | 0.001743 | 2.404 | CHEK2 |
| 22 | rs9619301 |  | 33107311 | T | 0.1959 | 0.3581 | C | 0.001827 | 0.4368 | SYN3 |
| 22 | rs713725 |  | 25563892 | A | 0.4054 | 0.2365 | G | 0.001854 | 2.201 | KIAA1671 |
| 22 | kgp9700777 | rs427736 | 28173898 | T | 0.4324 | 0.2603 | C | 0.001931 | 2.165 | MN1 |
| 22 | rs5763284 |  | 29899724 | G | 0.02027 | 0.1081 | A | 0.002049 | 0.1707 | THOC5 |
| 22 | rs5763288 |  | 29902382 | A | 0.02027 | 0.1081 | C | 0.002049 | 0.1707 | THOC5 |
| 22 | rs7290686 |  | 29380995 | A | 0.3151 | 0.162 | G | 0.00234 | 2.38 | ZNRF3 |
| 22 | kgp3094863 | rs11703629 | 25572582 | A | 0.3851 | 0.223 | G | 0.002425 | 2.183 | KIAA1671 |
| 22 | rs3747120 |  | 25591847 | T | 0.3851 | 0.223 | C | 0.002425 | 2.183 | KIAA1671/CRYBB3 |
| 22 | rs5998300 |  | 32602718 | T | 0.09459 | 0.223 | C | 0.002514 | 0.3641 | RFPL2 |
| 22 | kgp1754888 | rs7289865 | 33091812 | C | 0.2297 | 0.3919 | A | 0.002578 | 0.4628 | SYN3 |
| 22 | kgp3341898 | rs6003842 | 24030367 | A | 0.1419 | 0.2838 | G | 0.002863 | 0.4173 | RGL4/GUSBP11 |
| 22 | kgp15029637 | rs12169377 | 33292189 | T | 0.03378 | 0.1284 | C | 0.002872 | 0.2374 | SYN3 |
| 22 | kgp7063734 | rs4821129 | 33495528 | A | 0.2574 | 0.1154 | G | 0.003051 | 2.657 |  |
| 22 | kgp695626 | rs242069 | 33241769 | C | 0.4932 | 0.3243 | T | 0.003119 | 2.028 | SYN3-TIMP3 |
| 22 | kgp475255 | rs62220914 | 23480925 | A | 0.1149 | 0.02703 | G | 0.003249 | 4.672 | RTDR1 |
| 22 | rs5752249 |  | 26388706 | A | 0.1486 | 0.0473 | G | 0.003359 | 3.517 | MYO18B |
| 22 | kgp2441341 | rs4821127 | 33488404 | T | 0.2635 | 0.1284 | C | 0.003404 | 2.429 |  |
| 22 | rs137489 |  | 33262935 | C | 0.3311 | 0.1824 | T | 0.00342 | 2.218 | SYN3-TIMP3 |
| 22 | kgp3877577 | rs5752565 | 27881196 | T | 0.3904 | 0.5608 | C | 0.003445 | 0.5016 |  |
| 22 | rs7284984 |  | 26371211 | C | 0.1081 | 0.2365 | A | 0.003452 | 0.3913 | MYO18B |
| 22 | kgp11374980 | rs71321088 | 26793038 | G | 0.02027 | 0.1014 | A | 0.003517 | 0.1834 |  |
| 22 | rs1474813 |  | 27881885 | G | 0.3919 | 0.5608 | A | 0.003621 | 0.5047 |  |
| 22 | kgp9234600 | rs738503 | 27798492 | G | 0.4054 | 0.5743 | A | 0.003652 | 0.5053 |  |
| 22 | rs13054331 |  | 23407063 | A | 0.1233 | 0.2569 | G | 0.003697 | 0.4067 | RTDR1 |
| 22 | rs695267 |  | 28899748 | A | 0.4315 | 0.2703 | C | 0.003763 | 2.049 | TTC28 |
| 22 | rs6003484 |  | 23379985 | A | 0.3264 | 0.4932 | G | 0.003764 | 0.4978 |  |
| 22 | rs9613574 |  | 28604583 | T | 0.2432 | 0.1149 | C | 0.003971 | 2.477 | TTC28 |
| 22 | rs713974 |  | 27210691 | A | 0.1959 | 0.3446 | G | 0.003985 | 0.4635 |  |
| 22 | kgp10131755 | rs5760866 | 25523876 | C | 0.4595 | 0.2973 | T | 0.004023 | 2.009 | KIAA1671 |
| 22 | rs5998500 |  | 32859702 | C | 0.473 | 0.3108 | T | 0.004269 | 1.99 | BPIFC |
| 22 | kgp9692981 | rs136446 | 32582254 | C | 0.4797 | 0.3176 | T | 0.004384 | 1.981 | RFPL2 |
| 22 | kgp2299977 | rs9606957 | 32861083 | T | 0.4797 | 0.3176 | G | 0.004384 | 1.981 | BPIFC |
| 22 | kgp9696891 | rs8135522 | 25232052 | T | 0.2095 | 0.3581 | C | 0.004563 | 0.4749 | SGSM1 |
| 22 | kgp8505410 | rs5762925 | 29369398 | C | 0.4122 | 0.2568 | A | 0.004604 | 2.03 | ZNRF3 |
| 22 | rs13055470 |  | 29371761 | T | 0.3176 | 0.1757 | C | 0.00463 | 2.184 | ZNRF3 |
| 22 | rs929271 |  | 30638226 | G | 0.3176 | 0.1757 | T | 0.00463 | 2.184 | LIF |
| 22 | kgp4061366 | rs3753082 | 30638721 | A | 0.3176 | 0.1757 | G | 0.00463 | 2.184 | LIF |
| 22 | rs7291990 |  | 32569263 | C | 0.1486 | 0.2838 | T | 0.004745 | 0.4407 |  |
| 22 | kgp3582539 | rs1475978 | 33092669 | G | 0.1486 | 0.2838 | A | 0.004745 | 0.4407 | SYN3 |
| 22 | kgp4573649 | rs5761252 | 26213574 | T | 0.03378 | 0.1216 | C | 0.004764 | 0.2525 | MYO18B |
| 22 | rs1894536 |  | 33434836 | A | 0.5068 | 0.3446 | G | 0.004783 | 1.954 | SYN3 |
| 22 | rs4822568 |  | 25585047 | A | 0.4189 | 0.2635 | G | 0.004808 | 2.015 | KIAA1671 |
| 22 | rs4822549 |  | 25231422 | A | 0.2162 | 0.3649 | G | 0.004855 | 0.4802 | SGSM1 |
| 22 | rs5998267 |  | 32554985 | A | 0.5135 | 0.3514 | G | 0.004866 | 1.949 | C22orf42 |
| 22 | kgp194736 | rs67261683 | 27159903 | C | 0.08784 | 0.2027 | T | 0.005045 | 0.3788 |  |
| 22 | rs5762758 |  | 29109036 | T | 0.3082 | 0.1689 | C | 0.005049 | 2.192 | CHEK2 |
| 22 | kgp8454216 | rs6005437 | 27837974 | T | 0.1284 | 0.2568 | C | 0.0051 | 0.4264 |  |
| 22 | kgp7450654 | rs5762197 | 27887471 | A | 0.3784 | 0.5405 | C | 0.005124 | 0.5174 |  |
| 22 | rs5998278 |  | 32565036 | T | 0.5405 | 0.3784 | G | 0.005124 | 1.933 |  |
| 22 | rs2056962 |  | 28672406 | A | 0.2297 | 0.1081 | G | 0.005233 | 2.461 | TTC28 |
| 22 | kgp7884201 | rs73416473 | 27459563 | T | 0.1419 | 0.0473 | C | 0.005427 | 3.331 |  |
| 22 | rs5761092 |  | 25904257 | G | 0.1824 | 0.07432 | A | 0.005434 | 2.779 | CRYBB2P1 |
| 22 | rs5763241 |  | 29839168 | C | 0.1824 | 0.07432 | T | 0.005434 | 2.779 | RFPL1-RFPL1S |
| 22 | kgp1614972 | rs5760872 | 25532237 | A | 0.3784 | 0.2297 | C | 0.005439 | 2.041 | KIAA1671 |
| 22 | kgp5514293 | rs60054645 | 23533072 | T | 0.1081 | 0.02703 | G | 0.005456 | 4.364 | BCR |
| 22 | kgp1158398 | rs5759577 | 23415146 | T | 0.1622 | 0.06081 | C | 0.005603 | 2.989 | RTDR1/GNAZ |
| 22 | rs3761426 |  | 29688632 | G | 0.1622 | 0.06081 | T | 0.005603 | 2.989 | EWSR1 |
| 22 | kgp7381141 | rs138559,rs199566619 | 25664438 | A | 0.2973 | 0.4527 | G | 0.005756 | 0.5115 |  |
| 22 | rs4822888 |  | 27892976 | T | 0.2973 | 0.4527 | G | 0.005756 | 0.5115 |  |
| 22 | kgp6290805 | rs4822890 | 27894302 | A | 0.2973 | 0.4527 | G | 0.005756 | 0.5115 |  |
| 22 | kgp1430016 | rs59671056 | 31556988 | G | 0.1301 | 0.04054 | A | 0.005892 | 3.541 | RNF185 |
| 22 | rs9625239 |  | 27867567 | A | 0.05405 | 0.1507 | G | 0.006205 | 0.3221 |  |
| 22 | rs2040435 |  | 33263431 | T | 0.25 | 0.3986 | C | 0.006303 | 0.5028 | SYN3-TIMP3 |
| 22 | rs738865 |  | 22400423 | T | 0.1892 | 0.08108 | C | 0.006522 | 2.644 |  |
| 22 | rs5754429 |  | 33501556 | A | 0.1892 | 0.08108 | G | 0.006522 | 2.644 |  |
| 22 | kgp10910039 | rs5996455 | 23379255 | A | 0.1757 | 0.3108 | G | 0.006739 | 0.4726 |  |
| 22 | kgp5114380 | rs114462986 | 27864094 | A | 0.5634 | 0.4041 | T | 0.006845 | 1.903 |  |
| 22 | rs926837 |  | 27879170 | G | 0.3767 | 0.5338 | A | 0.006856 | 0.5279 |  |
| 22 | rs2298372 |  | 23950646 | G | 0.2635 | 0.4122 | A | 0.00686 | 0.5103 | C22orf43 |
| 22 | rs12484788 |  | 27878619 | A | 0.2973 | 0.1644 | G | 0.006885 | 2.151 |  |
| 22 | kgp10350038 | rs12483800 | 27158663 | G | 0.06757 | 0.1689 | T | 0.006931 | 0.3565 |  |
| 22 | rs2157462 |  | 27340510 | A | 0.06757 | 0.1689 | C | 0.006931 | 0.3565 |  |
| 22 | rs5752710 |  | 28620907 | G | 0.277 | 0.1486 | A | 0.006975 | 2.195 | TTC28 |
| 22 | rs8142488 |  | 32615004 | G | 0.2162 | 0.3581 | T | 0.006979 | 0.4945 | SLC5A4 |
| 22 | rs9621508 |  | 33013062 | C | 0.3649 | 0.5203 | A | 0.007113 | 0.5297 | SYN3 |
| 22 | rs139647 |  | 25232918 | A | 0.3851 | 0.2397 | G | 0.007178 | 1.986 | SGSM1 |
| 22 | rs4822887 |  | 27869192 | T | 0.5338 | 0.3784 | C | 0.007273 | 1.881 |  |
| 22 | kgp3947477 | rs136438 | 32580444 | T | 0.5338 | 0.3784 | C | 0.007273 | 1.881 |  |
| 22 | rs5752014 |  | 25287889 | A | 0.5616 | 0.4054 | C | 0.007353 | 1.879 | SGSM1 |
| 22 | kgp3698852 | rs13055979 | 23474085 | A | 0.1284 | 0.25 | G | 0.007556 | 0.4419 | RTDR1 |
| 22 | kgp10057870 | rs966964 | 32997766 | C | 0.1575 | 0.06081 | T | 0.007757 | 2.888 | SYN3 |
| 22 | rs5762764 |  | 29132990 | G | 0.3243 | 0.1892 | A | 0.007789 | 2.057 | CHEK2 |
| 22 | rs5751583 |  | 23434327 | A | 0.1149 | 0.03378 | G | 0.007834 | 3.711 | RTDR1/GNAZ |
| 22 | rs713876 |  | 25509747 | A | 0.4392 | 0.2905 | G | 0.0079 | 1.912 | KIAA1671 |
| 22 | kgp7207095 | rs5752064 | 25520095 | T | 0.4392 | 0.2905 | C | 0.0079 | 1.912 | KIAA1671 |
| 22 | kgp12195444 | rs738199 | 28756581 | G | 0.3784 | 0.2365 | A | 0.008163 | 1.965 | TTC28 |
| 22 | rs6004179 |  | 24933069 | T | 0.1622 | 0.2905 | C | 0.008314 | 0.4726 | GUCD1 |
| 22 | kgp5522491 | rs139736 | 25297404 | A | 0.4527 | 0.3041 | G | 0.008373 | 1.893 | SGSM1 |
| 22 | kgp1007211 |  | 25664780 | T | 0.3041 | 0.4527 | C | 0.008373 | 0.5282 |  |
| 22 | rs2331111 |  | 25942595 | T | 0.4527 | 0.3041 | C | 0.008373 | 1.893 |  |
| 22 | rs12170895 |  | 24943294 | T | 0.07432 | 0.1757 | C | 0.008383 | 0.3768 | GUCD1 |
| 22 | rs12160660 |  | 24949306 | C | 0.07432 | 0.1757 | T | 0.008383 | 0.3768 | GUCD1/SNRPD3 |
| 22 | kgp1558224 |  | 28868540 | G | 0.3851 | 0.2432 | A | 0.008551 | 1.949 | TTC28 |
| 22 | kgp12553111 | rs6005306 | 27535572 | A | 0.4595 | 0.3108 | G | 0.008596 | 1.885 |  |
| 22 | kgp4825716 | rs56400867 | 33010072 | C | 0.1849 | 0.08108 | T | 0.008671 | 2.571 | SYN3 |
| 22 | kgp3982551 | rs9620522 | 25778419 | T | 0.1554 | 0.06081 | A | 0.008778 | 2.842 | LRP5L |
| 22 | rs5762110 |  | 27788451 | T | 0.1554 | 0.06081 | C | 0.008778 | 2.842 |  |
| 22 | rs740229 |  | 31553510 | T | 0.06081 | 0.1554 | C | 0.008778 | 0.3519 | RNF185 |
| 22 | rs11703695 |  | 27983422 | A | 0.2297 | 0.1149 | G | 0.008883 | 2.298 |  |
| 22 | kgp12478332 | rs5997656 | 30863514 | G | 0.3919 | 0.25 | A | 0.008933 | 1.933 | SEC14L3 |
| 22 | rs5996883 |  | 25669569 | T | 0.1554 | 0.2808 | C | 0.009176 | 0.4712 |  |
| 22 | kgp4288637 | rs8139822 | 24938908 | A | 0.2014 | 0.338 | G | 0.009194 | 0.4938 | GUCD1 |
| 22 | rs2301415 |  | 32341669 | G | 0.2635 | 0.1419 | A | 0.009255 | 2.164 | YWHAH-C22orf24 |
| 22 | kgp14991028 | rs6006167 | 29894362 | T | 0.02027 | 0.08904 | C | 0.00935 | 0.2117 |  |
| 22 | kgp10884348 | rs136924 | 27785823 | A | 0.4932 | 0.3446 | G | 0.009549 | 1.851 |  |
| 22 | rs2016820 |  | 27989166 | T | 0.3446 | 0.4932 | C | 0.009549 | 0.5402 |  |
| 22 | rs2078729 |  | 23443909 | A | 0.1233 | 0.04054 | G | 0.009573 | 3.328 | RTDR1/GNAZ |
| 22 | rs2298383 |  | 24825511 | C | 0.4155 | 0.5676 | T | 0.009614 | 0.5416 | ADORA2A/ADORA2A-AS1 |
| 22 | kgp12154122 | rs8142788 | 29400515 | A | 0.1824 | 0.08108 | G | 0.009945 | 2.529 | ZNRF3 |
| 22 | rs8137688 |  | 23285694 | A | 0.1216 | 0.2365 | G | 0.009959 | 0.447 |  |

*1.7- XP-EHH selection test*

Fig M and Tables M-N report the results of the XP-EHH positive selection test performed in HCG.


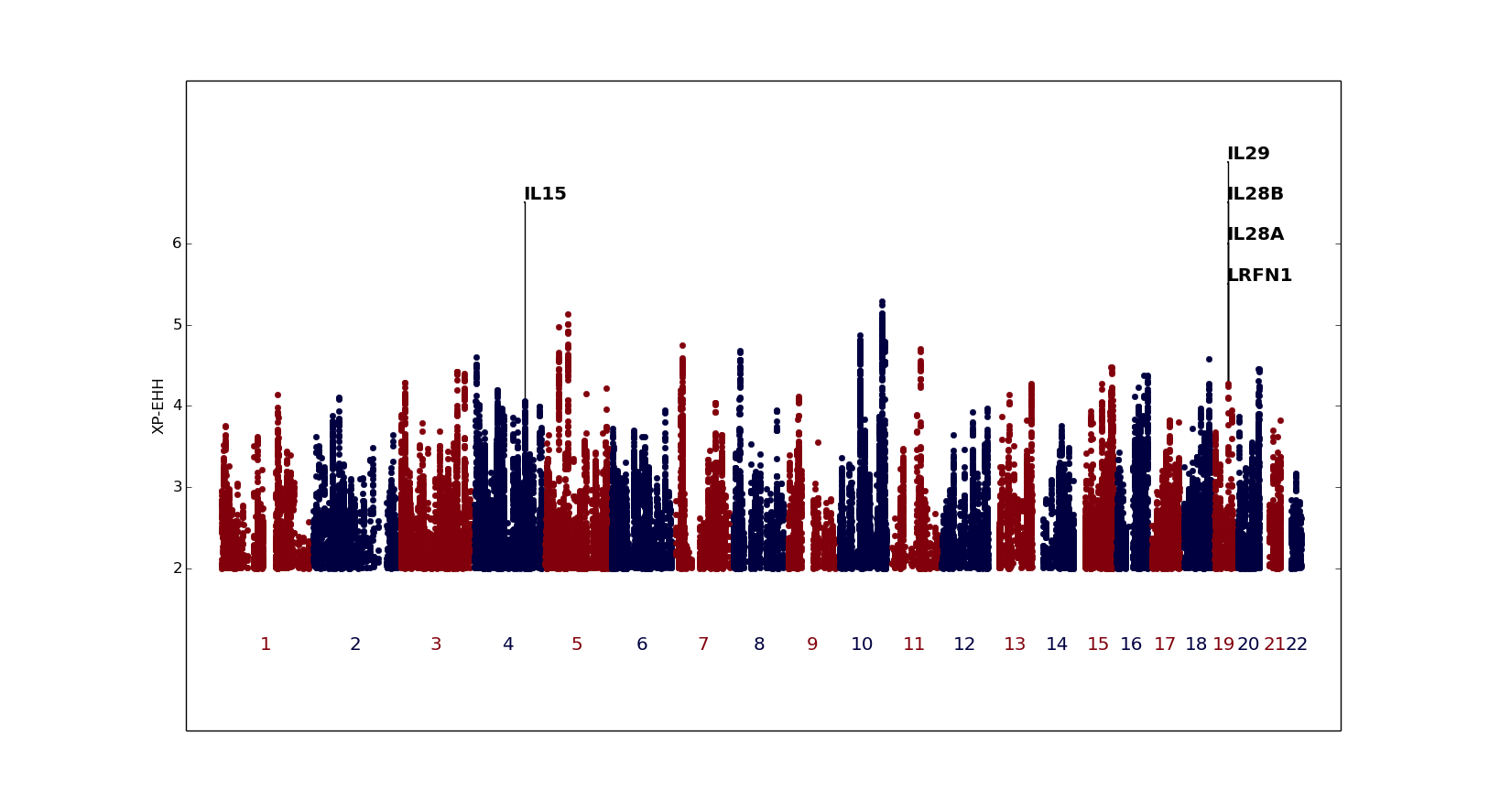


A


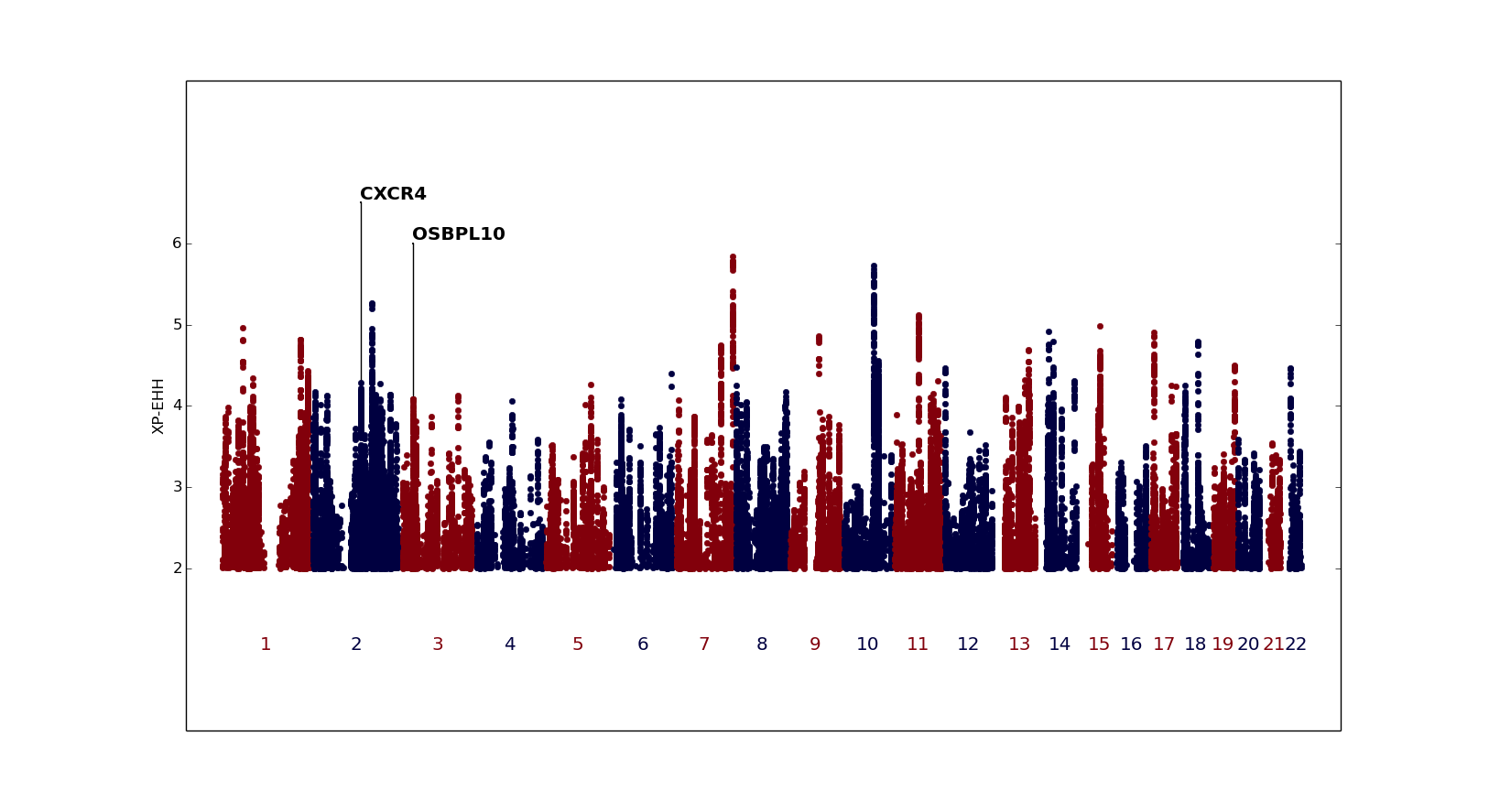


B

**Fig M. Manhattan plots for the XP-EHH selection measure.** **(a)** In asymptomatic and **(b)** haemorrhagic individuals in the HCG.

**Table M. XP-EHH values for the HCG, selected in the asymptomatic/control.**

| Chrom | Beginning of window | End of window | Max XP-EHH | Position max XP-EHH | Snps total | Genes at 5kb around maximum value | Genes in window |
| --- | --- | --- | --- | --- | --- | --- | --- |
| 10 | 121700001 | 121800000 | 5,286435225 | 121781774 | 78 |  | SEC23IP |
| 5 | 64900001 | 65000000 | 5,129792719 | 64951033 | 43 | C5orf44 | TRIM23 C5orf44 SGTB |
| 5 | 39300001 | 39400000 | 4,968499774 | 39376988 | 74 | DAB2 | DAB2 C9 |
| 10 | 59600001 | 59700000 | 4,872812663 | 59607602 | 52 |  |  |
| 10 | 121800001 | 121900000 | 4,808072957 | 121838117 | 73 |  |  |
| 10 | 59500001 | 59600000 | 4,80387557 | 59590682 | 37 |  |  |
| 10 | 128200001 | 128300000 | 4,787339195 | 128211885 | 115 | C10orf90 | C10orf90 |
| 7 | 19400001 | 19500000 | 4,741894147 | 19418564 | 82 |  |  |
| 11 | 84200001 | 84300000 | 4,703624633 | 84248067 | 53 | DLG2 | DLG2 |
| 10 | 59400001 | 59500000 | 4,700919649 | 59491198 | 64 |  |  |
| 8 | 17400001 | 17500000 | 4,682344544 | 17487373 | 196 | PDGFRL | SLC7A2 PDGFRL |
| 4 | 6300001 | 6400000 | 4,597803829 | 6303354 | 105 | WFS1 | PPP2R2C WFS1 |
| 18 | 70700001 | 70800000 | 4,576477103 | 70781718 | 80 |  |  |
| 5 | 64800001 | 64900000 | 4,55539689 | 64871133 | 36 | PPWD1 | TRIM23 CENPK PPWD1 |
| 10 | 59900001 | 60000000 | 4,547048752 | 59909204 | 33 |  | IPMK |
| 15 | 97500001 | 97600000 | 4,472701692 | 97561675 | 102 |  |  |
| 15 | 99800001 | 99900000 | 4,471449139 | 99865609 | 42 | LRRC28 | LRRC28 |
| 20 | 59000001 | 59100000 | 4,457324596 | 59074914 | 79 |  |  |
| 3 | 159900001 | 160000000 | 4,421413614 | 159993490 | 51 | IFT80 | IFT80 |
| 3 | 180000001 | 180100000 | 4,396022751 | 180067423 | 48 |  |  |
| 16 | 78700001 | 78800000 | 4,376741418 | 78714567 | 72 | WWOX | WWOX |
| 16 | 87400001 | 87500000 | 4,370985001 | 87469883 | 59 | ZCCHC14 | FBXO31 ZCCHC14 MAP1LC3B |
| 5 | 39400001 | 39500000 | 4,342802542 | 39408937 | 59 | DAB2 | DAB2 |
| 3 | 10800001 | 10900000 | 4,282746587 | 10840276 | 94 |  | SLC6A11 |
| 19 | 39700001 | 39800000 | 4,27709677 | 39743165 | 52 |  | IL29 IL28B IL28A LRFN1 |
| 13 | 110000001 | 110100000 | 4,274758226 | 110053658 | 91 |  |  |
| 15 | 70500001 | 70600000 | 4,274065324 | 70517708 | 93 |  |  |
| 16 | 63100001 | 63200000 | 4,225722079 | 63194487 | 54 |  |  |
| 15 | 99600001 | 99700000 | 4,223230297 | 99641985 | 146 | SYNM | SYNM TTC23 |
| 5 | 173700001 | 173800000 | 4,213896106 | 173767646 | 109 |  |  |
| 4 | 65200001 | 65300000 | 4,19857231 | 65264183 | 42 | TECRL | TECRL |
| 7 | 15800001 | 15900000 | 4,195714089 | 15868471 | 72 |  |  |
| 10 | 60000001 | 60100000 | 4,174593901 | 60014352 | 36 | IPMK | IPMK CISD1 UBE2D1 |
| 5 | 115800001 | 115900000 | 4,145751853 | 115861632 | 103 | SEMA6A | SEMA6A |
| 1 | 157300001 | 157400000 | 4,134192381 | 157370618 | 98 |  |  |
| 13 | 49300001 | 49400000 | 4,133386216 | 49365482 | 65 |  |  |
| 16 | 51600001 | 51700000 | 4,120827366 | 51623435 | 66 |  |  |
| 9 | 28600001 | 28700000 | 4,112226052 | 28680131 | 45 | LINGO2 | LINGO2 |
| 8 | 16100001 | 16200000 | 4,086075662 | 16175982 | 57 |  |  |
| 9 | 28700001 | 28800000 | 4,075262392 | 28701333 | 91 | LINGO2 | LINGO2 |
| 15 | 100700001 | 100800000 | 4,067647132 | 100732119 | 148 | ADAMTS17 | ADAMTS17 |
| 4 | 3200001 | 3300000 | 4,060338347 | 3236135 | 61 | HTT | HTT C4orf44 |
| 4 | 142600001 | 142700000 | 4,055108269 | 142636313 | 40 | IL15 | IL15 |
| 4 | 6200001 | 6300000 | 4,053029563 | 6299914 | 43 | WFS1 | WFS1 JAKMIP1 |
| 15 | 97900001 | 98000000 | 4,052769725 | 97946731 | 88 |  |  |
| 4 | 65700001 | 65800000 | 4,046626881 | 65769447 | 59 |  |  |
| 7 | 113700001 | 113800000 | 4,03530726 | 113754232 | 40 | FOXP2 | FOXP2 |
| 10 | 60100001 | 60200000 | 4,023807751 | 60110335 | 36 | UBE2D1 | TFAM UBE2D1 |
| 4 | 14100001 | 14200000 | 4,016245791 | 14128732 | 112 |  |  |
| 4 | 142500001 | 142600000 | 4,009756496 | 142538358 | 33 |  | IL15 |
| 15 | 101700001 | 101800000 | 4,002694225 | 101798865 | 91 |  | CHSY1 |
| 4 | 184200001 | 184300000 | 3,993206797 | 184294510 | 84 |  | WWC2 CLDN22 CLDN24 |
| 4 | 77200001 | 77300000 | 3,97248636 | 77288833 | 43 | CCDC158 | CCDC158 STBD1 FAM47E |
| 18 | 48700001 | 48800000 | 3,971393706 | 48726876 | 51 | MEX3C | MEX3C |
| 12 | 130300001 | 130400000 | 3,963505283 | 130348817 | 79 | TMEM132D | TMEM132D |
| 19 | 48500001 | 48600000 | 3,949360753 | 48517676 | 108 | ELSPBP1 | CABP5 PLA2G4C ELSPBP1 |
| 8 | 121800001 | 121900000 | 3,949207515 | 121847643 | 83 |  | SNTB1 |
| 6 | 151000001 | 151100000 | 3,948408013 | 151004289 | 81 | PLEKHG1 | PLEKHG1 |
| 15 | 39300001 | 39400000 | 3,937128367 | 39369688 | 74 |  |  |
| 12 | 88600001 | 88700000 | 3,924043177 | 88697433 | 34 |  |  |
| 15 | 93900001 | 94000000 | 3,92243751 | 93993996 | 120 |  |  |
| 20 | 58800001 | 58900000 | 3,906767263 | 58854231 | 95 |  |  |
| 3 | 300001 | 400000 | 3,891210279 | 324149 | 101 | CHL1 | CHL1 |
| 11 | 73200001 | 73300000 | 3,889491349 | 73265980 | 25 | FAM168A | FAM168A |
| 7 | 19300001 | 19400000 | 3,885800313 | 19322503 | 80 |  |  |
| 2 | 56000001 | 56100000 | 3,881876089 | 56035925 | 169 |  | EFEMP1 |
| 16 | 75200001 | 75300000 | 3,880123846 | 75204443 | 71 | ZFP1 | BCAR1 ZFP1 CTRB2 CTRB1 |
| 4 | 83600001 | 83700000 | 3,878291653 | 83652046 | 92 | SCD5 | SCD5 |
| 13 | 28400001 | 28500000 | 3,870123404 | 28437272 | 72 |  | PDX1 |
| 10 | 113600001 | 113700000 | 3,869990153 | 113649319 | 55 |  |  |
| 20 | 4400001 | 4500000 | 3,863860635 | 4417880 | 73 |  |  |
| 4 | 110200001 | 110300000 | 3,857577879 | 110278135 | 43 |  | COL25A1 |
| 1 | 159600001 | 159700000 | 3,852247863 | 159680817 | 64 | CRP | CRP |
| 16 | 67700001 | 67800000 | 3,837790195 | 67796315 | 22 | RANBP10 | GFOD2 RANBP10 C16orf48 C16orf86 |
| 10 | 72700001 | 72800000 | 3,836724191 | 72748629 | 155 |  |  |
| 9 | 26200001 | 26300000 | 3,831967152 | 26290353 | 63 |  |  |
| 17 | 51200001 | 51300000 | 3,827869702 | 51208664 | 50 |  |  |

**Table N. XP-EHH values for the HCG, selected in the haemorrhagic.**

| Chrom | Beginning of window | End of window | Max XP-EHH | Position max XP-EHH | Snps total | Genes at 5kb around maximum value | Genes in window |
| --- | --- | --- | --- | --- | --- | --- | --- |
| 7 | 158800001 | 158900000 | 5,836721722 | 158896436 | 76 | VIPR2 | VIPR2 |
| 8 | 158900001 | 159000000 | 5,786592922 | 158902741 | 44 |  |  |
| 10 | 85300001 | 85400000 | 5,726043941 | 85308054 | 40 |  |  |
| 10 | 85200001 | 85300000 | 5,611102148 | 85296654 | 66 |  |  |
| 2 | 167500001 | 167600000 | 5,261959454 | 167587749 | 38 |  |  |
| 11 | 67000001 | 67100000 | 5,123332402 | 67024534 | 31 | KDM2A | ANKRD13D KDM2A SSH3 ADRBK1 |
| 15 | 58700001 | 58800000 | 4,982226893 | 58703895 | 151 |  | LIPC |
| 1 | 58800001 | 58900000 | 4,955770026 | 58833911 | 111 |  |  |
| 14 | 27600001 | 27700000 | 4,910618129 | 27626887 | 60 |  |  |
| 17 | 10300001 | 10400000 | 4,898952057 | 10330168 | 25 | MYH8 | MYH1 MYH4 MYH8 |
| 9 | 80200001 | 80300000 | 4,855898853 | 80209604 | 76 | GNA14 | GNA14 |
| 1 | 223000001 | 223100000 | 4,818695342 | 223057033 | 46 | DISP1 | DISP1 |
| 11 | 67100001 | 67200000 | 4,798088168 | 67124992 | 37 | POLD4 | CLCF1 RPS6KB2 TBC1D10C POLD4 PPP1CA RAD9A CARNS1 |
| 14 | 40200001 | 40300000 | 4,796389225 | 40217339 | 56 |  |  |
| 18 | 43400001 | 43500000 | 4,793277828 | 43429966 | 53 | EPG5 | SIGLEC15 EPG5 |
| 1 | 223100001 | 223200000 | 4,772330866 | 223124916 | 75 | DISP1 | DISP1 |
| 7 | 125500001 | 125600000 | 4,742376176 | 125511106 | 53 |  |  |
| 7 | 125400001 | 125500000 | 4,733368449 | 125481946 | 59 |  |  |
| 13 | 93900001 | 94000000 | 4,686517609 | 93990654 | 67 | GPC6 | GPC6 |
| 15 | 58600001 | 58700000 | 4,616068123 | 58680904 | 164 |  |  |
| 10 | 85400001 | 85500000 | 4,594321658 | 85419023 | 97 |  |  |
| 10 | 97300001 | 97400000 | 4,55633197 | 97387248 | 80 | ALDH18A1 | SORBS1 ALDH18A1 |
| 13 | 94000001 | 94100000 | 4,547823932 | 94005805 | 95 | GPC6 | GPC6 |
| 10 | 97000001 | 97100000 | 4,524858226 | 97074129 | 112 | SORBS1 | PDLIM1 SORBS1 |
| 17 | 10400001 | 10500000 | 4,514444707 | 10404046 | 35 | MYH1 | MYH1 MYH2 |
| 19 | 58400001 | 58500000 | 4,499447375 | 58419180 | 66 | ZNF417 | ZNF814 ZNF418 ZNF417 C19orf18 ZNF256 ZNF606 |
| 8 | 2300001 | 2400000 | 4,470578676 | 2377503 | 128 |  |  |
| 22 | 19100001 | 19200000 | 4,460018582 | 19158116 | 70 | SLC25A1 | DGCR2 SLC25A1 CLTCL1 TSSK2 GSC2 DGCR14 |
| 12 | 300001 | 400000 | 4,45935233 | 349706 | 94 | SLC6A13 | SLC6A12 SLC6A13 KDM5A |
| 10 | 97100001 | 97200000 | 4,443209044 | 97118644 | 80 | SORBS1 | SORBS1 |
| 1 | 244100001 | 244200000 | 4,433448453 | 244167170 | 99 |  |  |
| 6 | 168100001 | 168200000 | 4,398456898 | 168102894 | 42 |  | C6orf123 |
| 1 | 243800001 | 243900000 | 4,368935272 | 243804158 | 30 | AKT3 | AKT3 |
| 1 | 243700001 | 243800000 | 4,358994793 | 243766921 | 24 | AKT3 | AKT3 |
| 1 | 87700001 | 87800000 | 4,342551693 | 87757861 | 82 |  | LMO4 |
| 13 | 81700001 | 81800000 | 4,322524159 | 81779413 | 47 |  |  |
| 11 | 120600001 | 120700000 | 4,307839965 | 120697099 | 37 | GRIK4 | GRIK4 |
| 14 | 101600001 | 101700000 | 4,305088344 | 101663335 | 73 |  |  |
| 2 | 135900001 | 136000000 | 4,288352094 | 135959272 | 36 | ZRANB3 | RAB3GAP1 ZRANB3 |
| 2 | 190400001 | 190500000 | 4,269483838 | 190442152 | 82 | SLC40A1 | SLC40A1 |
| 5 | 126400001 | 126500000 | 4,258743855 | 126451362 | 39 |  | C5orf63 |
| 18 | 5000001 | 5100000 | 4,251121933 | 5027542 | 85 |  |  |
| 17 | 59400001 | 59500000 | 4,247990548 | 59472403 | 87 | BCAS3 TBX2 | BCAS3 TBX2 C17orf82 |
| 17 | 70800001 | 70900000 | 4,233999257 | 70858149 | 84 | SLC39A11 | SLC39A11 |
| 2 | 167600001 | 167700000 | 4,213711883 | 167633880 | 52 |  |  |
| 2 | 6100001 | 6200000 | 4,176861486 | 6157537 | 89 |  |  |
| 8 | 142200001 | 142300000 | 4,174236453 | 142237733 | 98 | SLC45A4 | DENND3 SLC45A4 |
| 2 | 136800001 | 136900000 | 4,171351582 | 136802456 | 49 |  | CXCR4 |
| 2 | 136700001 | 136800000 | 4,151983636 | 136793114 | 39 |  | DARS |
| 11 | 108500001 | 108600000 | 4,149172053 | 108571682 | 29 | DDX10 | DDX10 |
| 13 | 95700001 | 95800000 | 4,142069807 | 95727349 | 139 | ABCC4 | ABCC4 |
| 2 | 178500001 | 178600000 | 4,135773725 | 178596672 | 88 | PDE11A | PDE11A |
| 2 | 219300001 | 219400000 | 4,135227399 | 219345931 | 28 | USP37 | USP37 VIL1 |
| 2 | 40400001 | 40500000 | 4,131842839 | 40424444 | 86 | SLC8A1 | SLC8A1 |
| 2 | 136000001 | 136100000 | 4,128758092 | 136002500 | 44 | ZRANB3 | ZRANB3 |
| 3 | 157300001 | 157400000 | 4,125047068 | 157318743 | 69 | C3orf55 | C3orf55 |
| 2 | 178600001 | 178700000 | 4,110522775 | 178607998 | 61 | PDE11A | PDE11A |
| 1 | 243900001 | 244000000 | 4,109150296 | 243919773 | 30 | AKT3 | AKT3 |
| 13 | 28800001 | 28900000 | 4,105938961 | 28837296 | 58 | PAN3 | FLT1 PAN3 |
| 11 | 99900001 | 100000000 | 4,10387358 | 99955299 | 93 | CNTN5 | CNTN5 |
| 10 | 85100001 | 85200000 | 4,090828366 | 85195727 | 56 |  |  |
| 3 | 31900001 | 32000000 | 4,087124005 | 31979027 | 110 | OSBPL10 | OSBPL10 |
| 6 | 23500001 | 23600000 | 4,081274313 | 23586112 | 92 |  |  |
| 4 | 104400001 | 104500000 | 4,062326106 | 104468034 | 64 |  |  |
| 8 | 29700001 | 29800000 | 4,042598384 | 29798823 | 55 |  |  |
| 8 | 29800001 | 29900000 | 4,035895889 | 29807552 | 79 |  |  |

*1.8- In depth investigation of OSBPL10 and RXRA genes*

The six SNPs of the detected OSBPL10 haplotype are highly linked in the 1000 Genomes dataset populations representative of European (Great-Britain; Fig N), Asian (Chinese; Fig O) and African (Yoruba; Fig P) ancestries.


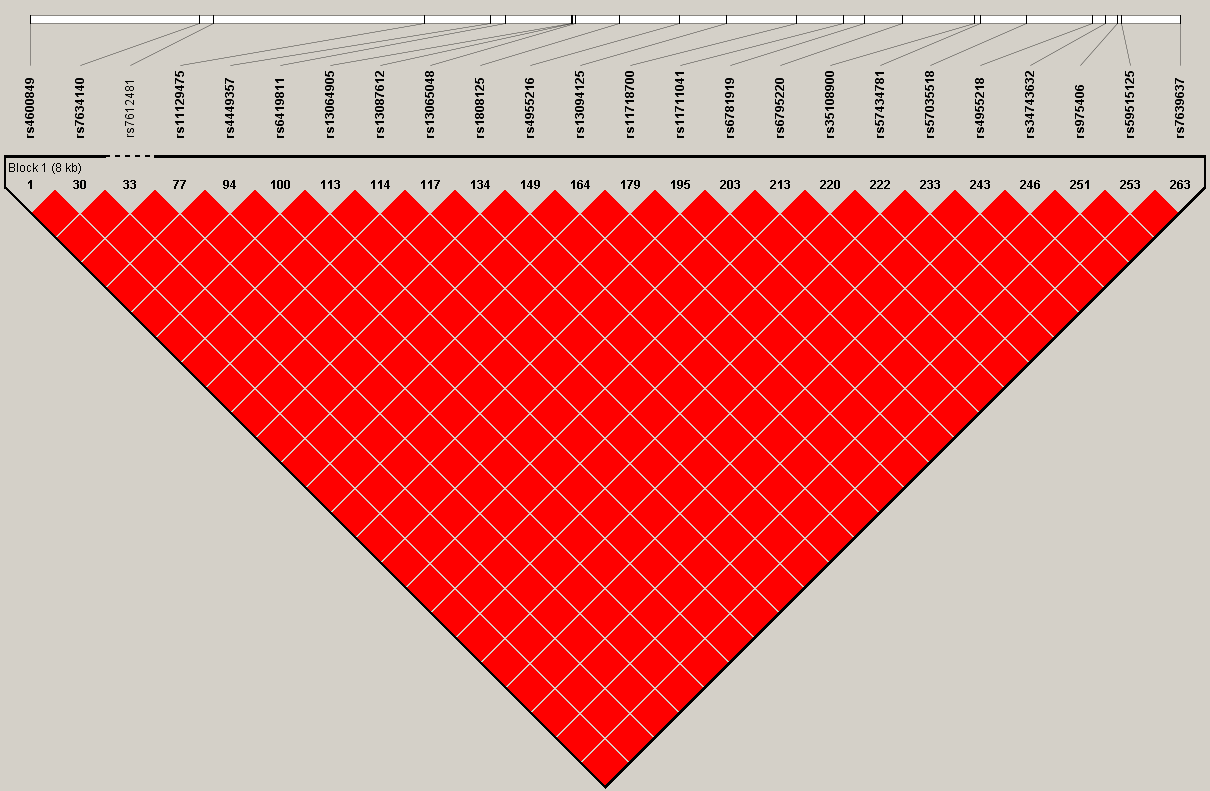


**Fig N. LD (r^2^) values for the *OSBPL10* region in the Great-Britain population (GBR) from 1000 Genomes database.**


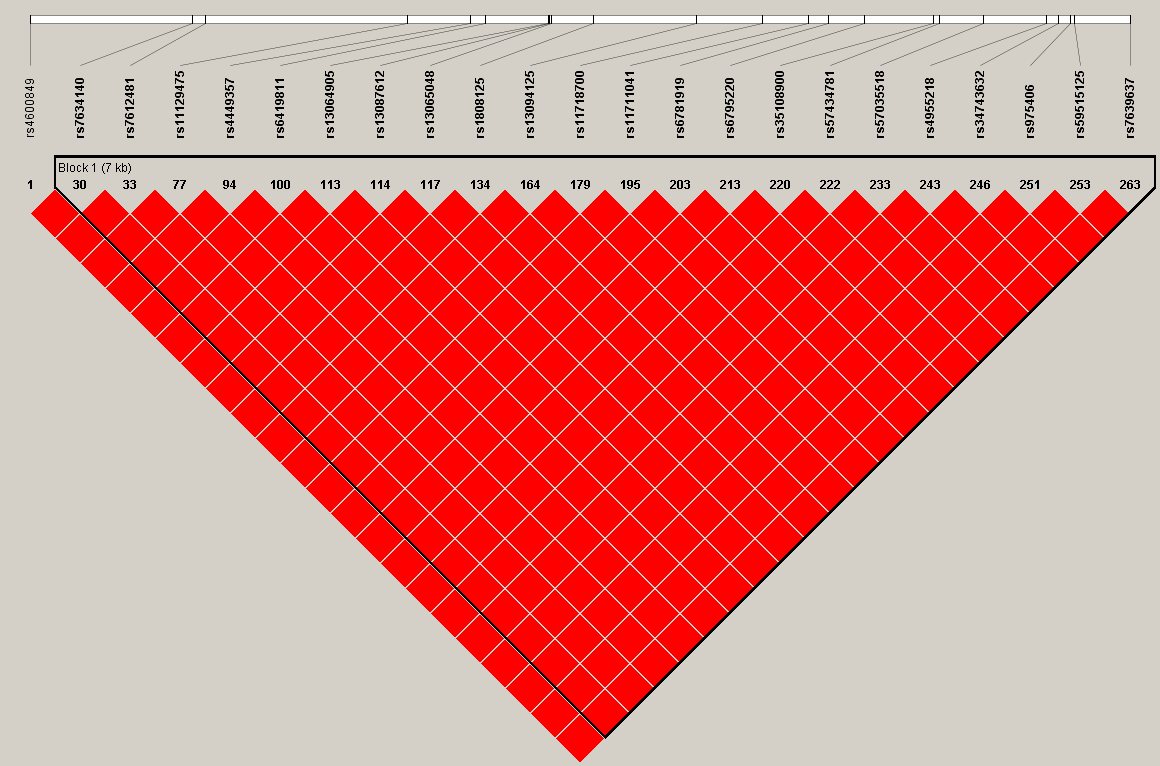


**Fig O. LD (r^2^) values for the *OSBPL10* region in the Chinese population (CHS) from 1000 Genomes database.**


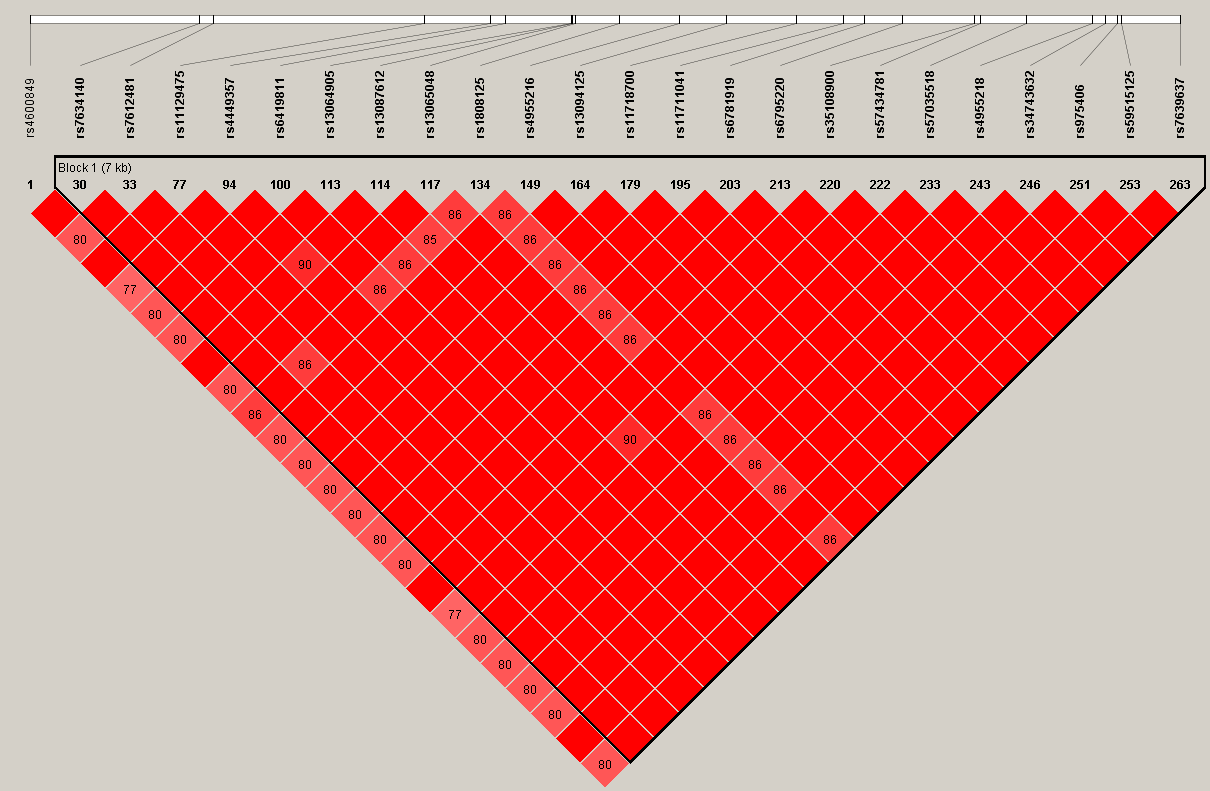


**Fig P. LD (r^2^) values for the *OSBPL10* region in the Yoruba population (YRI) from 1000 Genomes database.**

As can be seen in Fig Q, there are two promoters for *OSBPL10* and one for the overlapping *ZNF860* gene, but the haplotype region is 2.5 kb from the OSBPL10_1 and ZNF860 promoters (OSBPL10_2 is even farther). EPDNew tool also infers three HMR conserved transcription factor binding sites within the beginning of the haplotype region, one being recognised by *STAT5A* (signal transducer and activator of transcription 5A), which is activated by a number of cytokine and growth hormone receptors, playing a key role in the transformation of B and T lymphocytes [[9](#_ENREF_9)]. The haplotype region can also be related with a few weaker enhancer regions inferred by HaploReg (Table O). Two haplotype SNPs (rs4600849 and rs11129475) are located in weak enhancers detected in different cell types, and two contiguous SNPs not in the chip (rs35108900 and rs57434781) are located in a weak enhancer detected in the hepatic HepG2 cell line. Several SNPs located in the *OSBPL10* haplotype are recognised by many transcription factors, including *STAT* and *RXRA*.

**Fig Q. The location of the promoters for OSBPL10 and ZNF860 genes (inside the red square) and the haplotype associated with dengue in Cuba (in the blue square).** K562 is a cell line derived from blood from human (chronic myelogenous leukemia).

**Table O. Results from the HaploReg version 2 tool for SNPs located on the *OSBPL10* haplotype.** SNPs detected in our chip are highlighted on light brown; other SNPs/indels identified in the region on the 1000 Genomes project as having high differences in allele frequencies between African and European populations are highlighted in blue.

| chr | pos | rsID | ref | alt | AFR | AMR | ASN | EUR | Promoter_ENCODE | Enhancer_ENCODE | Promoter_Roadmap | Enhancer_Roadmap | DNAse | Proteins | eQTL | Motifs |
| --- | --- | --- | --- | --- | --- | --- | --- | --- | --- | --- | --- | --- | --- | --- | --- | --- |
| 3 | 32027672 | rs4600849 | T | C | 0.78 | 0.46 | 0.76 | 0.28 | . | . | . | ESO,14_Enh;CD19.P,9_TxEnhG1;ADI.NUC,11_EnhWk1 | Duke,GM19240,None | . | . | . |
| 3 | 32028907 | rs7634140 | A | T | 0.65 | 0.33 | 0.31 | 0.19 | . | . | . | . | . | . | . | Hltf;Lhx3_2;Mef2_disc1;Mef2_known3;Mef2_known5;Mef2_known6 |
| 3 | 32029010 | rs7612481 | G | A | 0.78 | 0.35 | 0.43 | 0.2 | . | . | . | . | . | . | . | INSM1 |
| 3 | 32030544 | rs11129475 | C | T | 0.65 | 0.33 | 0.31 | 0.19 | . | K562,7_Weak_Enhancer | . | PANC,12_EnhWk2 | . | . | . | CEBPD;ERalpha-a_known2;RXRA_known5;Zbtb3 |
| 3 | 32031024 | rs4449357 | C | T | 0.78 | 0.35 | 0.31 | 0.2 | . | . | . | . | . | . | . | Nkx6-1_1;Sox_1 |
| 3 | 32031135 | rs6419811 | A | G | 0.78 | 0.35 | 0.43 | 0.2 | . | . | . | . | . | . | . | PU.1_known2 |
| 3 | 32031615 | rs13064905 | A | C | 0.78 | 0.35 | 0.43 | 0.2 | . | . | . | . | . | . | . | . |
| 3 | 32031622 | rs13087612 | T | C | 0.76 | 0.35 | 0.43 | 0.2 | . | . | . | . | . | . | . | . |
| 3 | 32031643 | rs13065048 | A | G | 0.78 | 0.35 | 0.44 | 0.2 | . | . | . | . | . | . | . | Brachyury_2;Eomes;FAC1;RREB-1_2;TBX5_1;TBX5_2 |
| 3 | 32031962 | rs1808125 | A | G | 0.66 | 0.4 | 0.64 | 0.2 | . | . | . | . | . | . | . | Pbx-1_4;Pou5f1_known2 |
| 3 | 32032397 | rs4955216 | G | T | 0.78 | 0.35 | 0.31 | 0.2 | . | . | . | . | . | NT2-D1,ZNF274,UCD,None | . | . |
| 3 | 32032743 | rs13094125 | T | G | 0.78 | 0.35 | 0.31 | 0.2 | . | . | . | . | . | . | . | . |
| 3 | 32033248 | rs11718700 | A | C | 0.78 | 0.35 | 0.31 | 0.2 | . | . | . | . | . | . | . | CTCF_disc4;Mrg1::Hoxa9_1;Pou2f2_known11;YY1_disc2;YY1_known2;YY1_known6 |
| 3 | 32033595 | rs11711041 | C | G | 0.78 | 0.35 | 0.43 | 0.2 | . | . | . | . | . | . | . | AP-2rep;SREBP_known3;Zbtb3 |
| 3 | 32033748 | rs6781919 | C | G | 0.78 | 0.35 | 0.31 | 0.2 | . | . | . | . | . | . | . | Nkx2_7;Pou3f2_2 |
| 3 | 32034023 | rs6795220 | T | C | 0.78 | 0.35 | 0.31 | 0.2 | . | . | . | . | . | . | . | AP-1_disc6 |
| 3 | 32034547 | rs35108900 | G | A | 0.75 | 0.34 | 0.31 | 0.2 | . | HepG2,7_Weak_Enhancer | . | . | . | . | . | AP-1_disc8;BCL_disc4;Elf5;GATA_disc3;Irf_disc3;Irf_known1;Irf_known3;Irf_known7;Irf_known9;Mef2_disc2;PU.1_disc2;Pax-5_disc3;RXRA_disc4;STAT_disc3;p300_disc5 |
| 3 | 32034591 | rs57434781 | G | A | 0.77 | 0.35 | 0.31 | 0.2 | . | HepG2,7_Weak_Enhancer | . | . | . | . | . | Pou2f2_known2;Pou2f2_known8 |
| 3 | 32034927 | rs57035518 | T | C | 0.77 | 0.34 | 0.31 | 0.2 | . | . | . | . | . | . | . | LXR_2;Maf_disc2;T3R |
| 3 | 32035406 | rs4955218 | T | A | 0.78 | 0.35 | 0.31 | 0.2 | . | . | . | . | . | HepG2,MAFF,Stanford,None;HepG2,MAFK,Stanford,None | . | . |
| 3 | 32035499 | rs34743632 | C | A | 0.78 | 0.35 | 0.31 | 0.2 | . | . | . | . | . | HepG2,MAFF,Stanford,None;HepG2,MAFK,Stanford,None;HepG2,MAFK,Stanford,None | . | Foxa_disc1;Foxa_known1;Foxa_known2;Foxa_known3;Foxc1_1;Foxd1_1;Foxd1_2;Foxf1;Foxi1;Foxj1_1;Foxj1_2;Foxj2_1;Foxk1;Foxl1_1;Foxo_1;Foxo_2;Foxo_3;Foxo_4;Foxp1;Foxq1;HDAC2_disc2;Sox_6;TCF12_disc2;p300_disc3 |
| 3 | 32035587 | rs975406 | T | C | 0.78 | 0.35 | 0.31 | 0.2 | . | . | . | . | . | HepG2,MAFF,Stanford,None;HepG2,MAFK,Stanford,None;HepG2,MAFK,Stanford,None | . | Nanog_disc1;Pou2f2_disc1;Pou2f2_known7;Pou3f3;Pou5f1_disc2;TATA_disc9 |
| 3 | 32035619 | rs59515125 | C | T | 0.65 | 0.33 | 0.31 | 0.19 | . | . | . | . | . | HepG2,MAFF,Stanford,None;HepG2,MAFK,Stanford,None;HepG2,MAFK,Stanford,None | . | CEBPB_known4;Evi-1_5;Hand1_1;Osf2_2;Smad_2 |
| 3 | 32036042 | rs7639637 | A | C | 0.78 | 0.35 | 0.31 | 0.2 | . | . | . | . | . | . | . | . |

## **Cell lines:** *ADI.NUC*-Adipose Nuclei; *CD19.P*-CD19 Primary Cells; *ESO*-esophagus; *GM19240*-Yoruba immortalised cell line; *HepG2*-hepatocellular carcinoma; *K562*-leukemia; *NT2-D1*-malignant pluripotent embryonal carcinoma.

Besides the *OSBPL10* haplotype, the segment immediately 5’ to it (within the gene) has some SNPs with significant p-values in the association test (Table I), that are regulatory regions in several cell types (Fig R).


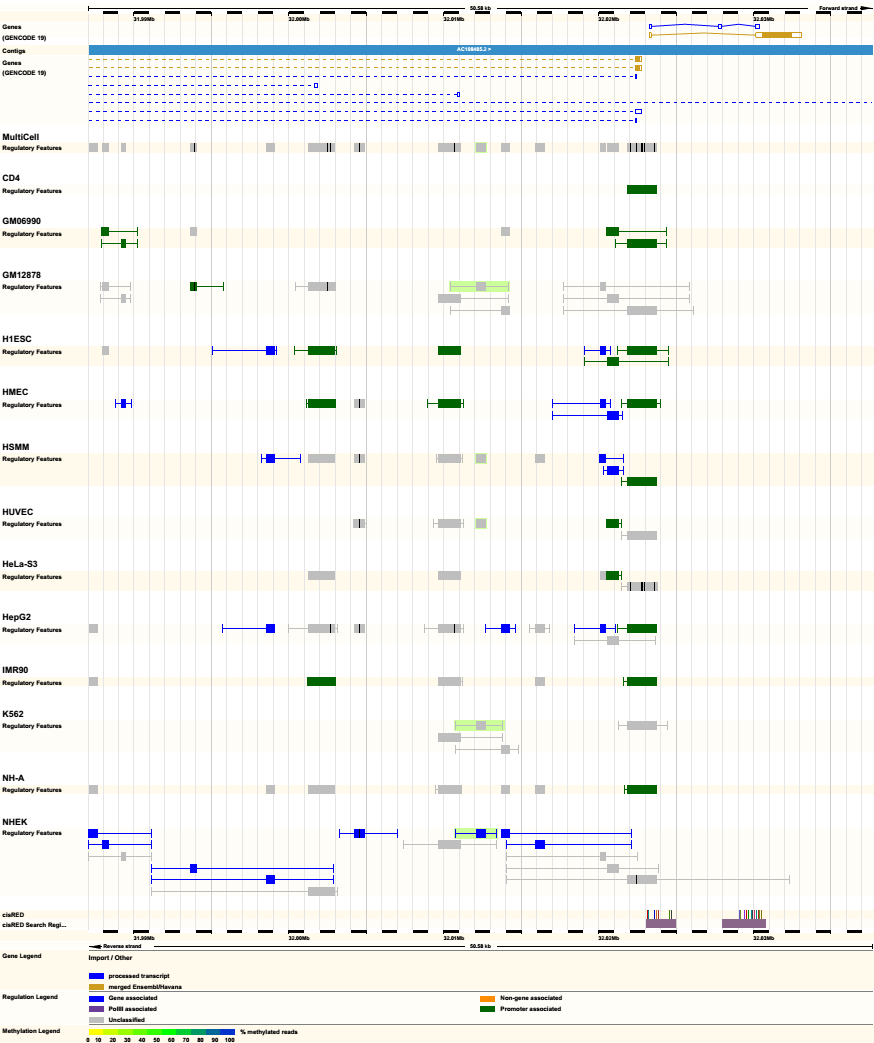


**Fig R. Ensembl regulatory features in several cell lines, for the region 5’ before the haplotype in *OSBPL10* gene.** Cell lines: CD4-human CD4 T cell; GM06990-B-lymphocyte, lymphoblastoid; GM12878-B-lymphocyte, lymphoblastoid; H1ESC-embryonic stem cells; HMEC-mammary epithelial cells; HSMM-skeletal muscle myoblasts; Huvec- umbilical vein endothelial cells; HeLa-S3-epithelial carcinoma cells; HepG2-hepatocellular carcinoma; IMR90-fetal lung fibroblast; K562-leukemia; NH-A-normal human astrocytes; NHEK-myelogenous leukaemia.


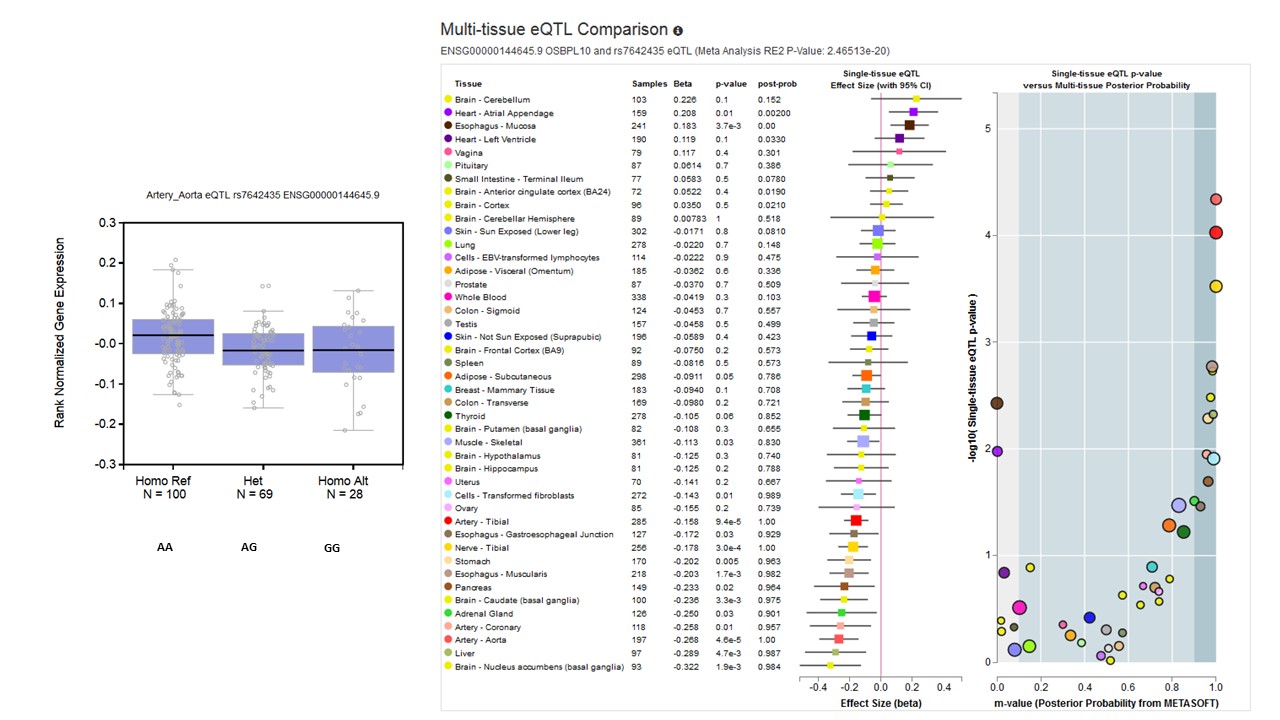


**Fig S. GTEx inferred eQTL rs7642435.** Expression of *OSBPL10* gene by genotype and multi-tissue comparison.

According to the HGDP selection browser [[10](#_ENREF_10)], this extended *OSBPL10* segment (haplotype and immediate 5’ region) has been under positive selection in the African population of Yoruba (iHS measure; Fig T). It is also detected as positively selected through XP-EHH (Fig M and Table N) in Cuban HCG.


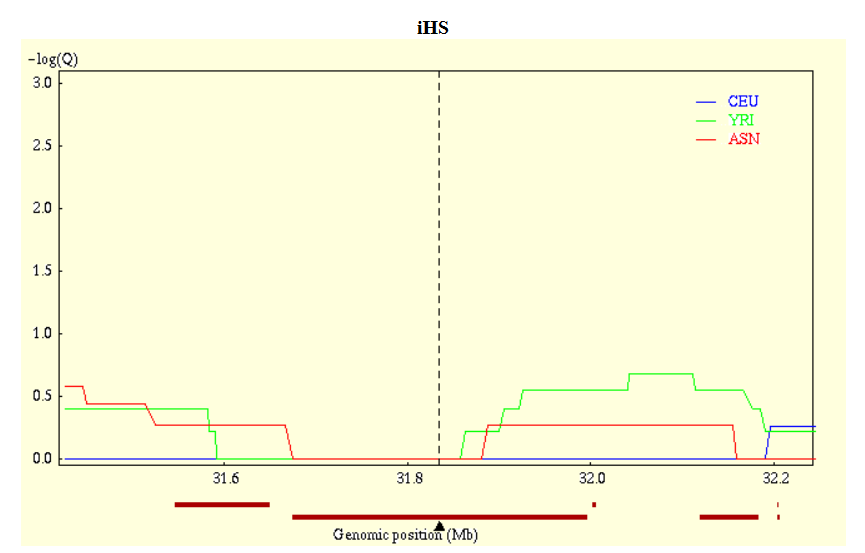


A


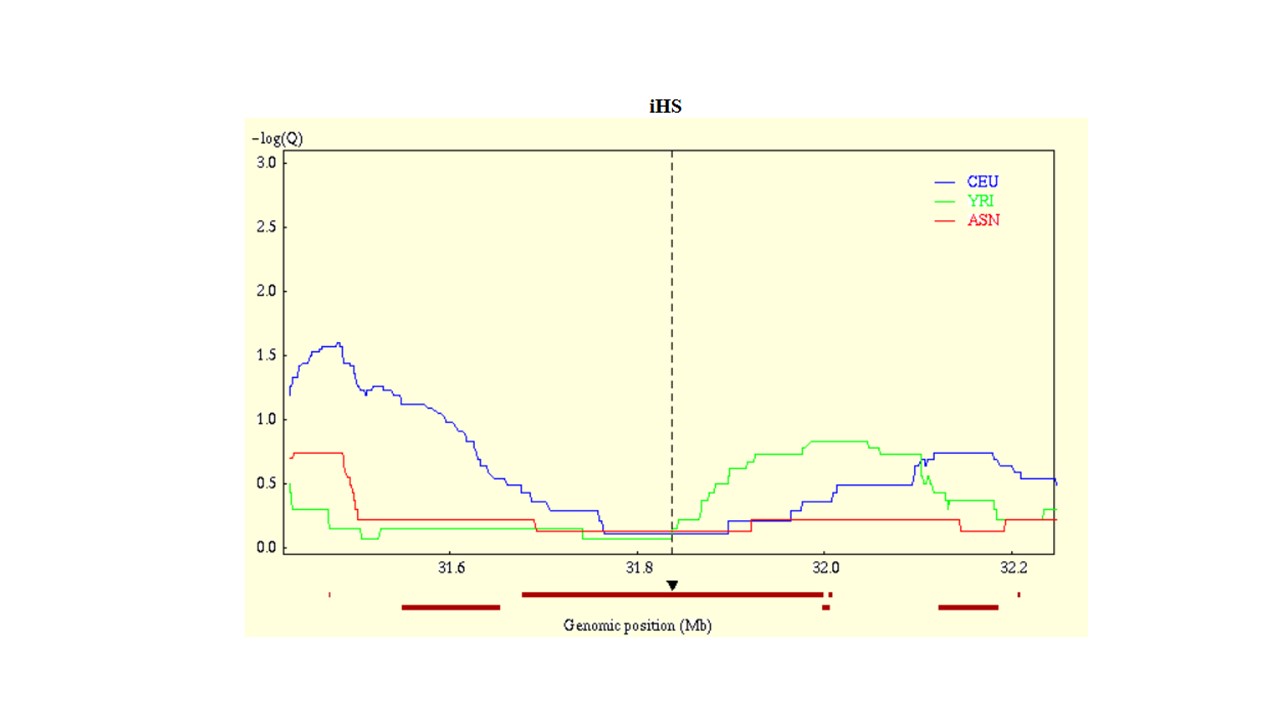


B

**Fig T. iHS values centred in *OSBPL10* gene (indicated by the arrow tip) provided in Haplotter tool in HGDP selection browser [**[**10**](#_ENREF_10)**], based on HapMap data phase I (A) and phase II (B).**

**Table P. Results from the HaploReg version 2 tool for SNPs located on the *RXRA*-*COL5A1* region.** SNPs detected in our chip are highlighted on light brown; other SNPs/indels identified in the region on the 1000 Genomes project as having high differences in allele frequencies between African and European populations are highlighted in blue.

| chr | pos | rsID | ref | alt | AFR | AMR | ASN | EUR | Promoter_ENCODE | Enhancer_ENCODE | Promoter_Roadmap | Enhancer_Roadmap | DNAse | Proteins | eQTL | Motifs |
| --- | --- | --- | --- | --- | --- | --- | --- | --- | --- | --- | --- | --- | --- | --- | --- | --- |
| Region chr9_137205000-137215000 | | | |  |  |  |  |  |  |  |  |  |  |  |  |  |
| 9 | 137205188 | rs12339163 | A | G | 0.36 | 0.1 | 0.13 | 0.12 | HepG2,3_Poised_Promoter | . | . | . | . | . | . | DMRT5;SIX5_known1;SIX5_known2;STAT_known7 |
| 9 | 137207142 | rs12339944 | C | T | 0.22 | 0.03 | 0.01 | 0 | HepG2,3_Poised_Promoter | . | . | PFF.2,15_EnhP;ST.SMUS28,14_Enh;SK.MUS63,12_EnhWk2;BN.GM2,11_EnhWk1;HD.CD184EC,11_EnhWk1;IPS.DF19,11_EnhWk1;BN.FE2,12_EnhWk2;IPS.20,12_EnhWk2;H1.BMP4DM,12_EnhWk2;HUES6,12_EnhWk2;BN.CC,12_EnhWk2;H1.DNP,11_EnhWk1;SPL,14_Enh | Duke,Fibrobl,None | . | . | AP-4_2;LBP-1_2;Nanog_disc3 |
| 9 | 137208916 | rs11185643 | G | A | 0.19 | 0.03 | 0 | 0 | . | K562,6_Weak_Enhancer;NHLF,6_Weak_Enhancer | COL.SMUS,6_TssD2 | PFF.2,15_EnhP;SK.MUS63,12_EnhWk2;IPS.DF19,11_EnhWk1;SPL,14_Enh;BN.SN,11_EnhWk1;PFF.1,15_EnhP;NCC.COR2,11_EnhWk1;CD34.MBP1536,11_EnhWk1;HRT.FE,13_EnhA;LIV.A,11_EnhWk1;CD34.MBP1508,11_EnhWk1;SK.MUS,12_EnhWk2;CD34.MBP1562,11_EnhWk1;COL.MUC32,12_EnhWk2;NCC.GED2,12_EnhWk2;CD34.MBP1549,11_EnhWk1;CD34.MBP1480,11_EnhWk1;CD34.P,12_EnhWk2;BN.MFL,12_EnhWk2;R.SMUS,11_EnhWk1;BN.FE0,13_EnhA;SK.MUS62,12_EnhWk2;LV,11_EnhWk1;ADI.MSC,12_EnhWk2;BR.MYO,11_EnhWk1;MUS.SC,11_EnhWk1;BM.MSC,11_EnhWk1;PANC,11_EnhWk1;BN.FE2,11_EnhWk1;DUO.SMUS,12_EnhWk2;CHON.BMMSC,11_EnhWk1;DUO.MUC61,11_EnhWk1;PFK.3,15_EnhP;BN.GM2,13_EnhA;ESO,12_EnhWk2;MSC.ADIPC,12_EnhWk2;IMR90,13_EnhA;LNG.FE,13_EnhA;PFK.2,15_EnhP;R.MUC31,12_EnhWk2;ST.SMUS28,11_EnhWk1 | AWG,GM12878,None;AWG,K562,None;Duke,FibroP,None;Duke,HSMM_emb,None;Duke,ProgFib,None;Duke,Stellate,None;UW,Monocytes-CD14+_RO01746,None | GM12878,EBF1,HudsonAlpha,None;GM12878,EBF1,Stanford,None;GM12878,PAX5C20,HudsonAlpha,None;GM12891,NFKB,Stanford,None;GM18951,NFKB,Stanford,None;SK-N-SH_RA,USF1,HudsonAlpha,None | . | STAT_known11 |
| 9 | 137210046 | rs188708573 | C | A | 0.2 | 0.04 | 0.01 | 0.01 | . | HSMM,7_Weak_Enhancer;NHLF,7_Weak_Enhancer | PFF.1,1_TssP | IPS.DF19,11_EnhWk1;SPL,14_Enh;CD34.MBP1536,11_EnhWk1;HRT.FE,13_EnhA;CD34.MBP1562,11_EnhWk1;BR.MYO,11_EnhWk1;MUS.SC,11_EnhWk1;BM.MSC,11_EnhWk1;DUO.SMUS,12_EnhWk2;CHON.BMMSC,11_EnhWk1;MSC.ADIPC,12_EnhWk2;IMR90,13_EnhA;HD.CD56MESC,11_EnhWk1;H1.BMP4DT,11_EnhWk1;ST.SMUS28,11_EnhWk1;BN.GM2,14_Enh;BN.FE0,14_Enh;ADI.MSC,11_EnhWk1;H1.DNP,14_Enh;PFF.2,13_EnhA | AWG,HSMM,None;Duke,AoSMC,None;Duke,Fibrobl,None;Duke,Osteobl,None;Duke,ProgFib,None;UW,CD34+_Mobilized,None;UW,NB4,None | . | . | Egr-1_known5;MOVO-B;NRSF_disc5;RREB-1_1;SREBP_known2;Sin3Ak-20_disc6;Spz1_1;WT1;YY1_disc3;ZNF219;Zfp281;Zfp740;Znf143_disc3 |
| 9 | 137210863 | rs11185644 | T | C | 0.52 | 0.28 | 0.09 | 0.18 | . | HepG2,7_Weak_Enhancer;HSMM,7_Weak_Enhancer | SPL,4_TssWk | HRT.FE,13_EnhA;CD34.MBP1562,11_EnhWk1;MUS.SC,11_EnhWk1;BM.MSC,11_EnhWk1;CHON.BMMSC,11_EnhWk1;IMR90,13_EnhA;HD.CD56MESC,11_EnhWk1;H1.BMP4DT,11_EnhWk1;ST.SMUS28,11_EnhWk1;BN.GM2,14_Enh;BN.FE0,14_Enh;H1.DNP,14_Enh;PFF.2,13_EnhA;SK.MUS63,15_EnhP;PFF.1,15_EnhP;LIV.A,13_EnhA;PFM.2,12_EnhWk2 | Duke,FibroP,None;Duke,Hepatocytes,None | HepG2,USF1,HudsonAlpha,None | . | CACD_2;EBF_disc2;Roaz_1;Roaz_2;STAT_known2;STAT_known8;Zic_4;p300_disc2 |
| 9 | 137211146 | rs944860 | C | G | 0.52 | 0.28 | 0.09 | 0.18 | . | HSMM,7_Weak_Enhancer | SPL,4_TssWk | HRT.FE,13_EnhA;MUS.SC,11_EnhWk1;BM.MSC,11_EnhWk1;CHON.BMMSC,11_EnhWk1;IMR90,13_EnhA;HD.CD56MESC,11_EnhWk1;H1.BMP4DT,11_EnhWk1;BN.GM2,14_Enh;BN.FE0,14_Enh;H1.DNP,14_Enh;PFF.2,13_EnhA;PFF.1,15_EnhP;LIV.A,13_EnhA;NCC.GED2,11_EnhWk1;IPS.DF19,11_EnhWk1 | . | . | . | CCNT2_disc1;GATA_known10;GATA_known3;Lmo2-complex_2;PU.1_disc3;SIX5_known1;SIX5_known2;Sp4;TAL1_disc1 |
| 9 | 137211298 | rs914847 | C | T | 0.29 | 0.05 | 0 | 0 | . | HepG2,7_Weak_Enhancer | SPL,4_TssWk | HRT.FE,13_EnhA;MUS.SC,11_EnhWk1;BM.MSC,11_EnhWk1;IMR90,13_EnhA;HD.CD56MESC,11_EnhWk1;H1.BMP4DT,11_EnhWk1;BN.GM2,14_Enh;BN.FE0,14_Enh;PFF.2,13_EnhA;LIV.A,13_EnhA;IPS.DF19,11_EnhWk1;H1.DMSC,11_EnhWk1;H1.DNP,13_EnhA;BN.FE2,11_EnhWk1;PFF.1,12_EnhWk2;PFM.3,14_Enh;NCC.GED2,13_EnhA | . | . | . | LXR_1;RXRA_known5;TCF12_disc5;p300_known1 |
| 9 | 137214145 | rs4917348 | A | G | 0.59 | 0.29 | 0.09 | 0.17 | HepG2,2_Weak_Promoter | . | . | LIV.A,13_EnhA;BN.GM2,13_EnhA;SPL,14_Enh;NCC.COR2,13_EnhA;CHON.BMMSC,11_EnhWk1;R.MUC31,11_EnhWk1;GAS,11_EnhWk1;DUO.MUC61,13_EnhA;BN.AC,14_Enh;PANC,11_EnhWk1;H1.BMP4DT,15_EnhP;NCC.GED2,14_Enh;HRT.FE,14_Enh;ST.MUC,11_EnhWk1;BN.FE0,14_Enh;BN.MFL,14_Enh;IPS.DF19,11_EnhWk1 | . | . | . | . |
| 9 | 137214501 | rs7871873 | C | G | 0.29 | 0.05 | 0 | 0 | . | HepG2,6_Weak_Enhancer | H1.BMP4DT,6_TssD2 | LIV.A,13_EnhA;CHON.BMMSC,11_EnhWk1;R.MUC31,11_EnhWk1;GAS,11_EnhWk1;DUO.MUC61,13_EnhA;BN.AC,14_Enh;NCC.GED2,14_Enh;HRT.FE,14_Enh;ST.MUC,11_EnhWk1;BN.MFL,14_Enh;IPS.DF19,11_EnhWk1;NCC.COR2,14_Enh;BN.GM2,11_EnhWk1;BR.MYO,11_EnhWk1;BN.ITL,14_Enh | . | T-REx-HEK293,ZNF263,UCD,None | . | Egr-1_disc2;Irf_disc3;Irf_disc5;Nrf-2_1;PU.1_known2;Pax-5_disc4;TATA_disc7;Zbtb3;p300_disc5 |
| 9 | 137214888 | rs62576287 | A | C | 0.09 | 0.01 | 0 | 0.0013 | HepG2,2_Weak_Promoter | K562,7_Weak_Enhancer | H1.DMSC,4_TssWk | LIV.A,13_EnhA;R.MUC31,11_EnhWk1;GAS,11_EnhWk1;DUO.MUC61,13_EnhA;BN.AC,14_Enh;NCC.GED2,14_Enh;HRT.FE,14_Enh;ST.MUC,11_EnhWk1;BN.MFL,14_Enh;IPS.DF19,11_EnhWk1;NCC.COR2,14_Enh;BN.GM2,11_EnhWk1;BR.MYO,11_EnhWk1;BN.ITL,14_Enh;ST.SMUS28,14_Enh;ES.I3,12_EnhWk2;BN.SN,13_EnhA;BN.AG,14_Enh;ADI.NUC,13_EnhA;CHON.BMMSC,13_EnhA | . | . | . | . |
| Region chr9_137354500-137363500 | | | |  |  |  |  |  |  |  |  |  |  |  |  |  |
| 9 | 137354553 | rs76360775 | A | G | 0.13 | 0.01 | 0 | 0 | . | . | . | CD34.MBP1480,11_EnhWk1;CD34.MBP1562,11_EnhWk1;CD34.MBP1549,11_EnhWk1;PFF.2,15_EnhP;CD34.P,11_EnhWk1;CD34.MBP1536,11_EnhWk1;CD34.MBP1508,13_EnhA;CD34.C,11_EnhWk1;H1.DMSC,11_EnhWk1 | UW,CD34+_Mobilized,None;UW,CMK,None | . | . | AP-2_disc2;AP-2_known1;AP-2_known5;AP-2_known7;EBF_disc2;HES1;Hic1_1;RREB-1_2 |
| 9 | 137354604 | rs3118551 | T | C | 0.79 | 0.87 | 0.91 | 0.96 | . | . | CD34.MBP1549,6_TssD2 | CD34.MBP1480,11_EnhWk1;PFF.2,15_EnhP;CD34.P,11_EnhWk1;CD34.MBP1536,11_EnhWk1;CD34.MBP1508,13_EnhA;CD34.C,11_EnhWk1;CD34.MBP1562,13_EnhA | UW,CMK,None;UW,Jurkat,None | . | . | . |
| 9 | 137355021 | rs3132284 | A | G | 0.79 | 0.87 | 0.91 | 0.96 | . | . | . | CD34.MBP1480,11_EnhWk1;PFF.2,15_EnhP;CD34.P,11_EnhWk1;CD34.MBP1536,11_EnhWk1;CD34.C,11_EnhWk1;CD34.MBP1549,11_EnhWk1;CD34.MBP1508,11_EnhWk1;CD34.MBP1562,11_EnhWk1 | . | . | . | Evi-1_4;HDAC2_disc6;Nkx3_3;p300_disc5 |
| 9 | 137355164 | rs3118552 | A | G | 0.79 | 0.87 | 0.91 | 0.96 | . | . | . | CD34.MBP1480,11_EnhWk1;PFF.2,15_EnhP;CD34.P,11_EnhWk1;CD34.MBP1536,11_EnhWk1;CD34.C,11_EnhWk1;CD34.MBP1549,11_EnhWk1;CD34.MBP1508,11_EnhWk1;CD34.MBP1562,11_EnhWk1 | . | . | . | BDP1_disc1;BDP1_disc3;E2F_known3;EWSR1-FLI1;GR_disc6;Myc_disc10;NRSF_disc4;PU.1_disc3;Rad21_disc10;SMC3_disc3;SP1_disc3;STAT_disc7;ZNF263_disc1 |
| 9 | 137355643 | rs943428 | G | C | 0.33 | 0.15 | 0.2 | 0.18 | . | . | . | CD34.MBP1480,11_EnhWk1;PFF.2,15_EnhP;CD34.MBP1536,11_EnhWk1;CD34.MBP1562,11_EnhWk1;IPS.DF19,11_EnhWk1 | . | . | . | AP-2_disc2;AP-2_known1;ATF3_disc2;BCL_disc9;EBF_known1;ELF1_disc3;Myc_disc10;NRSF_disc9;PLAG1;YY1_disc4;ZBTB7A_known1;Zfx |
| 9 | 137355912 | rs75679673 | G | A | 0.13 | 0.01 | 0 | 0 | . | . | . | CD34.MBP1480,11_EnhWk1;PFF.2,15_EnhP;CD34.MBP1536,11_EnhWk1;CD34.MBP1562,11_EnhWk1 | . | . | . | Brachyury_1;PPAR_1 |
| 9 | 137356228 | rs80077128 | C | T | 0.02 | 0.13 | 0.02 | 0.19 | . | . | . | PFF.2,15_EnhP | Duke,8988T,None | . | . | Ets_known9;STAT_known4 |
| 9 | 137358930 | rs114989133 | T | G | 0.11 | 0.01 | 0 | 0.02 | . | . | . | PFF.2,11_EnhWk1 | . | . | . | E2A_3;ERalpha-a_disc3;HNF4_known5;Myc_known5;RXRA_known3;SMC3_disc1;Sin3Ak-20_disc2;Sin3Ak-20_disc5;VDR_3 |
| 9 | 137359044 | rs3118555 | A | G | 0.16 | 0.67 | 0.62 | 0.72 | . | . | . | PFF.1,15_EnhP;PFF.2,9_TxEnhG1 | . | . | . | AP-2_known3;BCL_disc10;CAC-binding-protein;CCNT2_disc2;Egr-1_disc6;INSM1;Myc_disc10;SP1_known1;ZNF219 |
| 9 | 137359201 | rs3132277 | G | A | 0.01 | 0.07 | 0 | 0.13 | . | . | IPS.DF19,6_TssD2 | PFF.2,9_TxEnhG1 | AWG,HSMM,None;AWG,HSMMtube,None;AWG,MCF-7,None;AWG,NHEK,None;Duke,Fibrobl,None;Duke,Gliobla,None;Duke,H9ES,None;Duke,Ishikawa,4OHTAM_20nM_72hr;Duke,LNCaP,androgen;Duke,Osteobl,None;Duke,iPS,None;UW,H7-hESC,None;UW,NB4,None;UW,WI-38,4OHTAM_20nM_72hr | GM12878,NRSF,HudsonAlpha,None;H1-hESC,NRSF,HudsonAlpha,None;H1-hESC,NRSF,HudsonAlpha,None;HeLa-S3,NRSF,HudsonAlpha,None;K562,NRSF,HudsonAlpha,None;PFSK-1,NRSF,HudsonAlpha,None;U87,NRSF,HudsonAlpha,None | . | Egr-1_disc4;Znf143_known1 |
| 9 | 137359219 | rs4842227 | G | A | 0.05 | 0.37 | 0.6 | 0.31 | . | . | IPS.DF19,6_TssD2 | PFF.2,9_TxEnhG1 | AWG,H1-hESC,None;AWG,HSMM,None;AWG,HSMMtube,None;AWG,LNCaP,None;AWG,MCF-7,None;AWG,NHEK,None;Duke,Fibrobl,None;Duke,Gliobla,None;Duke,H9ES,None;Duke,Ishikawa,4OHTAM_20nM_72hr;Duke,LNCaP,androgen;Duke,Osteobl,None;Duke,ProgFib,None;Duke,iPS,None;UW,H7-hESC,None;UW,NB4,None;UW,WI-38,4OHTAM_20nM_72hr | GM12878,NRSF,HudsonAlpha,None;H1-hESC,NRSF,HudsonAlpha,None;H1-hESC,NRSF,HudsonAlpha,None;HeLa-S3,NRSF,HudsonAlpha,None;HepG2,NRSF,HudsonAlpha,None;K562,NRSF,HudsonAlpha,None;PFSK-1,NRSF,HudsonAlpha,None;U87,NRSF,HudsonAlpha,None | . | CTCF_disc1;CTCF_disc4;SMC3_disc1 |
| 9 | 137359464 | rs10776935 | A | G | 0.53 | 0.19 | 0.28 | 0.23 | . | . | . | IPS.DF19,9_TxEnhG1 | . | . | . | AP-1_disc10;CAC-binding-protein;Egr-1_known2;Irf_disc4;MAZ;MAZR;MZF1::1-4_2;MZF1::1-4_3;Myc_disc10;PRDM1_known1;PU.1_disc3;PU.1_known2;Pou2f2_disc2;SP1_disc3;SP1_known1;SP1_known2;SRF_disc2;STAT_disc7;Sp4;TFII-I;UF1H3BETA;WT1;ZNF263_disc1;Zfp281;Znf143_disc3 |
| 9 | 137359635 | rs10776936 | G | A | 0.63 | 0.2 | 0.28 | 0.24 | . | . | . | IPS.DF19,9_TxEnhG1;ST.SMUS28,14_Enh | . | . | . | CDP_7;GATA_known10;GATA_known4;Pax-2_1 |
| 9 | 137359689 | rs3118557 | C | T | 0.47 | 0.7 | 0.67 | 0.73 | . | . | . | IPS.DF19,9_TxEnhG1;ST.SMUS28,14_Enh | . | . | . | . |
| 9 | 137360124 | rs59730563 | A | C | 0.1 | 0.0028 | 0.04 | 0.0026 | . | . | . | ST.SMUS28,13_EnhA;H1.BMP4DT,11_EnhWk1 | Duke,Fibrobl,None;Duke,Osteobl,None | . | . | Ets_disc7;Ik-1_1;Irf_disc5;PU.1_disc3;RBP-Jkappa_2 |
| 9 | 137360240 | rs3132278 | T | C | 0.53 | 0.82 | 0.74 | 0.92 | . | . | . | ST.SMUS28,13_EnhA;H1.BMP4DT,11_EnhWk1;COL.SMUS,12_EnhWk2;R.SMUS,12_EnhWk2;PFF.2,15_EnhP | Duke,Fibrobl,None | . | . | Cdx2_1;Foxl1_1;Hoxa9;Hoxb9 |
| 9 | 137360329 | rs3118558 | A | T | 0.16 | 0.64 | 0.49 | 0.69 | . | . | . | ST.SMUS28,13_EnhA;H1.BMP4DT,11_EnhWk1;COL.SMUS,12_EnhWk2;R.SMUS,12_EnhWk2;PFF.2,15_EnhP | . | . | . | CTCF_disc10;Ets_known1;TCF12_known1 |
| 9 | 137360659 | rs7021222 | C | T | 0.22 | 0.01 | 0 | 0 | . | . | ST.SMUS28,2_TssF;COL.SMUS,6_TssD2;R.SMUS,6_TssD2 | H1.BMP4DT,11_EnhWk1;PFF.2,15_EnhP;COL.MUC32,12_EnhWk2;DUO.SMUS,12_EnhWk2;HRT.FE,12_EnhWk2;H1.DMSC,11_EnhWk1;ADI.MSC,11_EnhWk1;ADI.NUC,15_EnhP;LV,11_EnhWk1;SK.MUS,15_EnhP;SK.MUS62,15_EnhP;PFF.1,15_EnhP;SK.MUS63,15_EnhP;MSC.ADIPC,13_EnhA | . | . | . | E2F_disc2;EBF_known3;Egr-1_disc3;PU.1_known2 |
| 9 | 137360669 | rs3118559 | C | T | 0.45 | 0.67 | 0.56 | 0.74 | . | . | ST.SMUS28,2_TssF;COL.SMUS,6_TssD2;R.SMUS,6_TssD2 | H1.BMP4DT,11_EnhWk1;PFF.2,15_EnhP;COL.MUC32,12_EnhWk2;DUO.SMUS,12_EnhWk2;HRT.FE,12_EnhWk2;H1.DMSC,11_EnhWk1;ADI.MSC,11_EnhWk1;ADI.NUC,15_EnhP;LV,11_EnhWk1;SK.MUS,15_EnhP;SK.MUS62,15_EnhP;PFF.1,15_EnhP;SK.MUS63,15_EnhP;MSC.ADIPC,13_EnhA | . | . | . | EBF_known3 |
| 9 | 137360701 | rs3118560 | C | T | 0.54 | 0.69 | 0.56 | 0.75 | . | . | ST.SMUS28,2_TssF;COL.SMUS,6_TssD2;R.SMUS,6_TssD2 | H1.BMP4DT,11_EnhWk1;PFF.2,15_EnhP;COL.MUC32,12_EnhWk2;DUO.SMUS,12_EnhWk2;HRT.FE,12_EnhWk2;H1.DMSC,11_EnhWk1;ADI.MSC,11_EnhWk1;ADI.NUC,15_EnhP;LV,11_EnhWk1;SK.MUS,15_EnhP;SK.MUS62,15_EnhP;PFF.1,15_EnhP;SK.MUS63,15_EnhP;MSC.ADIPC,13_EnhA | . | . | . | BDP1_disc1;EBF_disc2 |
| 9 | 137360790 | rs4842228 | T | A,G | 0.45 | 0.67 | 0.56 | 0.73 | . | . | ST.SMUS28,2_TssF;COL.SMUS,6_TssD2;R.SMUS,6_TssD2 | H1.BMP4DT,11_EnhWk1;PFF.2,15_EnhP;COL.MUC32,12_EnhWk2;DUO.SMUS,12_EnhWk2;HRT.FE,12_EnhWk2;H1.DMSC,11_EnhWk1;ADI.MSC,11_EnhWk1;ADI.NUC,15_EnhP;LV,11_EnhWk1;SK.MUS,15_EnhP;SK.MUS62,15_EnhP;PFF.1,15_EnhP;SK.MUS63,15_EnhP;MSC.ADIPC,13_EnhA | Duke,PanIsletD,None | . | . | . |
| 9 | 137360936 | rs4842229 | A | G | 0.33 | 0.78 | 0.73 | 0.88 | . | . | ST.SMUS28,2_TssF;DUO.SMUS,6_TssD2 | H1.BMP4DT,11_EnhWk1;PFF.2,15_EnhP;HRT.FE,12_EnhWk2;ADI.NUC,15_EnhP;LV,11_EnhWk1;SK.MUS,15_EnhP;SK.MUS62,15_EnhP;PFF.1,15_EnhP;SK.MUS63,15_EnhP;MSC.ADIPC,13_EnhA;LNG.FE,11_EnhWk1;COL.MUC32,15_EnhP;ADI.MSC,13_EnhA;COL.SMUS,11_EnhWk1;R.SMUS,11_EnhWk1;R.MUC29,14_Enh | AWG,HSMMtube,None;AWG,MCF-7,None;Duke,AoSMC,None;Duke,FibroP,None;Duke,Stellate,None;UW,AG09309,None;UW,AG09319,None;UW,AG10803,None;UW,BJ,None;UW,HAc,None;UW,HCF,None;UW,HCM,None;UW,HConF,None;UW,HFF,None;UW,HFF-Myc,None;UW,HPAF,None;UW,NH-A,None;UW,NHLF,None;UW,NT2-D1,None | . | . | DMRT5;Evi-1_5;RFX5_known2;RFX5_known4 |
| 9 | 137361379 | rs3132279 | C | T | 0.2 | 0.64 | 0.52 | 0.7 | . | . | PFF.2,1_TssP;GAS,4_TssWk | H1.BMP4DT,11_EnhWk1;PFF.1,15_EnhP;SK.MUS63,15_EnhP;MSC.ADIPC,13_EnhA;ADI.MSC,13_EnhA;COL.SMUS,11_EnhWk1;R.SMUS,11_EnhWk1;R.MUC29,14_Enh;IMR90,12_EnhWk2;SK.MUS62,14_Enh;ST.SMUS28,13_EnhA;DUO.SMUS,11_EnhWk1 | . | . | . | BDP1_disc3;PU.1_disc3;STAT_disc7 |
| 9 | 137362155 | rs12237220 | G | C | 0.11 | 0.17 | 0.24 | 0.21 | . | . | . | ST.SMUS28,14_Enh | . | . | . | . |
| 9 | 137362222 | rs71483224 | T | G | 0.47 | 0.71 | 0.67 | 0.8 | . | . | . | ST.SMUS28,14_Enh | . | . | . | Esr2 |
| 9 | 137362805 | rs112112878 | G | C | 0.27 | 0.04 | 0.07 | 0.05 | . | . | . | ST.SMUS28,14_Enh | . | . | . | HNF4_disc1;Nrf-2_2;PRDM1_disc1 |
| 9 | 137362894 | rs9409932 | T | C | 0.4 | 0.66 | 0.52 | 0.71 | . | . | . | ST.SMUS28,14_Enh | . | . | . | Esr2;GR_disc5 |
| 9 | 137362905 | rs9410015 | A | G | 0.38 | 0.64 | 0.54 | 0.71 | . | . | . | ST.SMUS28,14_Enh | . | . | . | Ik-3;NF-kappaB_disc2 |
| 9 | 137363199 | rs111495363 | C | T | 0.18 | 0.02 | 0 | 0 | . | . | . | ST.SMUS28,14_Enh | . | . | . | . |
| Region chr9_137417100-137426400 | | | |  |  |  |  |  |  |  |  |  |  |  |  |  |
| 9 | 137417212 | rs76917123 | G | A | 0.05 | 0.0028 | 0 | 0 | . | . | . | ADI.MSC,11_EnhWk1;H1.BMP4DT,11_EnhWk1;PFF.1,15_EnhP;SK.MUS63,15_EnhP;MSC.ADIPC,14_Enh | AWG,HMEC,None;Duke,Osteobl,None | . | . | BCL_disc9;EBF_disc2;ERalpha-a_known4;Hand1_2;YY1_disc4 |
| 9 | 137417305 | rs7037930 | A | G | 0.78 | 0.8 | 0.83 | 0.67 | . | . | . | ADI.MSC,11_EnhWk1;H1.BMP4DT,11_EnhWk1;PFF.1,15_EnhP;SK.MUS63,15_EnhP;MSC.ADIPC,14_Enh | AWG,HMEC,None;Duke,Osteobl,None | . | . | Roaz_2 |
| 9 | 137417384 | rs6537910 | A | C | 0.78 | 0.8 | 0.83 | 0.67 | . | . | . | ADI.MSC,11_EnhWk1;H1.BMP4DT,11_EnhWk1;PFF.1,15_EnhP;SK.MUS63,15_EnhP;MSC.ADIPC,14_Enh | Duke,iPS,None | . | . | GCM;Pax-5_known3 |
| 9 | 137418902 | rs9409901 | C | A | 0.5 | 0.33 | 0.39 | 0.26 | . | NHLF,6_Weak_Enhancer;HSMM,7_Weak_Enhancer | PFF.1,6_TssD2;ADI.MSC,2_TssF;R.SMUS,4_TssWk;SK.MUS63,1_TssP;ST.SMUS28,4_TssWk | SK.MUS,15_EnhP;ADI.NUC,15_EnhP;CHON.BMMSC,13_EnhA;GAS,14_Enh;MSC.ADIPC,13_EnhA;LV,14_Enh;MUS.SC,12_EnhWk2;PFF.2,11_EnhWk1;SK.MUS62,14_Enh;LNG.FE,12_EnhWk2;BM.MSC,11_EnhWk1 | Duke,8988T,None | . | . | BCL_disc6;NF-kappaB_known1;NF-kappaB_known6;SREBP_known3;ZBTB7A_known1 |
| 9 | 137419411 | rs3118584 | C | T | 0.42 | 0.33 | 0.41 | 0.26 | . | . | . | . | . | . | . | . |
| 9 | 137419818 | rs2182640 | C | T | 0.5 | 0.33 | 0.39 | 0.26 | . | . | . | . | Duke,Melano,None | . | . | . |
| 9 | 137420521 | rs3118586 | A | G | 0.89 | 0.81 | 0.92 | 0.65 | . | . | . | . | . | . | . | . |
| 9 | 137421333 | rs3118587 | T | G | 0.5 | 0.33 | 0.39 | 0.26 | . | . | . | . | . | . | . | Evi-1_5;GATA_known1 |
| 9 | 137421666 | rs3118588 | A | G | 0.5 | 0.33 | 0.39 | 0.26 | . | . | . | . | . | . | . | Hic1_3 |
| 9 | 137422319 | rs2148702 | G | A | 0.5 | 0.33 | 0.39 | 0.26 | . | . | . | . | . | . | . | RREB-1_2;TCF12_disc4 |
| 9 | 137422382 | rs3132323 | T | C | 0.89 | 0.81 | 0.92 | 0.65 | . | . | . | . | . | . | . | CCNT2_disc1;CTCF_disc4;GATA_known10 |
| 9 | 137422484 | rs3118589 | A | G | 0.82 | 0.78 | 0.83 | 0.64 | . | . | . | . | . | . | . | . |
| 9 | 137423393 | rs3118590 | G | A | 0.83 | 0.82 | 0.83 | 0.69 | . | . | . | MSC.ADIPC,14_Enh;ADI.MSC,13_EnhA | Duke,GM12892,None;Duke,Osteobl,None | . | . | AP-1_disc10;EBF_known1;Klf4;MAZ;MAZR;Myc_disc10;NERF1a;PU.1_disc3;Rad21_disc10;Rad21_disc6;SP1_disc3;SP1_known1;SP1_known2;UF1H3BETA |
| 9 | 137423451 | rs1555951 | G | A | 0.4 | 0.31 | 0.39 | 0.25 | . | . | . | MSC.ADIPC,14_Enh;ADI.MSC,13_EnhA | . | AG09319,CTCF,UW,None | . | Rad21_disc5 |
| 9 | 137423497 | rs3118591 | C | G | 0.9 | 0.85 | 0.92 | 0.69 | . | . | . | MSC.ADIPC,14_Enh;ADI.MSC,13_EnhA | UW,AG09309,None;UW,AG10803,None;UW,HMF,None;UW,HVMF,None | AG09309,CTCF,UW,None;AG09319,CTCF,UW,None;AG10803,CTCF,UW,None;AoAF,CTCF,UW,None;HMF,CTCF,UW,None | . | CHOP::CEBPalpha;GZF1 |
| 9 | 137424054 | rs3132322 | T | C | 0.52 | 0.36 | 0.39 | 0.3 | . | . | . | MSC.ADIPC,14_Enh;ADI.MSC,13_EnhA;DUO.MUC61,12_EnhWk2 | AWG,LNCaP,None;Duke,8988T,None | . | . | CTCF_disc10 |
| 9 | 137424444 | rs9409902 | T | A | 0.54 | 0.36 | 0.39 | 0.3 | . | . | . | ADI.MSC,14_Enh | . | . | . | HNF1_1;HNF1_6;Mef2_disc1;Mef2_known1;Mef2_known2;Mef2_known4;Mef2_known5;Mef2_known6;Ncx_2;Pou2f2_known2;Pou2f2_known8;Sox_15;Sox_2;TATA_known1 |
| 9 | 137424681 | rs7858349 | G | C | 0.96 | 0.85 | 0.91 | 0.69 | . | . | . | . | . | . | . | YY1_known5 |
| 9 | 137424804 | rs11103194 | C | T | 0.01 | 0.05 | 0 | 0.13 | . | . | . | . | . | . | . | AIRE_1;HNF4_known3;Mef2_disc1;RXR::LXR;RXRA_known6 |
| 9 | 137425039 | rs9409903 | C | T | 0.52 | 0.35 | 0.38 | 0.3 | . | . | . | . | . | . | . | AP-1_disc7;Rad21_disc7;YY1_disc4 |
| 9 | 137425049 | rs9409904 | T | C | 0.83 | 0.73 | 0.77 | 0.57 | . | . | . | . | . | . | . | AP-1_disc7 |
| 9 | 137425441 | rs3132321 | C | T | 0.51 | 0.35 | 0.39 | 0.3 | . | . | . | NCC.GED2,13_EnhA;PFF.2,14_Enh;H1.DMSC,11_EnhWk1 | . | . | . | GR_known9 |
| 9 | 137425927 | rs3132318 | C | T | 0.56 | 0.37 | 0.4 | 0.29 | . | . | H1.BMP4DT,6_TssD2 | NCC.GED2,13_EnhA;PFF.2,14_Enh;H1.DMSC,11_EnhWk1;BM.MSC,11_EnhWk1;BN.AG,12_EnhWk2;BN.ITL,12_EnhWk2;CHON.BMMSC,11_EnhWk1;IPS.DF19,11_EnhWk1;NCC.COR2,11_EnhWk1;PFF.1,15_EnhP;BN.HM150,12_EnhWk2;BN.SN,12_EnhWk2;IPS.DF6,11_EnhWk1;BN.AC,12_EnhWk2;BN.CC,12_EnhWk2;ST.SMUS28,15_EnhP;IMR90,11_EnhWk1 | Duke,Fibrobl,None;Duke,Osteobl,None;Duke,PanIsletD,None;Duke,ProgFib,None;Duke,Stellate,None;Duke,Urothelia,None;Duke,Urothelia,UT189;Duke,iPS,None;Duke,pHTE,None;UW,AG09309,None;UW,HA-h,None;UW,HAc,None;UW,HIPEpiC,None;UW,HNPCEpiC,None;UW,NB4,None;UW,NH-A,None;UW,NHLF,None | . | . | EWSR1-FLI1;Ets_disc7;GR_known3;Maf_disc2;ZBRK1 |
| 9 | 137426119 | rs3132317 | C | T | 0.01 | 0.12 | 0.06 | 0.12 | . | NHLF,7_Weak_Enhancer | BN.GM2,4_TssWk;BN.ITL,6_TssD2;BN.AG,4_TssWk;CHON.BMMSC,2_TssF;BM.MSC,6_TssD2;BN.SN,6_TssD2;BN.HM150,6_TssD2 | NCC.GED2,13_EnhA;PFF.2,14_Enh;H1.DMSC,11_EnhWk1;IPS.DF19,11_EnhWk1;NCC.COR2,11_EnhWk1;PFF.1,15_EnhP;IPS.DF6,11_EnhWk1;BN.AC,12_EnhWk2;BN.CC,12_EnhWk2;ST.SMUS28,15_EnhP;IMR90,11_EnhWk1;BN.FE0,12_EnhWk2;H1,12_EnhWk2;H1.BMP4DT,11_EnhWk1;HD.CD56MESC,11_EnhWk1;MUS.SC,12_EnhWk2 | Duke,GM19240,None;UW,HBMEC,None;UW,SKMC,None | . | . | . |
| 9 | 137426334 | rs3118593 | C | A | 0.65 | 0.37 | 0.39 | 0.29 | . | NHLF,7_Weak_Enhancer | BN.AG,4_TssWk;CHON.BMMSC,2_TssF;BM.MSC,6_TssD2;BN.SN,6_TssD2;BN.HM150,6_TssD2;BN.AC,6_TssD2;BN.CC,6_TssD2 | PFF.2,14_Enh;H1.DMSC,11_EnhWk1;IPS.DF19,11_EnhWk1;NCC.COR2,11_EnhWk1;PFF.1,15_EnhP;ST.SMUS28,15_EnhP;IMR90,11_EnhWk1;H1.BMP4DT,11_EnhWk1;HD.CD56MESC,11_EnhWk1;MUS.SC,12_EnhWk2;BN.ITL,12_EnhWk2;LNG.FE,15_EnhP;BN.GM2,12_EnhWk2;NCC.GED2,11_EnhWk1 | . | . | . | SRF_known3;TATA_known4;TATA_known5;TCF4_known2 |
| Region chr9_137511000-137519000 | | | |  |  |  |  |  |  |  |  |  |  |  |  |  |
| 9 | 137511278 | rs6537934 | T | C | 0.94 | 0.44 | 0.6 | 0.38 | . | . | . | H1.BMP4DT,12_EnhWk2;BR.MYO,14_Enh;PFF.2,11_EnhWk1 | . | . | . | AP-1_disc7 |
| 9 | 137511314 | rs7873001 | G | A | 0.21 | 0.01 | 0.04 | 0.0026 | . | . | . | H1.BMP4DT,12_EnhWk2;BR.MYO,14_Enh;PFF.2,11_EnhWk1 | . | . | . | Zic_2;Zic_3 |
| 9 | 137512483 | rs12344933 | G | A | 0.58 | 0.11 | 0.09 | 0.13 | . | HSMM,7_Weak_Enhancer;Huvec,7_Weak_Enhancer | . | PFF.2,11_EnhWk1;HD.CD56MESC,11_EnhWk1;CHON.BMMSC,11_EnhWk1;H1.BMP4DT,11_EnhWk1;MUS.SC,11_EnhWk1;IMR90,11_EnhWk1 | . | . | . | BRCA1_known2 |
| 9 | 137512641 | rs73555170 | C | T | 0.47 | 0.07 | 0.08 | 0.07 | . | HSMM,7_Weak_Enhancer;Huvec,7_Weak_Enhancer | . | PFF.2,11_EnhWk1;HD.CD56MESC,11_EnhWk1;CHON.BMMSC,11_EnhWk1;H1.BMP4DT,11_EnhWk1;MUS.SC,11_EnhWk1;IMR90,11_EnhWk1;PFF.1,15_EnhP;BM.MSC,11_EnhWk1 | AWG,HeLa-S3,None;Duke,Osteobl,None | . | . | . |
| 9 | 137513309 | rs11103435 | A | C | 0.4 | 0.04 | 0.08 | 0.01 | . | HSMM,7_Weak_Enhancer | . | HD.CD56MESC,11_EnhWk1;CHON.BMMSC,11_EnhWk1;H1.BMP4DT,11_EnhWk1;IMR90,11_EnhWk1;BM.MSC,11_EnhWk1;PFF.2,12_EnhWk2;H1.DMSC,11_EnhWk1;IPS.DF19,11_EnhWk1;BN.GM2,11_EnhWk1 | . | . | . | CCNT2_disc2;Egr-1_disc6;Egr-1_known3;Ets_disc9;Myc_disc10;Myc_disc9;Pou2f2_disc2;SP1_known1;SP1_known2;SRF_disc2;THAP1_disc1;WT1;ZBTB7A_known2;ZNF219;Zfp281;Zfp740 |
| 9 | 137513472 | rs73555178 | G | T | 0.11 | 0.01 | 0.03 | 0.0013 | . | HSMM,7_Weak_Enhancer | . | HD.CD56MESC,11_EnhWk1;CHON.BMMSC,11_EnhWk1;H1.BMP4DT,11_EnhWk1;IMR90,11_EnhWk1;BM.MSC,11_EnhWk1;H1.DMSC,11_EnhWk1;IPS.DF19,11_EnhWk1;BN.GM2,11_EnhWk1;PFF.2,15_EnhP | . | . | . | ATF3_known8;INSM1;Myc_known8;NF-kappaB_disc2;Rad21_disc5;YY1_known5 |
| 9 | 137513642 | rs28369640 | T | C | 0.22 | 0.01 | 0 | 0.0013 | . | . | . | HD.CD56MESC,11_EnhWk1;CHON.BMMSC,11_EnhWk1;H1.BMP4DT,11_EnhWk1;IMR90,11_EnhWk1;BM.MSC,11_EnhWk1;H1.DMSC,11_EnhWk1;IPS.DF19,11_EnhWk1;BN.GM2,11_EnhWk1 | . | . | . | AP-1_disc3;AP-1_known1;AP-1_known3;AP-1_known4;AP-2_disc1;BAF155_disc1;BATF_disc1;BCL_disc2;Bach1;Bach2;GR_disc2;HMGN3_disc1;Irf_disc2;KAP1_disc1;Maf_disc1;Mef2_disc3;Myc_disc3;NF-E2_disc1;NF-E2_known1;PRDM1_disc2;RXRA_disc3;STAT_disc2;TCF4_disc1;p300_disc1 |
| 9 | 137513917 | rs11103436 | A | G | 0.29 | 0.03 | 0 | 0.004 | . | . | . | HD.CD56MESC,11_EnhWk1;CHON.BMMSC,11_EnhWk1;H1.BMP4DT,11_EnhWk1;BM.MSC,11_EnhWk1;IPS.DF19,11_EnhWk1;BN.GM2,11_EnhWk1;NCC.GED2,12_EnhWk2 | . | . | . | AIRE_1;Hoxa10;Hoxa9;Hoxc10;Hoxc9;Hoxd10;Nkx6-1_1;Pdx1_1;Pou5f1_known2 |
| 9 | 137513953 | rs74350003 | C | G | 0.08 | 0.27 | 0.25 | 0.16 | . | . | . | HD.CD56MESC,11_EnhWk1;CHON.BMMSC,11_EnhWk1;H1.BMP4DT,11_EnhWk1;BM.MSC,11_EnhWk1;IPS.DF19,11_EnhWk1;BN.GM2,11_EnhWk1;NCC.GED2,12_EnhWk2 | . | . | . | ATF3_known8;Tgif1_2 |
| 9 | 137513971 | rs72772520 | G | C | 0.08 | 0.27 | 0.34 | 0.16 | . | . | . | HD.CD56MESC,11_EnhWk1;CHON.BMMSC,11_EnhWk1;H1.BMP4DT,11_EnhWk1;BM.MSC,11_EnhWk1;IPS.DF19,11_EnhWk1;BN.GM2,11_EnhWk1;NCC.GED2,12_EnhWk2 | . | . | . | HEN1_1;Pax-5_known4;Pax-6_2;SP2_disc2 |
| 9 | 137514117 | rs11103437 | C | G | 0.44 | 0.88 | 0.96 | 0.82 | . | . | . | CHON.BMMSC,11_EnhWk1;H1.BMP4DT,11_EnhWk1;BM.MSC,11_EnhWk1;IPS.DF19,11_EnhWk1;BN.GM2,11_EnhWk1;NCC.GED2,12_EnhWk2;HD.CD56MESC,14_Enh | AWG,Th1,None | . | . | Sox_13;p300_disc3 |
| 9 | 137514606 | rs7849030 | C | G | 0.2 | 0.04 | 0.04 | 0.04 | . | GM12878,7_Weak_Enhancer;HSMM,7_Weak_Enhancer | . | CHON.BMMSC,11_EnhWk1;BM.MSC,11_EnhWk1;IPS.DF19,11_EnhWk1;BN.GM2,11_EnhWk1;NCC.GED2,12_EnhWk2;HD.CD56MESC,14_Enh;NCC.COR2,12_EnhWk2;H1.BMP4DT,11_EnhWk1;HUES6,11_EnhWk1;IMR90,11_EnhWk1 | . | . | . | EWSR1-FLI1;PU.1_disc3;STAT_disc7;TATA_disc7;ZNF263_disc1 |
| 9 | 137514801 | rs57187152 | T | C | 0.34 | 0.14 | 0.15 | 0.2 | . | HSMM,7_Weak_Enhancer | . | CHON.BMMSC,11_EnhWk1;BM.MSC,11_EnhWk1;IPS.DF19,11_EnhWk1;BN.GM2,11_EnhWk1;NCC.GED2,12_EnhWk2;H1.BMP4DT,11_EnhWk1;IMR90,11_EnhWk1;H1,12_EnhWk2;PFF.1,15_EnhP;IPS.18,12_EnhWk2;BN.AG,12_EnhWk2;BN.MFL,12_EnhWk2;H1.DMSC,11_EnhWk1;BN.FE2,12_EnhWk2;HD.CD56MESC,11_EnhWk1;PFF.2,11_EnhWk1;HUES6,14_Enh;MSC.ADIPC,11_EnhWk1;ADI.MSC,12_EnhWk2 | AWG,Th1,None;Duke,Osteobl,None | . | . | Egr-1_disc4;NRSF_disc1 |
| 9 | 137514962 | rs7027374 | A | G | 0.38 | 0.35 | 0.45 | 0.17 | . | HSMM,7_Weak_Enhancer | . | CHON.BMMSC,11_EnhWk1;BM.MSC,11_EnhWk1;IPS.DF19,11_EnhWk1;BN.GM2,11_EnhWk1;NCC.GED2,12_EnhWk2;H1.BMP4DT,11_EnhWk1;IMR90,11_EnhWk1;H1,12_EnhWk2;PFF.1,15_EnhP;IPS.18,12_EnhWk2;BN.AG,12_EnhWk2;BN.MFL,12_EnhWk2;H1.DMSC,11_EnhWk1;BN.FE2,12_EnhWk2;HD.CD56MESC,11_EnhWk1;PFF.2,11_EnhWk1;HUES6,14_Enh;MSC.ADIPC,11_EnhWk1;ADI.MSC,12_EnhWk2 | Duke,Fibrobl,None;UW,BE2_C,None | . | . | XBP-1_2 |
| 9 | 137515156 | rs4262378 | A | G | 0.84 | 0.32 | 0.47 | 0.24 | . | HSMM,7_Weak_Enhancer | . | CHON.BMMSC,11_EnhWk1;BM.MSC,11_EnhWk1;BN.GM2,11_EnhWk1;H1.BMP4DT,11_EnhWk1;H1.DMSC,11_EnhWk1;BN.FE2,12_EnhWk2;HD.CD56MESC,11_EnhWk1;PFF.2,11_EnhWk1;HUES6,14_Enh;MSC.ADIPC,11_EnhWk1;ADI.MSC,12_EnhWk2;BN.HM150,12_EnhWk2;MUS.SC,11_EnhWk1 | Duke,Urothelia,UT189 | . | . | . |
| 9 | 137515158 | rs4424343 | G | A | 0.68 | 0.31 | 0.47 | 0.24 | . | HSMM,7_Weak_Enhancer | . | CHON.BMMSC,11_EnhWk1;BM.MSC,11_EnhWk1;BN.GM2,11_EnhWk1;H1.BMP4DT,11_EnhWk1;H1.DMSC,11_EnhWk1;BN.FE2,12_EnhWk2;HD.CD56MESC,11_EnhWk1;PFF.2,11_EnhWk1;HUES6,14_Enh;MSC.ADIPC,11_EnhWk1;ADI.MSC,12_EnhWk2;BN.HM150,12_EnhWk2;MUS.SC,11_EnhWk1 | Duke,Urothelia,UT189 | . | . | . |
| 9 | 137515846 | rs12342979 | G | A | 0.24 | 0.01 | 0.0017 | 0.0013 | . | HSMM,5_Strong_Enhancer | PFF.2,6_TssD2 | H1.BMP4DT,11_EnhWk1;HUES6,14_Enh;MSC.ADIPC,11_EnhWk1;ADI.MSC,12_EnhWk2;MUS.SC,11_EnhWk1;HD.CD56MESC,13_EnhA;PFF.1,13_EnhA;PFK.3,12_EnhWk2;CHON.BMMSC,13_EnhA;PFK.2,14_Enh | . | . | . | AP-4_1;AP-4_2;Foxa_disc3;GATA_disc6;LBP-1_2;NF-I_2 |
| 9 | 137516100 | rs7863156 | T | G | 0.26 | 0.07 | 0 | 0.1 | . | HSMM,5_Strong_Enhancer | PFF.2,6_TssD2 | HUES6,14_Enh;MSC.ADIPC,11_EnhWk1;ADI.MSC,12_EnhWk2;MUS.SC,11_EnhWk1;HD.CD56MESC,13_EnhA;PFF.1,13_EnhA;PFK.3,12_EnhWk2;CHON.BMMSC,13_EnhA;IMR90,11_EnhWk1;BM.MSC,11_EnhWk1;BN.ITL,14_Enh;BR.H35,12_EnhWk2;H1.BMP4DT,13_EnhA;PFK.2,13_EnhA | . | . | . | CEBPB_disc1;Hsf_disc1;Pou2f2_known1 |
| 9 | 137516625 | rs7341858 | T | C | 0.9 | 0.38 | 0.4 | 0.32 | . | NHEK,5_Strong_Enhancer;HSMM,4_Strong_Enhancer;Huvec,5_Strong_Enhancer;HMEC,6_Weak_Enhancer;NHLF,6_Weak_Enhancer | PFF.1,2_TssF;MSC.ADIPC,6_TssD2;PFF.2,2_TssF | ADI.MSC,12_EnhWk2;MUS.SC,11_EnhWk1;HD.CD56MESC,13_EnhA;PFK.3,12_EnhWk2;CHON.BMMSC,13_EnhA;BN.ITL,14_Enh;BR.H35,12_EnhWk2;H1.BMP4DT,13_EnhA;PFK.2,13_EnhA;BN.MFL,12_EnhWk2;BM.MSC,13_EnhA;IMR90,13_EnhA;HUES6,13_EnhA;IPS.DF19,11_EnhWk1;BN.AG,12_EnhWk2;BN.CC,12_EnhWk2;LNG.FE,11_EnhWk1;ES.I3,12_EnhWk2;H1.DMSC,11_EnhWk1 | AWG,Th1,None;Duke,Melano,None;Duke,RWPE1,None;Duke,Urothelia,None | . | . | TATA_disc5 |
| 9 | 137518273 | rs12345728 | G | T | 0.17 | 0.01 | 0.05 | 0 | . | NHLF,7_Weak_Enhancer;HSMM,7_Weak_Enhancer;Huvec,5_Strong_Enhancer;H1,7_Weak_Enhancer;NHEK,7_Weak_Enhancer | . | ADI.MSC,12_EnhWk2;HD.CD56MESC,13_EnhA;CHON.BMMSC,13_EnhA;PFK.2,13_EnhA;BN.MFL,12_EnhWk2;IMR90,13_EnhA;HUES6,13_EnhA;LNG.FE,11_EnhWk1;ES.I3,12_EnhWk2;H1.DMSC,11_EnhWk1;HUES64,12_EnhWk2;NCC.COR2,12_EnhWk2;NCC.GED2,12_EnhWk2;PFF.2,13_EnhA;IPS.DF19,11_EnhWk1;PFK.3,12_EnhWk2;MUS.SC,11_EnhWk1;H1,11_EnhWk1;PFF.1,13_EnhA;IPS.18,12_EnhWk2;HUES48,12_EnhWk2;IPS.20,12_EnhWk2;H1.BMP4DT,11_EnhWk1;BM.MSC,11_EnhWk1;H9,12_EnhWk2;H1.BMP4DM,14_Enh | Duke,Melano,None | . | . | Irf_known9;UF1H3BETA |
| 9 | 137518299 | rs10858263 | T | C | 0.86 | 0.41 | 0.34 | 0.29 | . | NHLF,7_Weak_Enhancer;HSMM,7_Weak_Enhancer;Huvec,5_Strong_Enhancer;H1,7_Weak_Enhancer;NHEK,7_Weak_Enhancer | . | ADI.MSC,12_EnhWk2;HD.CD56MESC,13_EnhA;CHON.BMMSC,13_EnhA;PFK.2,13_EnhA;BN.MFL,12_EnhWk2;IMR90,13_EnhA;HUES6,13_EnhA;LNG.FE,11_EnhWk1;ES.I3,12_EnhWk2;H1.DMSC,11_EnhWk1;HUES64,12_EnhWk2;NCC.COR2,12_EnhWk2;NCC.GED2,12_EnhWk2;PFF.2,13_EnhA;IPS.DF19,11_EnhWk1;PFK.3,12_EnhWk2;MUS.SC,11_EnhWk1;H1,11_EnhWk1;PFF.1,13_EnhA;IPS.18,12_EnhWk2;HUES48,12_EnhWk2;IPS.20,12_EnhWk2;H1.BMP4DT,11_EnhWk1;BM.MSC,11_EnhWk1;H9,12_EnhWk2;H1.BMP4DM,14_Enh | . | . | . | RXRA_known3;RXRA_known5;SREBP_known3 |
| 9 | 137518733 | rs7865626 | C | A | 0.24 | 0.11 | 0 | 0.16 | . | H1,7_Weak_Enhancer;HSMM,6_Weak_Enhancer;Huvec,7_Weak_Enhancer | IPS.DF19,6_TssD2 | CHON.BMMSC,13_EnhA;PFK.2,13_EnhA;BN.MFL,12_EnhWk2;IMR90,13_EnhA;HUES6,13_EnhA;ES.I3,12_EnhWk2;H1.DMSC,11_EnhWk1;NCC.COR2,12_EnhWk2;PFF.2,13_EnhA;PFK.3,12_EnhWk2;MUS.SC,11_EnhWk1;H1,11_EnhWk1;PFF.1,13_EnhA;HUES48,12_EnhWk2;IPS.20,12_EnhWk2;H1.BMP4DT,11_EnhWk1;BM.MSC,11_EnhWk1;H1.BMP4DM,14_Enh;BN.HM150,12_EnhWk2;HD.CD56MESC,11_EnhWk1;BN.FE0,12_EnhWk2 | AWG,MCF-7,None;UW,HConF,None | HeLa-S3,CEBPB,Stanford,None;HeLa-S3,CJUN,Stanford,None;HeLa-S3,ELK4,UCD,None;HeLa-S3,GTF2F1,Stanford,None;HeLa-S3,JUND,Stanford,None;HeLa-S3,P300,Stanford,None;HeLa-S3,POL2,HudsonAlpha,None;HeLa-S3,RAD21,Stanford,None;HeLa-S3,TBP,Stanford,None | . | AP-1_known2;BATF_disc1;Bach1;CEBPD;Cphx;GATA_disc2;HNF1_3;HNF1_5;HNF1_6;HNF1_7;KAP1_disc1;Pax-8_1;Pax-8_2;RXRA_disc3;TFII-I;p300_disc2 |
| 9 | 137518798 | rs28637698 | G | A | 0.18 | 0.01 | 0.05 | 0 | . | H1,7_Weak_Enhancer;HSMM,6_Weak_Enhancer;Huvec,7_Weak_Enhancer | IPS.DF19,6_TssD2 | CHON.BMMSC,13_EnhA;PFK.2,13_EnhA;BN.MFL,12_EnhWk2;IMR90,13_EnhA;HUES6,13_EnhA;ES.I3,12_EnhWk2;H1.DMSC,11_EnhWk1;NCC.COR2,12_EnhWk2;PFF.2,13_EnhA;PFK.3,12_EnhWk2;MUS.SC,11_EnhWk1;H1,11_EnhWk1;PFF.1,13_EnhA;HUES48,12_EnhWk2;IPS.20,12_EnhWk2;H1.BMP4DT,11_EnhWk1;BM.MSC,11_EnhWk1;H1.BMP4DM,14_Enh;BN.HM150,12_EnhWk2;HD.CD56MESC,11_EnhWk1;BN.FE0,12_EnhWk2 | UW,HConF,None | HeLa-S3,CEBPB,Stanford,None;HeLa-S3,CJUN,Stanford,None;HeLa-S3,ELK4,UCD,None;HeLa-S3,GTF2F1,Stanford,None;HeLa-S3,JUND,Stanford,None;HeLa-S3,P300,Stanford,None;HeLa-S3,POL2,HudsonAlpha,None;HeLa-S3,RAD21,Stanford,None;HeLa-S3,TBP,Stanford,None | . | . |

## **Cell lines:** *8988T*-pancreas adenocarcinoma; *ADI.MSC*-Adipose Derived Mesenchymal Stem Cell Cultured Cells; *AG09309*-adult toe fibroblast; *AG09319*-gum tissue fibroblasts; *AG10803*-abdominal skin fibroblasts; *AoSMC*-aortic smooth muscle; *BE2_C*-neuroblastoma; *BJ*-skin fibroblast; *BM.MSC*-Bone Marrow Derived Mesenchymal Stem Cell Cultured Cells; *BN.AC*-Brain Anterior Caudate; *BN.AG*-Brain Angular Gyrus; *BN.CC*-Brain Cingulate Gyrus; *BN.GM2*-Brain Germinal Matrix.Donor HuFGM02; *BN.HM150*-Brain Hippocampus Middle.Donor 150; *BN.ITL*-Brain Inferior Temporal Lobe; *BN.SN*-Brain Substantia Nigra; *CD34+_Mobilized*-hematopoietic progenitor cells; *CD34.MBP1549*-Mobilized CD34 Primary Cells.Donor RO 01549; *CHON.BMMSC*-Chondrocytes from Bone Marrow Derived Mesenchymal Stem Cell Cultured Cells; *CMK*-acute megakaryocytic leukemia; *COL.SMUS*-Colon Smooth Muscle; *DUO.SMUS*-Duodenum Smooth Muscle; Fibrobl-child fibroblast; FibroP- fibroblasts taken from individuals with Parkinson's disease; *GAS*-Gastric; Gliobla-glioblastoma; *GM12878*-B-lymphocyte, lymphoblastoid; *GM19240*-B-lymphocyte, lymphoblastoid; *H1*-H1 Cell Line; *H1.BMP4DT*-H1 BMP4 Derived Trophoblast Cultured Cells; *H1.DMSC*-H1 Derived Mesenchymal Stem Cells; *H1-hESC*-embryonic stem cells embryonic stem cell (hESC) H9; *H7-hESC*-undifferentiated embryonic stem cells; *H9ES*-endometrial adenocarcinoma; *HAc*-astrocytes-cerebellar; *HA-h*-astrocytes-hippocampal; *HBMEC*-brain microvascular endothelial cells; *HCF*-cardiac fibroblasts; *HCM*-cardiac myocytes; *HConF*-conjunctival fibroblast; *HeLa-S3*-cervical carcinoma; *Hepatocytes*-primary hepatocytes; *HepG2*-hepatocellular carcinoma; *HFF*-foreskin fibroblast; *HFF-Myc*-foreskin fibroblast cells expressing canine cMyc; *HMEC*-mammary epithelial cells; *HMF*-mammary fibroblasts; *HIPEpiC*-iris pigment epithelial cells; *HNPCEpiC*-non-pigment ciliary epithelial cells; *HPAF*-pulmonary artery fibroblasts; *HSMM*-skeletal muscle myoblasts; *HSMMtube*-skeletal muscle myotubes differentiated from the HSMM cell line; *Huvec*-umbilical vein endothelial cells; *HVMF*-villous mesenchymal fibroblast cells; *iPS*-induced pluripotent stem cell derived from skin fibroblast; *IPS.DF19*-iPS DF 19.11 Cell Line; *Jurkat*-T lymphoblastoid derived from an acute T cell leukemia; *K562*-leukemia; *LNCaP*-prostate adenocarcinoma; *MCF-7*-mammary gland, adenocarcinoma; *Melano*-epidermal melanocytes; *Monocytes-CD14+_RO01746*-CD14-positive cells from human leukapheresis production; *MSC.ADIPC*-Mesenchymal Stem Cell Derived Adipocyte Cultured Cells; *NB4*-acute promyelocytic leukemia; *NH-A*-astrocytes; *NHEK*-epidermal keratinocytes; *NHLF*-lung fibroblasts; *Osteobl*-osteoblasts (NHOst); *NT2-D1*-malignant pluripotent embryonal carcinoma; *PanIsletD*-pancreatic islets; *PFF.1*-Penis Foreskin Fibroblast Primary Cells.Donor skin01; *PFF.2*-Penis Foreskin Fibroblast Primary Cells.Donor skin02; *pHTE*-primary tracheal epithelial cells; *ProgFib*-fibroblasts, Hutchinson-Gilford progeria syndrome; *R.SMUS*-Rectal Smooth Muscle; *RWPE1*-prostate epithelial; *SKMC*-Skeletal Striated Muscle Cells from M. pectoralis / Mm. intercostales; *SK.MUS63*-Skeletal Muscle.Donor 63; *SPL*-Spleen; *ST.SMUS28*-Stomach Smooth Muscle.Donor 28; *Stellate*-hepatic stellate cells; *TH1*-primary Th1 T cells; *Urothelia*-urothelial cells; *WI-38*-embryonic lung fibroblast cells.

The GTEx expression profiles in various human tissues for genes *RXRA* and *RP11-473E2.4* are displayed in Fig U.


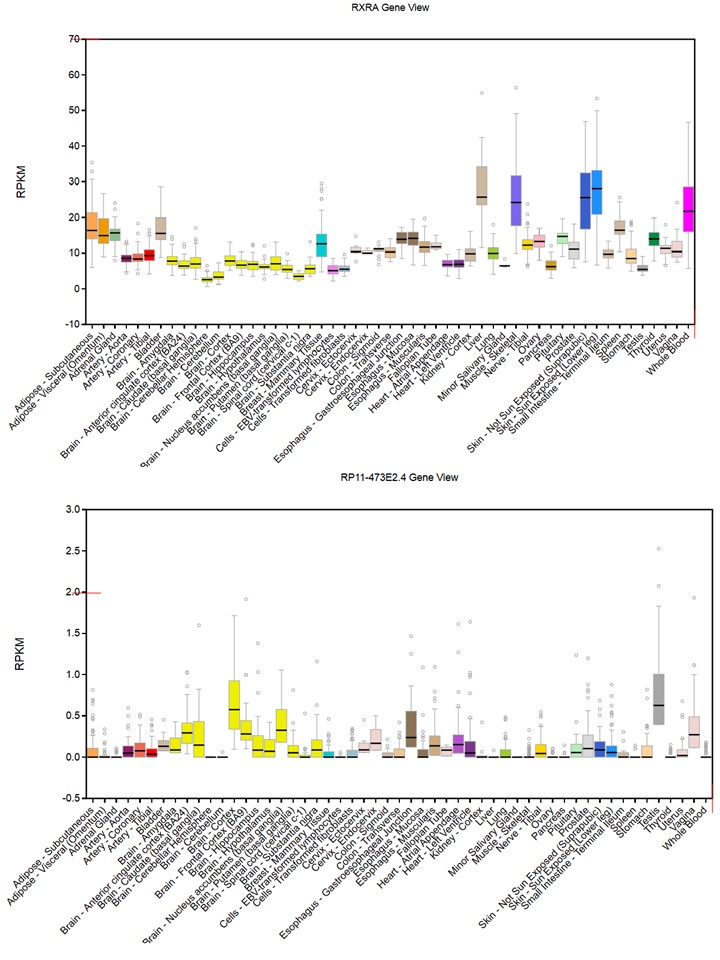


**Fig U. Expression profiles of *RXRA* and *RP11-473E2.4* genes in various human tissues, according to GTEx portal.**

Three other lncRNAs surround the *RXRA* gene, but are expressed in just one or two tissues (Figs V-W) and at low levels, and seem not to have any SNP related with dengue infection.


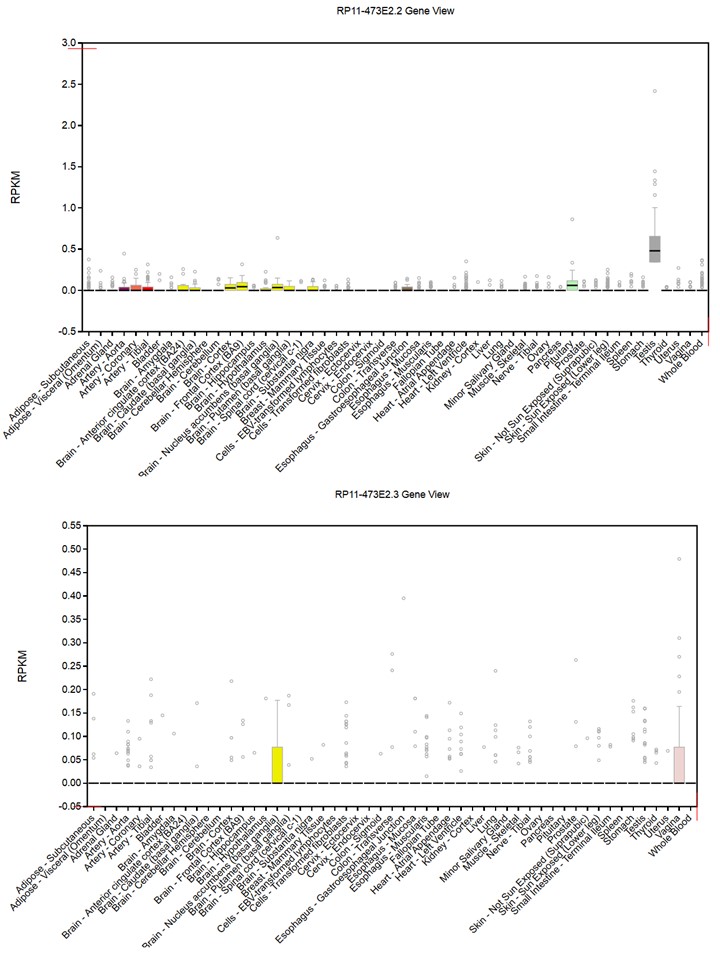


**Fig V. Expression profiles of *RP11-473E2.2* and *RP11-473E2.3* genes in various human tissues, according to GTEx portal.**


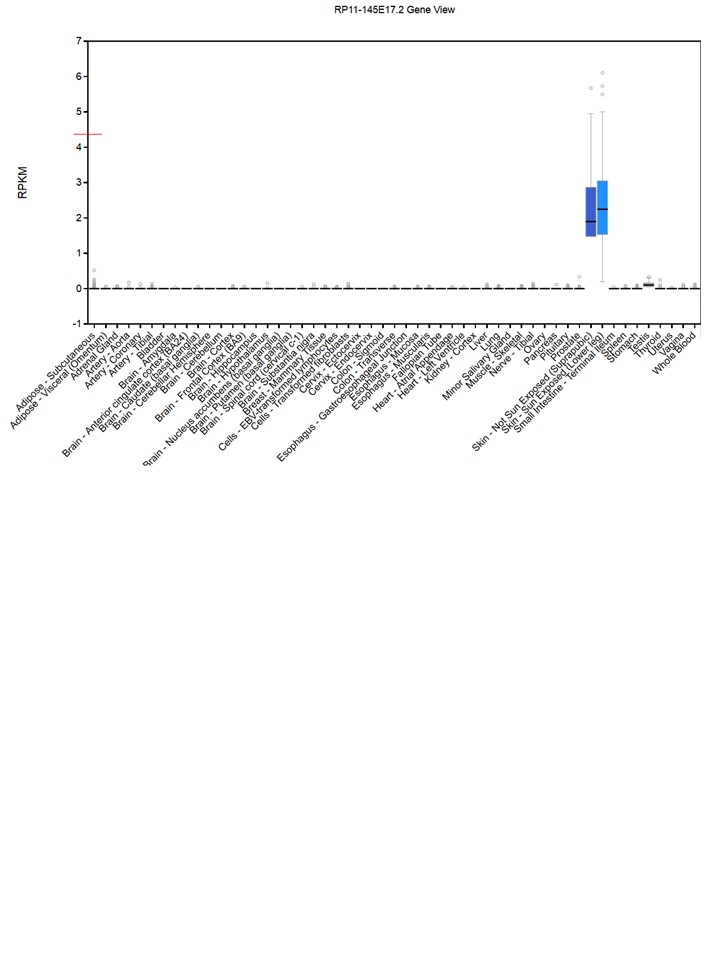


**Fig W. Expression profile of *RP11-145E17.2* gene in various human tissues, according to GTEx portal.**

We also performed an association tests for the whole *RXRA-COL5A1* region in the 1000 Genomes transcriptome derived from blood cells. First, we compared Africans having low (n=6; lower than 10 RPKM) and high (n=8; higher than 20 RPKM) *RXRA* expression; and second, we did the same comparison in Europeans (n=42 and n=39, respectively). The results are reported in Tables Q-R, and show that several SNPs surrounding *RXRA* gene attain significant p-values between low and high profiles of *RXRA* expression, in a higher number in Africans than in Europeans.

**Table Q. Significant p-values (1% level) of the association test in 1000 Genomes Project Africans with low and high *RXRA* expression.**

| Chr | SNP | BP | Allele | Low RXRA expression | High RXRA expression | Other allele | CHISQ | P | OR |
| --- | --- | --- | --- | --- | --- | --- | --- | --- | --- |
| 9 | rs114104051 | 137488180 | T | 0.5833 | 0 | C | 12.44 | 0.0004192 | NA |
| 9 | rs4842136 | 137489483 | G | 0.5833 | 0 | A | 12.44 | 0.0004192 | NA |
| 9 | rs4240705 | 137311400 | A | 0.08333 | 0.6875 | G | 10.22 | 0.001389 | 0.04132 |
| 9 | rs10745382 | 137485204 | A | 0.5 | 0 | G | 10.18 | 0.001418 | NA |
| 9 | rs10735245 | 137485536 | T | 0.5 | 0 | C | 10.18 | 0.001418 | NA |
| 9 | rs7859903 | 137486263 | A | 0.5 | 0 | G | 10.18 | 0.001418 | NA |
| 9 | rs10858255 | 137486496 | C | 0.5 | 0 | A | 10.18 | 0.001418 | NA |
| 9 | rs7867092 | 137486860 | T | 0.5 | 0 | C | 10.18 | 0.001418 | NA |
| 9 | rs7863506 | 137486891 | A | 0.5 | 0 | G | 10.18 | 0.001418 | NA |
| 9 | rs77267695 | 137487774 | T | 0.5 | 0 | C | 10.18 | 0.001418 | NA |
| 9 | rs112112878 | 137362805 | C | 0.5833 | 0.0625 | G | 9.115 | 0.002536 | 21 |
| 9 | rs3927491 | 137313719 | A | 0 | 0.5 | C | 8.4 | 0.003752 | 0 |
| 9 | rs139318474 | 137314456 | A | 0 | 0.5 | G | 8.4 | 0.003752 | 0 |
| 9 | rs6583658 | 137256404 | G | 0.4167 | 0 | A | 8.116 | 0.004388 | NA |
| 9 | rs7389067 | 137491462 | A | 0.4167 | 0 | C | 8.116 | 0.004388 | NA |
| 9 | rs12351482 | 137261632 | T | 0.5 | 0.0625 | C | 7 | 0.008151 | 15 |
| 9 | rs58403320 | 137261840 | A | 0.5 | 0.0625 | G | 7 | 0.008151 | 15 |
| 9 | rs11787956 | 137262107 | C | 0.5 | 0.0625 | T | 7 | 0.008151 | 15 |
| 9 | rs11185661 | 137262116 | T | 0.5 | 0.0625 | C | 7 | 0.008151 | 15 |
| 9 | rs11185662 | 137262119 | C | 0.5 | 0.0625 | T | 7 | 0.008151 | 15 |
| 9 | rs62576338 | 137262663 | C | 0.5 | 0.0625 | T | 7 | 0.008151 | 15 |
| 9 | rs56239054 | 137264018 | A | 0.5 | 0.0625 | C | 7 | 0.008151 | 15 |
| 9 | rs67022788 | 137264042 | C | 0.5 | 0.0625 | T | 7 | 0.008151 | 15 |
| 9 | rs11185663 | 137265276 | C | 0.5 | 0.0625 | T | 7 | 0.008151 | 15 |
| 9 | rs34463468 | 137267991 | C | 0.5 | 0.0625 | T | 7 | 0.008151 | 15 |
| 9 | rs34525966 | 137268044 | T | 0.5 | 0.0625 | C | 7 | 0.008151 | 15 |
| 9 | rs35774225 | 137268063 | A | 0.5 | 0.0625 | G | 7 | 0.008151 | 15 |
| 9 | rs34448439 | 137268107 | A | 0.5 | 0.0625 | G | 7 | 0.008151 | 15 |
| 9 | rs62576341 | 137268339 | C | 0.5 | 0.0625 | A | 7 | 0.008151 | 15 |
| 9 | rs67816242 | 137268441 | T | 0.5 | 0.0625 | C | 7 | 0.008151 | 15 |
| 9 | rs34863049 | 137268777 | C | 0.5 | 0.0625 | G | 7 | 0.008151 | 15 |
| 9 | rs62576344 | 137268915 | T | 0.5 | 0.0625 | C | 7 | 0.008151 | 15 |
| 9 | rs11103437 | 137514117 | G | 0.5 | 0.0625 | C | 7 | 0.008151 | 15 |
| 9 | rs62571282 | 137380951 | A | 0.75 | 0.25 | G | 6.892 | 0.008657 | 9 |
| 9 | rs11103846 | 137381366 | C | 0.75 | 0.25 | T | 6.892 | 0.008657 | 9 |
| 9 | rs11103414 | 137491435 | G | 0.75 | 0.25 | A | 6.892 | 0.008657 | 9 |
| 9 | rs3118594 | 137427625 | G | 0.08333 | 0.5625 | A | 6.857 | 0.008827 | 0.07071 |

**Table R. Significant p-values (1% level) of the association test in 1000 Genomes Project Europeans with low and high *RXRA* expression.**

| Chr | SNP | BP | Allele | Low RXRA expression | High RXRA expression | Other allele | CHISQ | P | OR |
| --- | --- | --- | --- | --- | --- | --- | --- | --- | --- |
| 9 | rs34798391 | 137299790 | G | 0.2439 | 0.0641 | A | 9.802 | 0.001743 | 4.71 |
| 9 | rs111770868 | 137508744 | C | 0.08537 | 0 | G | 6.963 | 0.00832 | NA |
| 9 | rs72772513 | 137510436 | T | 0.08537 | 0 | C | 6.963 | 0.00832 | NA |
| 9 | rs72772516 | 137510565 | A | 0.08537 | 0 | G | 6.963 | 0.00832 | NA |
| 9 | rs72772517 | 137511362 | A | 0.08537 | 0 | G | 6.963 | 0.00832 | NA |
| 9 | rs10858266 | 137564762 | T | 0.3293 | 0.1538 | C | 6.673 | 0.009786 | 2.7 |

*1.9- GSEA analysis*

We checked in ingenuity database (<https://targetexplorer.ingenuity.com/index.htm>) and literature for pathways were RXRA and OBSPL10 genes could play a role and detected the LXR/RXR activation pathway. This pathway is represented in Fig X.


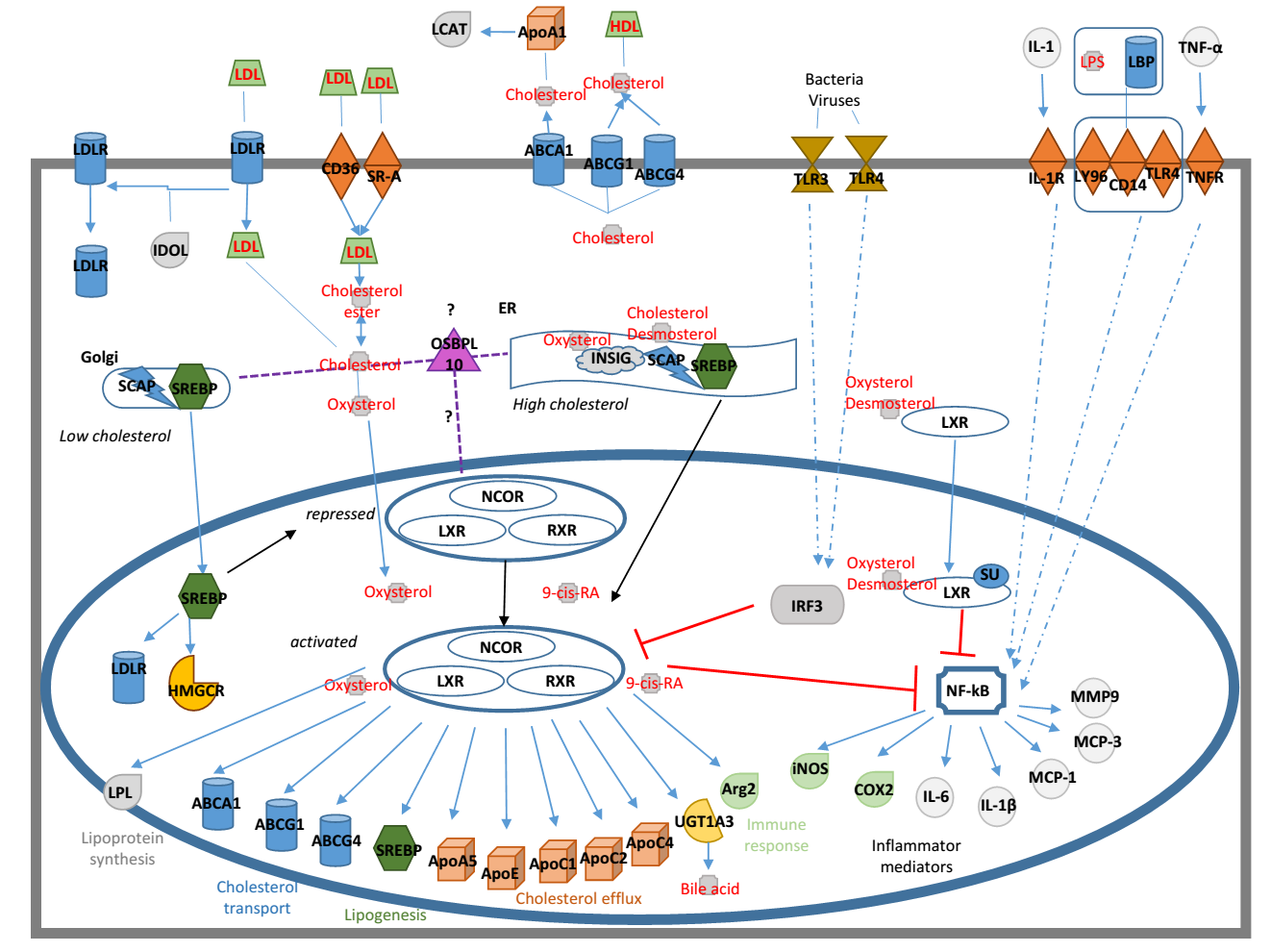


**Fig 24. The LXR/RXR activation pathway in macrophages.** Englobing the lipid metabolism, LXR/RXR activation and NF-kB activation. Information was collected from the Ingenuity database (<https://targetexplorer.ingenuity.com/index.htm>) and publications cited in the Discussion section [[11](#_ENREF_11), [12](#_ENREF_12)]. Red lines with block in the end mean inhibition; arrows mean activation. The precise mechanism by which *OSBPL10* is involved in the transport of lipids between membranous organelles and as signal detector of cholesterol or oxysterols is still under investigation.

We split the pathway into three gene sets, lipid metabolism, LXR/RXR activation and NF-kB activation as indicated in Table S, and used GSEA [[13](#_ENREF_13)] to assess the statistical significance of their enrichment score. Results of the GSEA analyses are reported in the main text, and Fig Y reports the comparison between DF and convalescents, as well as DHF and convalescents, while Fig Z displays the comparisons against controls.


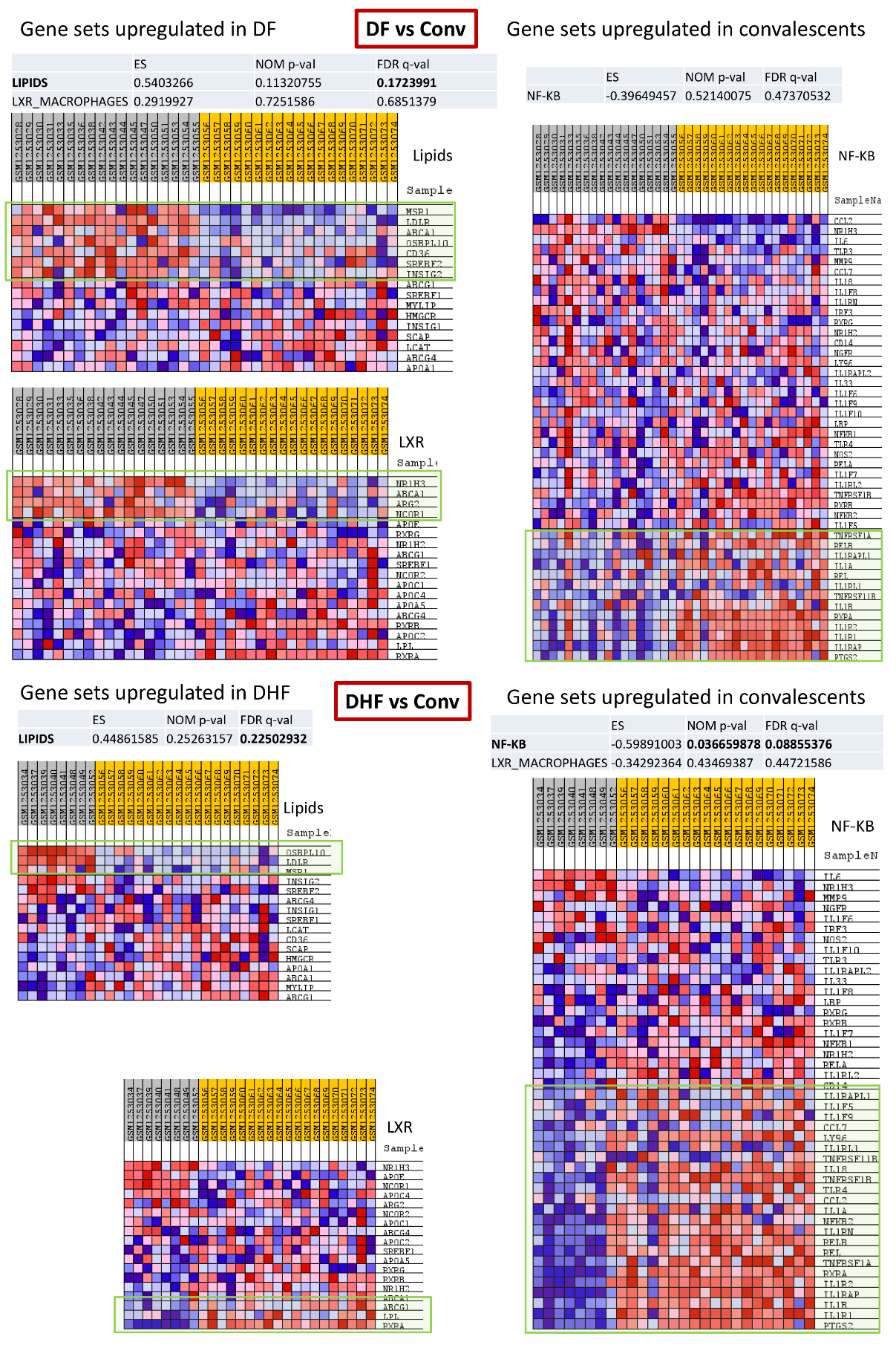


**Fig Y. GSEA analysis in DF vs convalescent subjects, and DHF vs convalescent subjects in the Thai transcriptome dataset [**[**6**](#_ENREF_6)**].**

ES is the enrichment score, reflecting the degree to which a gene set is overrepresented at the top or bottom of a ranked list of genes. NES is the normalized enrichment score, accounting for differences in gene set size, in correlations between gene sets and the expression dataset. FDR is the false discovery rate, the estimated probability that a gene set with a given NES represents a false positive finding (FDR<25%, meaning that the result is valid 3 out of 4 times, are highlighted in bold). The genes identified as up-regulated in each test are marked by the green shadow.

**Table S. List of genes included in the three gene sets used in GSEA.**

| **Lipids** | **LXR/RXR** | **NF-kB** |
| --- | --- | --- |
| ABCA1 | ABCA1 | CCL2 |
| ABCG1 | ABCG1 | CCL7 |
| ABCG4 | ABCG4 | CD14 |
| APOA1 | APOA5 | IL18 |
| CD36 | APOC1 | IL1A |
| HMGCR | APOC2 | IL1B |
| INSIG1 | APOC4 | IL1B |
| INSIG2 | APOE | IL1F10 |
| LCAT | ARG2 | IL1F5/IL36RN |
| LDLR | LPL | IL1F6/IL36A |
| MSR1 | NCOR1 | IL1F7/IL37 |
| MYLIP | NCOR2 | IL1F8/IL36B |
| OSBPL10 | NR1H2/LXRB | IL1F9/IL36G |
| SCAP | NR1H3/LXRA | IL1R1 |
| SREBF1 | RXRA | IL1R2 |
| SREBF2 | RXRB | IL1RAP |
|  | RXRG | IL1RAPL1 |
|  | SREBF1 | IL1RAPL2 |
|  | UGT1A3 | IL1RL1 |
|  |  | IL1RL2 |
|  |  | IL1RN |
|  |  | IL33 |
|  |  | IL6 |
|  |  | IRF3 |
|  |  | LBP |
|  |  | LY96 |
|  |  | MMP9 |
|  |  | NFKB1 |
|  |  | NFKB2 |
|  |  | NGFR |
|  |  | NOS2 |
|  |  | NR1H2/LXRB |
|  |  | NR1H3/LXRA |
|  |  | PTGS2 |
|  |  | REL |
|  |  | RELA |
|  |  | RELB |
|  |  | RXRA |
|  |  | RXRB |
|  |  | RXRG |
|  |  | TLR3 |
|  |  | TLR4 |
|  |  | TNFRSF11B |
|  |  | TNFRSF1A |
|  |  | TNFRSF1B |


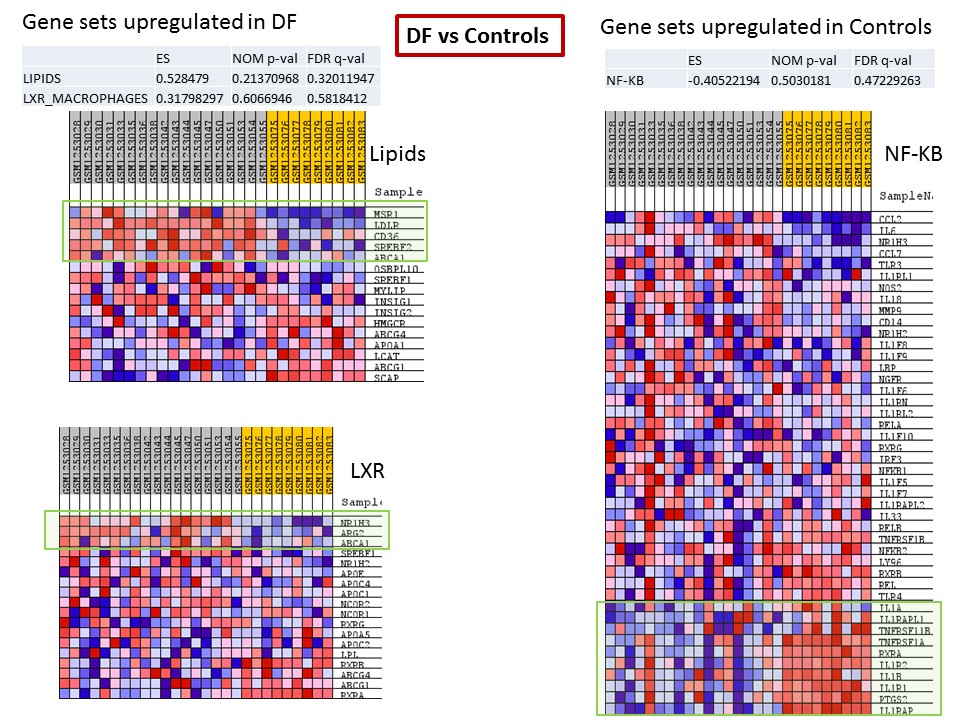


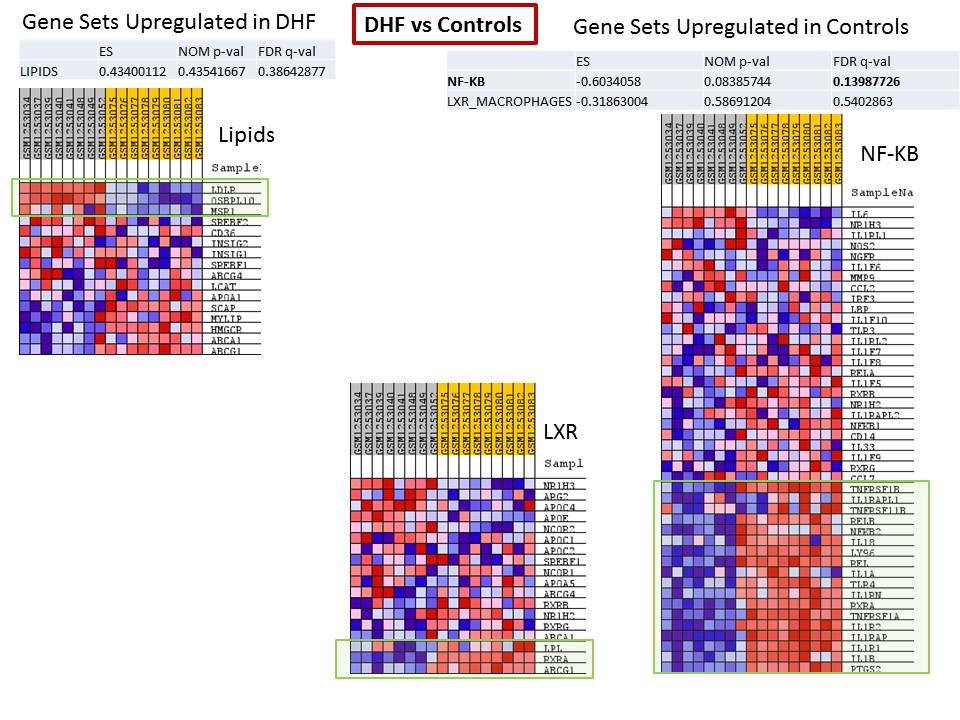


**Fig Z. GSEA analysis in DF vs controls and DHF vs controls.**

As a final GSEA test, we included the 12 *OSBP* family members in the comparison between DHF and convalescents and confirmed that the *OSBPL10* gene is the most significantly overexpressed in patients (Fig AA).


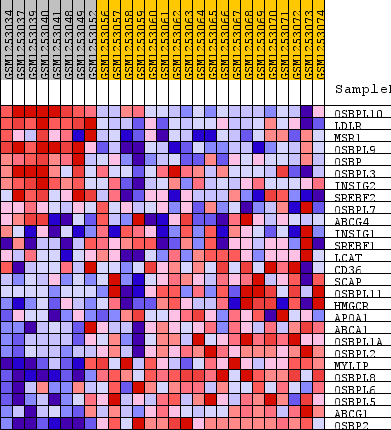


**Fig AA. GSEA analysis in DHF vs convalescents when including all 12 OSBP genes.**

*1.10- References*

1. Abecasis GR, Auton A, Brooks LD, DePristo MA, Durbin RM, Handsaker RE, et al. An integrated map of genetic variation from 1,092 human genomes. Nature. 2012;491(7422):56-65. Epub 2012/11/07. doi: 10.1038/nature11632. PubMed PMID: 23128226; PubMed Central PMCID: PMC3498066.

2. Li JZ, Absher DM, Tang H, Southwick AM, Casto AM, Ramachandran S, et al. Worldwide human relationships inferred from genome-wide patterns of variation. Science. 2008;319(5866):1100-4. Epub 2008/02/23. doi: 10.1126/science.1153717. PubMed PMID: 18292342.

3. Fritsche E, Cline JE, Nguyen NH, Scanlan TS, Abel J. Polychlorinated biphenyls disturb differentiation of normal human neural progenitor cells: clue for involvement of thyroid hormone receptors. Environmental health perspectives. 2005;113(7):871-6. Epub 2005/07/09. PubMed PMID: 16002375; PubMed Central PMCID: PMCPmc1257648.

4. Narikawa K, Nishi K, Naito Y, Yonezawa S, Mazda M, Ui-Tei K. Genome-Wide Identification and Analysis of miRNAs Complementary to Upstream Sequences of mRNA Transcription Start Sites. In: Catalano AJ, editor. Gene silencing: theory, techniques and applications. 2: Nova Science Publishers; 2010. p. 287-319.

5. Moreno-Estrada A, Gravel S, Zakharia F, McCauley JL, Byrnes JK, Gignoux CR, et al. Reconstructing the population genetic history of the Caribbean. PLoS Genet. 2013;9(11):e1003925. Epub 2013/11/19. doi: 10.1371/journal.pgen.1003925. PubMed PMID: 24244192; PubMed Central PMCID: PMC3828151.

6. Kwissa M, Nakaya HI, Onlamoon N, Wrammert J, Villinger F, Perng GC, et al. Dengue virus infection induces expansion of a CD14(+)CD16(+) monocyte population that stimulates plasmablast differentiation. Cell Host Microbe. 2014;16(1):115-27. Epub 2014/07/02. doi: 10.1016/j.chom.2014.06.001. PubMed PMID: 24981333; PubMed Central PMCID: PMC4116428.

7. Caromile LA, Oganesian A, Coats SA, Seifert RA, Bowen-Pope DF. The neurosecretory vesicle protein phogrin functions as a phosphatidylinositol phosphatase to regulate insulin secretion. J Biol Chem. 2010;285(14):10487-96. Epub 2010/01/26. doi: 10.1074/jbc.M109.066563. PubMed PMID: 20097759; PubMed Central PMCID: PMCPmc2856256.

8. Saeed M, Andreo U, Chung HY, Espiritu C, Branch AD, Silva JM, et al. SEC14L2 enables pan-genotype HCV replication in cell culture. Nature. 2015;524(7566):471-5. Epub 2015/08/13. doi: 10.1038/nature14899. PubMed PMID: 26266980.

9. Heltemes-Harris LM, Willette MJ, Vang KB, Farrar MA. The role of STAT5 in the development, function, and transformation of B and T lymphocytes. Ann N Y Acad Sci. 2011;1217:18-31. Epub 2011/02/01. doi: 10.1111/j.1749-6632.2010.05907.x. PubMed PMID: 21276004.

10. Voight BF, Kudaravalli S, Wen X, Pritchard JK. A map of recent positive selection in the human genome. PLoS Biol. 2006;4(3):e72. Epub 2006/02/24. doi: 10.1371/journal.pbio.0040072. PubMed PMID: 16494531; PubMed Central PMCID: PMC1382018.

11. Spann NJ, Glass CK. Sterols and oxysterols in immune cell function. Nature immunology. 2013;14(9):893-900. Epub 2013/08/21. doi: 10.1038/ni.2681. PubMed PMID: 23959186.

12. Zelcer N, Tontonoz P. Liver X receptors as integrators of metabolic and inflammatory signaling. The Journal of clinical investigation. 2006;116(3):607-14. Epub 2006/03/03. doi: 10.1172/jci27883. PubMed PMID: 16511593; PubMed Central PMCID: PMCPmc1386115.

13. Subramanian A, Tamayo P, Mootha VK, Mukherjee S, Ebert BL, Gillette MA, et al. Gene set enrichment analysis: a knowledge-based approach for interpreting genome-wide expression profiles. Proc Natl Acad Sci U S A. 2005;102(43):15545-50. Epub 2005/10/04. doi: 10.1073/pnas.0506580102. PubMed PMID: 16199517; PubMed Central PMCID: PMCPmc1239896.
